# Decoding 5G security: toward a hybrid threat ontology

**DOI:** 10.12688/openreseurope.16916.1

**Published:** 2024-02-19

**Authors:** R. Andrew Paskauskas

**Affiliations:** 1 Lithuanian Cybercrime Center of Excellence for Training, Research and Education (L3CE), Vilnius, 08393, Lithuania

**Keywords:** 5G Cybersecurity, Hybrid Threats, Ontology Development, RDF (Resource Description Framework), ENISA Threat Taxonomy, EU Cybersecurity Strategies, Automated Analysis in Cybersecurity, JRC Cybersecurity Taxonomy

## Abstract

The rapid deployment of 5G technology ushers in a new era of connectivity with unparalleled potential, but it also presents unprecedented security challenges.

A meticulous review of ENISA's Taxonomy is undertaken, specifically in its application to 5G networks and their cybersecurity assets. This work also evaluates the relevance of cybersecurity structures in other EU papers and ENISA reports, providing critical insights into the evolving landscape of cybersecurity.

In the context of hybrid threats, the study categorizes these multifaceted challenges using the established taxonomy. It establishes connections between ontological categories, thereby deriving an ontology that illuminates the intricate nature of hybrid threats within 5G.

The integration of the 5G vision with the TEN-T initiative for trans-European transport corridors constitutes a significant part of the research. This phase incorporates a comprehensive review of the Connecting Europe Facility (CEF) work plan, encompassing vital elements like Multi-Access Edge Computing (MEC), Network Function Virtualization (NFV), Software-Defined Networking (SDN), FOG/EDGE/CLOUD computing.

The study also delves into the intricacies of 5G cybersecurity, examining ENISA's contributions to 5G network security and risk while navigating the landscape of applicable EU and national laws, along with EU guidance. This exploration extends to cybersecurity implications within the context of the CEF funding program.

Significantly, the integration of RDF coding plays a pivotal role in aligning the developed ontology with the JRC Cybersecurity Taxonomy. This exposition represents a milestone in the field of 5G cybersecurity, as it effectively aligns a comprehensive ontology, designed to comprehend and mitigate hybrid threats in 5G networks, with the JRC Cybersecurity Taxonomy. The alignment is achieved by leveraging RDF coding techniques, which have greatly enhanced the ontology's machine-readability and interoperability.

## Part 1: Modeling the 5G cyberthreat landscape: key concepts and techniques

### Introduction

In Part 1 we take an in-depth look at developing taxonomies and ontologies for analyzing cyberthreats and vulnerabilities related to 5G networks. We begin by introducing ENISA's threat taxonomy for 5G, with categories covering various attack types, actors, and impacts. An overview of ontology development is then provided, explaining how to transform a hierarchy into a full ontology by defining classes, relationships, attributes and using RDF/OWL syntax.

Next, the ontology of hybrid threats ^i^ is examined, categorizing them based on analytical framework aspects, domains targeted, and phases of activity. Figures visually depict the ontology relationships. Focus then shifts to the cyber domain, introducing a high-level taxonomy of potential 5G cyberthreats including signaling attacks, core network threats, IoT attacks, etc. An ontology representation using RDF showcases 5G threat classes, individuals, and relationships.

Vignettes illustrate hypothetical hybrid cyberattacks on a nation's critical infrastructure across cybertheft, subversion, and sabotage vectors. A third vignette incorporates 5G-specific hybrid threats like signaling attacks, subscriber credential theft, and supply chain backdoors. Updates integrate the latest ENISA Threat Landscape report's findings.

Finally, Europol's cyberthreat analysis is incorporated. Ransomware, social engineering, intrusions, payment fraud, and child exploitation feature prominently and provide a wider context for understanding hybrid threat onslaughts.

### Exposition

In a previous work ^1^ regarding our exploration into 5G cybersecurity, I delved into the strategic integration of cybersecurity features within the design, architecture and development of 5G networks, emphasizing the need to fortify these critical infrastructures against evolving global threats. Our analysis was based on the 5G assets that formed the foundation of ENISA’s 5G design and architecture ^2^. This strategic perspective laid the groundwork for the innovative technologies and applications that the future demands. Building upon this foundation, we continue our exploration by starting with ENISA’s 5G Threat Taxonomy ^2, pp. 102–116; 124–128^ and move along a path towards an ontological approach to understanding and managing hybrid threats. Here, we consider the preliminary steps to be taken towards achieving a comprehensive ontological framework designed to fathom and mitigate the intricate landscape of hybrid threats within 5G mobile networks. We scrutinise the targets of these threats, and the actors orchestrating them, and leverage a sophisticated analytical framework. Rooted in the notion that hybrid threats encompass a dynamic blend of technological, social, and political facets, we draw inspiration from the Joint Research Council’s (JRC's) conceptual model ^3^. Our objective is to illuminate the multifaceted nature of these threats and unveil best-in-class security measures offered by ENISA to confront them. By the end of this paper, we aim to provide a deeper comprehension of hybrid threats in 5G networks, empowering organisations and policymakers to craft robust countermeasures against potential risks.

### The taxonomy of threats

A taxonomy of threats is a classification framework used to categorise and organise different types of threats in a systematic manner. In the context of cybersecurity, a taxonomy of threats for 5G networks would aim to identify and classify the various threats that could potentially impact the security of 5G infrastructures, services, and applications.

It is impossible to conduct a comprehensive literature review of the topic of ‘5G threat taxonomy’ within the confines of this open letter. Indeed, a preliminary search in Google scholar revealed close to 25,000 entries that deal with the topic. What is more, a similar search on ‘5G ontology’ revealed close to 50,000 entries. Nevertheless, it is possible to discern from sampling this literature a short list of 5G threats that would include the following categories:

*Network-based:* threats that target the 5G network infrastructure, including attacks on signaling protocols, network slicing vulnerabilities, denial-of-service (DoS) attacks, or interception of communication.
*Device and user-related:* involve threats that exploit vulnerabilities in 5G devices, such as smartphones or IoT devices, as well as threats targeting the users of these devices, including phishing attacks, social engineering, or identity theft.
*Application-level:* threats that target 5G applications and services, such as unauthorised access to sensitive data, application-level vulnerabilities, or malware specifically designed for 5G environments.
*Virtualisation and cloud-related:* threats related to the virtualised and cloud-based aspects of 5G networks, including attacks on virtualised network functions (VNFs), hypervisor vulnerabilities, or unauthorised access to cloud resources.
*Emerging threats:* encompass new and evolving threats that may arise as 5G networks continue to evolve and expand, including emerging attack vectors, zero-day vulnerabilities, or threats specific to novel 5G use cases like autonomous vehicles or smart cities.

### ENISA’S 5G taxonomy of threats

First, we consider that ENISA’S taxonomy is mapped to that of the classification scheme developed by the International Telecommunication Union (ITU); and it is also linked up with vulnerabilities presented in Annexes C to M ^2, pp. 129 – 250^. The elaborate descriptions of these relationships presented in row/column format result from the intersection between ENISA and ITU threat taxonomies.

Consider that ENISA’s Taxonomy maps to ITU’s in a general way according to the following categories ^2, p. 126^.

- Destruction of information and other resources
- Corruption or modification of information
- Theft, removal, or loss of information and other resources
- Disclosure of information
- Interruption of services

ITU-T Recommendation X.1205 is titled ‘Telecommunication security - Overview of cybersecurity’ ^ii^ and provides an overview of cybersecurity from a telecommunication security perspective. It offers a broad understanding of cybersecurity concepts and outlines the key components involved in securing telecommunication networks and systems.

Furthermore, it addresses various aspects of cybersecurity, including threat landscape analysis, risk management, security controls, incident response, and in our case, it describes a taxonomy of security threats. At a high level these include the following taxonomic components:

*Malware:* covers threats related to malicious software, such as viruses, worms, Trojans, ransomware, and spyware.
*Network-based:* threats targeting network infrastructure, such as denial-of-service (DoS) attacks, distributed denial-of-service (DDoS) attacks, and man-in-the-middle (MitM) attacks.
*Social engineering:* involves threats that exploit human vulnerabilities and manipulate individuals to gain unauthorised access or disclose sensitive information.
*Physical:* threats that involve physical access to systems or equipment, including theft, vandalism, or tampering.
*Information disclosure:* cover threats that result in the unauthorised disclosure of sensitive information, such as data breaches or unauthorised access to confidential data.
*Software vulnerabilities:* include threats that exploit software weaknesses, such as software bugs, coding errors, or insecure configurations.
*Data integrity:* threats that target the integrity of data, including unauthorised modifications, data corruption, or unauthorised data deletion.

### 5G threat taxonomy unveiled

Amidst the growing diversity of methodologies, taxonomies, and frameworks, it becomes increasingly important to cultivate a shared lexicon that unifies the terminology employed to delineate the threat landscape. A fundamental step towards this is the classification of key components, encompassing assets, vulnerabilities, and threats. Notably, the establishment of a universal taxonomy within the realm of 5G security holds the promise of enhancing communication and collaboration across a spectrum of stakeholders involved in policymaking, regulation, product development, system deployment, and operation.

To facilitate this imperative, ENISA introduces an insightful threat taxonomy, which is put forward in Annex B ^2, p. 124^. This taxonomy serves as a cornerstone for fostering a coherent understanding of 5G security threats, thereby bolstering the resilience and effectiveness of the entire 5G ecosystem.

The following list represents the principal categories of the ENISA Threat Taxonomy:

- Nefarious activity/abuse (NAA): “intended actions that target ICT systems, infrastructure, and networks by means of malicious acts with the aim to either steal, alter, or destroy a specified target”.
- Eavesdropping/Interception/Hijacking (EIH): “actions aiming to listen, interrupt, or seize control of a third party communication without consent”.
- Physical attacks (PA): “actions which aim to destroy, expose, alter, disable, steal or gain unauthorised access to physical assets such as infrastructure, hardware, or interconnection”.
- Damage (DAM): intentional actions aimed at causing “destruction, harm, or injury of property or persons and results in a failure or reduction in usefulness”.
- Unintentional Damage (UD): unintentional actions aimed at causing “destruction, harm, or injury of property or persons and results in a failure or reduction in usefulness”.
- Failures or malfunctions (FM): “Partial or full insufficient functioning of an asset (hardware or software)”.
- Outages (OUT): “unexpected disruptions of service or decrease in quality falling below a required level“.
- Disaster (DIS): “a sudden accident or a natural catastrophe that causes great damage or loss of life”.
- Legal (LEG): “legal actions of third parties (contracting or otherwise), in order to prohibit actions or compensate for loss based on applicable law”.

Table 1 below delineates the principal elements of the front end of this taxonomy, under category Nefarious Activity/ Abuse of assets (NAA) in relation to its sub-categoties ‘potential impact’ and ‘affected assets’. which are described in more depth in table format in Section 6, 5G Threats ^2, pp. 103–106^.

**Table 1.** ENISA taxonomy of 5G threats. Under Threat Type, ‘Nefarious Activity/ Abuse of assets (NAA),’ the individual threats are marked in bold and highlighted in blue. The symbols 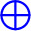 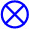 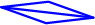 and bracketed numbers [1]–[5] are tied to the elements depicted in Figure 1, as are the individual threats highlighted in blue in the table.

**Manipulation of network** ** configuration/data forging** 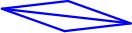 - Routing tables manipulation - CORE configuration data tampering - DNS manipulation - Manipulation of access network and radio technology configuration data - Exploitation of misconfigured or poorly configured systems/networks - Registration of malicious network functions - Security data tampering - Network implementation data tampering - OS services tampering **Potential Impact** - Information integrity - Information destruction - Service unavailability **Affected Assets** - SDN, NFV, MANO, RAN, RAT (system, network, security configuration data), business services	**Exploitation of software, ** **hardware vulnerabilities** **[5]** - Zero-day exploits - Abuse of edge open application programming interfaces (APIs) - Application programming interface (API) exploitation - Software tampering - System execution hijack **Potential Impact** - Information integrity - Information destruction - Service unavailability **Affected Assets** - SDN, NFV, MANO, RAN, RAT, MEC, API - Physical infrastructure - Business applications - Security controls - Cloud, virtualisation - Subscribers’ data - Application data - Security data - Network data - Business services	**Denial of service (DoS)** **[2]** - Distributed denial of service (DDoS) - Flooding of core network components - Flooding of base stations - Amplification attacks - Jamming device radio interface - Jamming base station radio interface - Edge node overload - Authentication traffic spikes **Potential Impact** - Service unavailability - Outage **Affected Assets** - SDN, NFV - RAN, RAT - MEC - CLOUD - Network services - Business services **Remote access exploitation** - intra-RAT mobility mechanism hijack - RAT session hijack (this impacts system integrity, data confidentiality; and affects assets RAT, SDN, NFV, MANO, CLOUD)
**Malicious code/software** **[1]** - Injection attacks (SQL, XSS)[] - Rootkits - Rogueware - Worms/trojan - Botnet - Ransomware - Malicious network functions - Malware attacks on network products - Malware attacks on business applications **Potential Impact** - Service unavailability - Information integrity - Information destruction - Other software asset integrity - Other software asset Destruction **Affected Assets** - Data network - Business applications - Security controls - Cloud, virtualisation - Subscribers’ data - Application data - Security data - Network data - Business services - Network services	**Abuse of remote access to the network** **[4]** - Abuse on external remote services to network products (e.g. VPN) **Potential Impact** - Information & System integrity and confidentiality - Initial unauthorised access - Persistence **Affected Assets** - SDN, NFV, RAN, RAT - Intra-RAT (Subscribers, Application, Security, Network data) **Abuse of information ** **leakage [3]** - Theft and/or leakage from network traffic - Theft and/or leakage of data from cloud computing - Abuse on security data from audit tools - Theft/breach of security keys - Unauthorised access to user plane data and signalling data **Potential Impact** - Information integrity, destruction, confidentiality **Affected Assets** Data storage/ repository Subscribers & monitoring data - Cryptographic keys	**Abuse of authentication** 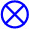 - Authentication traffic spikes - Abuse of user authentication/ authorisation data by third parties’ personnel - Abuse of the application management function (AMF) authentication and key agreement procedure - Abuse the credentials of existing accounts **Potential Impact** - Information integrity - Information destruction - Service unavailability - Initial unauthorised access - Persistence - Security data **Affected Assets** - Network service - Network functions - Subscribers’ data - Application data - Security data - Network data **Lawful interception function abuse** 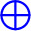 **Potential Impact** - Information integrity - Information destruction **Affected Assets** - Subscribers’ data - User subscription profile data

**Figure 1.**
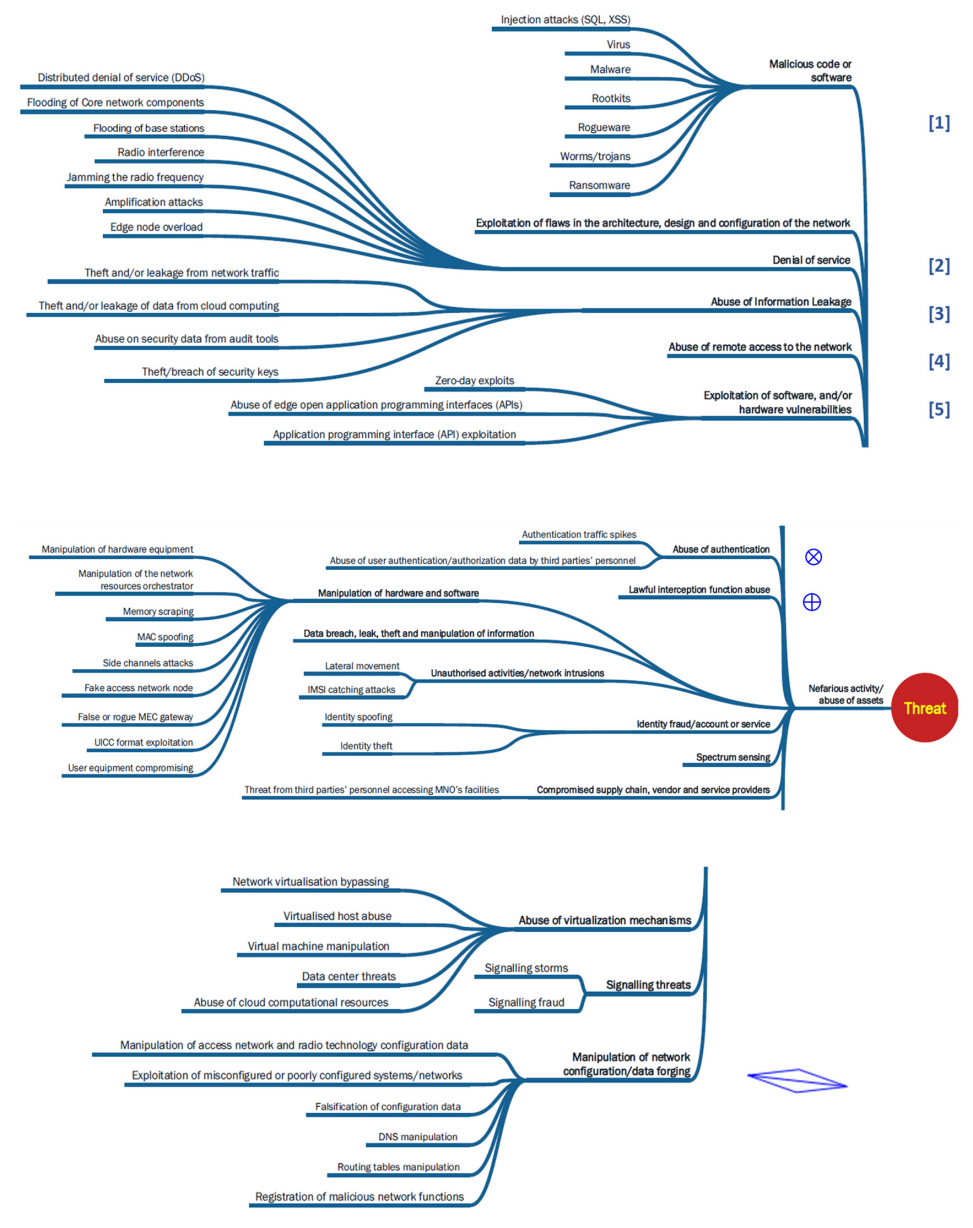
ENISA 5G taxonomy: partial view. The figure depicts the hierarchical structure of part of the ENISA 5G taxonomy, excerpted from the original ^2, p. 128^. The symbols and bracketed numbers map to the threat elements highlighted in blue in Table I above. © European Union Agency for Cybersecurity (ENISA), 2020.

### Hybrid threat taxonomy

A taxonomy of hybrid threats could be classified in terms of i) an analytical framework; ii) various domains; and iii) phases. The European Commission Joint Research Centre (EC JRC) and The European Centre of Excellence for Countering Hybrid Threats (Hybrid CoE) have produced a *Conceptual Model* ^3^ that outlines these categories in significant detail. The high-level overview of this hierarchical model is delineated in the following way:

*Analytical Framework:*

- Actors (and their strategic objectives)
- Tools applied by the actor
- Domains that are targeted
- Phases (including the types of activity observed in each phase)

More specifically, *Domains* are categorised as follows: Cyber, Information, Intelligence, Economy, Infrastructure, Administration, Space, Military/Defense, Legal, Social/Societal, Culture, Diplomacy, Political.

Finally, three key *phases* have been identified in the *Conceptual Model*. These are: priming, destabilisation, coercion; and these are associated with activities that include interference, influence, operations and campaigns, as well as hybrid warfare.

### Ontology: a general overview

An ontology encompasses not only the hierarchical structure but also relationships, attributes, and a formal specification of concepts and their interconnections.

To transform the presented hierarchical model into a complete ontology, we would need to:

*Define Classes*: Clearly define classes (concepts) for each element in the hierarchy.
*Specify Relationships*: Describe relationships between classes using Resource Description Framework (RDF), ^iii^ indicating how different concepts relate to each other.
*Include Attributes:* Define attributes or properties associated with each class. These properties describe characteristics or features of the concepts.
*Use Ontology Languages:* Utilise ontology languages like OWL (Web Ontology Language) ^iv^ to formalise the ontology, specifying classes, relationships, and attributes in a machine-readable format.

Once fully formalised, the ontology can be used by machines to reason, infer, and process information, providing a structured and semantically rich representation of the domain.

### Hybrid threat ontology: *Conceptual Model*

**Figure 2.**
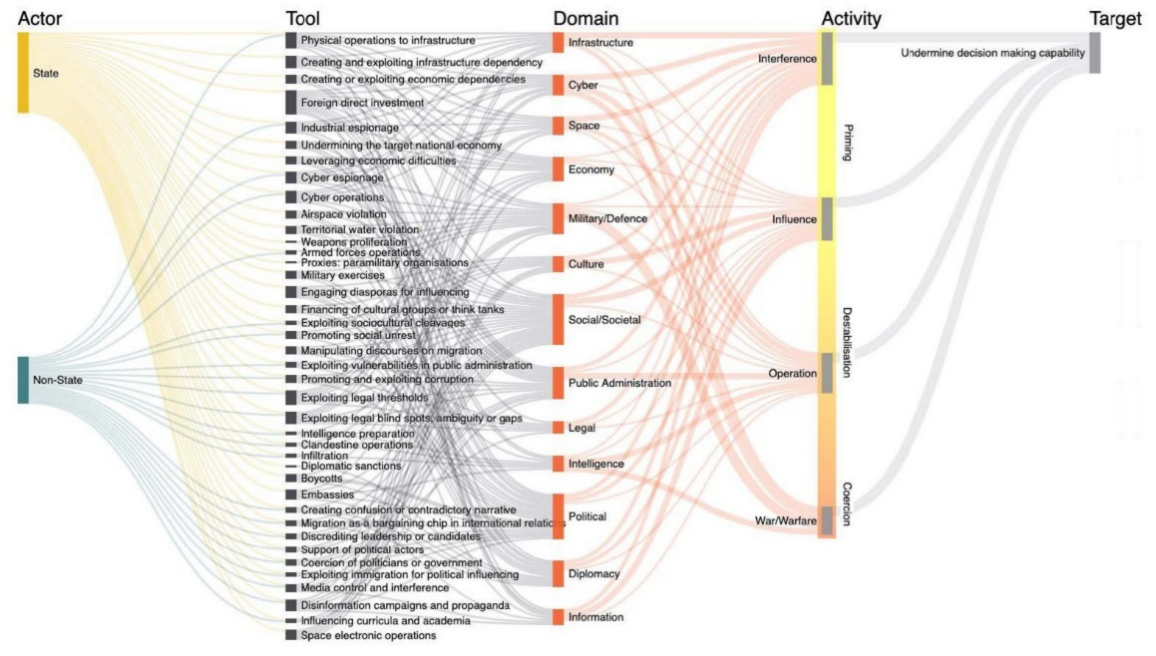
Ontology of Hybrid Threats. An overview of the Hybrid Threats Ontology is depicted in the figure ^3, p. 13^. It showcases actors, tools, targets, and interconnections within hybrid threat domains. Hybrid threats blend conventional and unconventional tactics to achieve strategic goals. State and non-state actors leverage cyber, physical, and information tools to target military, government, infrastructure, economy, and societal sectors. Their objectives encompass disruption, destabilization, coercion, discrediting, influence, information operations, priming, and exploitation. © European Union and Hybrid CoE, 2021.

Determining relationships between ontological categories involves identifying and defining the relationships or connections between different categories within the ontology (refer to Figure 2 below).

In the context of hybrid threats, determining relationships between ontological categories could include:

*Hierarchical Relationships:* Identifying hierarchical relationships between categories, such as broader categories and their subcategories.
*Associative Relationships*: Defining associative relationships between categories that share common attributes or characteristics.
*Dependency Relationships*: Identifying dependencies or dependencies between categories, where one category relies on another.
*Temporal Relationships:* Consider temporal relationships between categories, where certain threats may evolve or emerge based on the progression of time or advancements in technology. This could involve tracking the evolution of specific threat vectors within the hybrid threat landscape.

***Actors***

In the intricate world of hybrid threats, the primary actors come in two distinct forms, each with its unique motives and methods.

*State Actors:* These are the behemoths of the geopolitical stage, nations and governmental agencies with strategic objectives that often shape the course of history. Within this category, we find subcategories such as nation-states and various government agencies, each with its set of complex relationships. These relationships may involve collaboration on shared goals, alliances born of mutual interest, or conflicts that escalate into digital battlegrounds. The attributes of state actors encompass a range of geopolitical goals and policy interests, driving their actions in the hybrid arena.
*Non-State Actors:* In the shadows of the digital realm, non-state actors, especially criminal entities, wield their power. This diverse category includes organised crime groups and hacktivist collectives, united by their pursuit of financial gain or ideological motivations. Collaboration, support, and opposition define their intricate relationships, and their actions are often driven by motives that include financial gain and ideological convictions.

***Tools***

To wage a hybrid threat, actors deploy an arsenal of tools that span the spectrum of modern technology.

*Cyber Tools:* The digital realm serves as the battleground for many hybrid threats, with actors employing a variety of cyber tools. These tools encompass malware attacks, phishing schemes, and data breaches, each with its unique subcategory. The relationships between these tools are often seen in the context of attack vectors and the targeted systems. Attributes such as attack complexity and data compromise define their impact on the hybrid landscape.
*Disinformation Campaigns:* In an era of information overload, disinformation campaigns have become a potent weapon. Actors manipulate narratives and exploit societal divisions through the dissemination of false or misleading information. The relationships between these campaigns and their psychological impact on public opinion are defining features. Attributes include the erosion of trust and social polarisation, both of which play a pivotal role in hybrid strategies.
*Economic Manipulation:* For some actors, hybrid warfare extends to economic battlegrounds. Economic espionage and financial manipulation are their chosen tools, often targeting financial markets and trade networks. The relationships here are seen in the context of financial impact and motive analysis, as actors seek to disrupt economic stability and gain strategic advantages.

***Domains***

The domains targeted by hybrid threats are as diverse as the actors themselves, encompassing critical aspects of modern society. These include:

- *Infrastructure:* Within the infrastructure domain, critical facilities such as energy and transportation systems are prime targets. Relationships are defined by dependencies and vulnerabilities, while attributes focus on the criticality of these systems and the measures in place to ensure their resilience.
- *Cyber:* In the digital age, cyber threats have become a dominant force. Malware attacks, phishing campaigns, and data breaches threaten the very fabric of the digital world. Relationships are seen in attack vectors and targeted systems, with attributes highlighting the complexity of attacks and the compromise of sensitive data.
- *Space:* The space domain extends beyond the Earth's atmosphere, with threats including satellite disruption and communication interference. Relationships are defined by technological reliance and global communication, while attributes emphasise the vulnerability of satellite networks and their impact on GPS.
- *Economy:* Economic manipulation is a subtle yet potent form of hybrid threat. Financial markets and trade networks become the battlegrounds where relationships center on financial impact and motive analysis.
- *Military/Defence:* Hybrid threats extend into the military and defense domain, encompassing disinformation campaigns and hybrid warfare tactics. Relationships emerge in the context of geopolitical conflicts and military preparedness, with attributes reflecting military objectives and deterrence strategies.
- *Culture:* Cultural narratives and identity manipulation are tools used to sow discord within societies. Relationships lead to societal divisions and media influence, while attributes consider cultural sensitivities and the effectiveness of influence campaigns.
- *Social/Societal:* The social and societal domain is shaped by social media manipulation and the manipulation of public sentiment. Relationships are characterised by psychological impact and shifts in public opinion, with attributes focusing on trust erosion and social polarisation.
- *Public Administration:* Government systems disruption and policy manipulation are threats that target public administration. Relationships center on governance stability and policy impact, with attributes highlighting administrative vulnerabilities and policy alignment.
- *Legal:* Legal systems themselves can become targets, leading to legal system manipulation and breaches of international law. Relationships are defined by legal frameworks and international treaties, with attributes considering the erosion of the rule of law and the challenges posed by legal issues.
- *Intelligence:* The intelligence domain involves espionage and information gathering. Relationships revolve around surveillance capabilities and foreign intelligence, with attributes focusing on intelligence objectives and counterintelligence efforts.
- *Diplomacy:* Diplomatic tensions and manipulation of negotiations fall within the domain of diplomacy. Relationships are seen in international relations and diplomatic initiatives, while attributes highlight diplomatic objectives and negotiation tactics.
- *Political:* In the political domain, actors seek to interfere with political processes and destabilise regimes. Relationships are defined by the political landscape and efforts to bring about regime change, with attributes delving into political motivations and electoral influence.
- *Information:* Information is a powerful tool in hybrid warfare, with threats including disinformation, misinformation, and deepfakes. Relationships extend to media platforms and their influence on public perception, while attributes encompass information.

The relationships between these ontological categories are depicted in the *Conceptual Model* and provide a comprehensive and interconnected view of the threat landscape, allowing for a deeper understanding of how different categories relate to each other and how these contribute to a robust ontology for hybrid threats ( Figure 2).

***Phases***

*Priming:* The initial phase of a hybrid threat sets the stage for what follows. This phase is characterised by information campaigns and the careful setting of narratives. Relationships within priming involve the influence exerted by media platforms and their impact on public perception. Attributes defining this phase include the effectiveness of message dissemination and the skillful use of emotional triggers to shape attitudes and opinions.
*Destabilisation through Operations and Campaigns*: As hybrid threats progress, the phase of destabilisation through operations and campaigns emerges. It encompasses a range of tactics, including cyberattacks and disinformation campaigns. Relationships during this phase revolve around the sectors that become targets and the coordination of operational efforts. Attributes come to the forefront, highlighting the sophistication of attack techniques and the scope of these operations as they disrupt and sow chaos.
*Coercion through Hybrid Warfare*: In the final phase, coercion through hybrid warfare takes center stage. This phase may involve economic pressure and psychological tactics, often backed by political objectives and military brinkmanship. Relationships in this critical phase are defined by the pursuit of political goals and the strategic use of military posturing. Attributes play a decisive role, shedding light on the coercive strategies employed and the far-reaching geopolitical impact of hybrid threats in action.

The *Conceptual Model* offers a representation of the Phases component in the context of a hybrid threat ontology. The Hybrid Threat Ontology, with its actors, tools, domains, and phases, offers a comprehensive framework to dissect and understand the complex landscape of hybrid threats. Each element within this ontology contributes to a nuanced perspective, enabling a more informed and strategic response to the ever-evolving challenges posed by hybrid threats.

### The cyber domain

**Figure 3.**
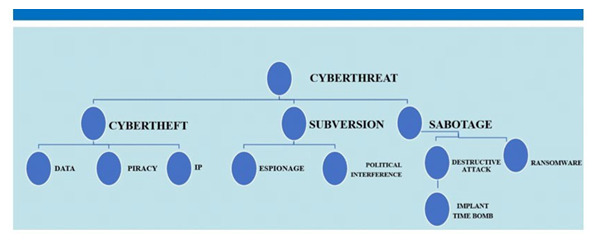
Depicting the complex character of the cyberthreat landscape. This figure illustrates how the landscape extends beyond its own cyber domain, highlighting its multifaceted nature. This landscape is constantly evolving and includes a wide range of threats, actors, and motivations. The figure depicts a variety of cyberthreats, including cybercrime, espionage, sabotage, and destructive attacks. These threats can be carried out by a variety of actors, including state-sponsored actors, criminal organizations, and individuals. The motivations for cyberattacks can also vary widely, from financial gain to political espionage to simply causing disruption. © EU-HYBNET, 2021.

In the context of hybrid threats the cyber domain might be expanded with reference to the categories depicted in Figure 3.

***Vignette 1: Cyberthreat***

Imagine an unsuspecting EU country, let's call it NatioVeritas, a peaceful and prosperous country, with a strong economy and a high standard of living. However, NatioVeritas is also a target of state-sponsored hybrid attacks from a hostile foreign power, let's call it FalsitasGens.

FalsitasGens seeks to destabilise NatioVeritas and undermine its influence within the EU. To achieve this, FalsitasGens launches a coordinated hybrid attack against NatioVeritas, using a variety of cyber and physical methods.

*Cybertheft*

In the early hours of dawn, a group of hackers launched an attack on NatioVeritas's government servers. They skillfully bypassed the security measures, gaining access to sensitive data, including financial records, and Intellectual Property (IP). The stolen data, now in their possession, was swiftly copied and transmitted to remote servers outside NatioVeritas's borders. The breach remained undetected.

*Subversion*

Simultaneously, another group, working on behalf of FalsitasGens, initiated a campaign of digital subversion. They infiltrated social media platforms, blogs, and news sites, spreading false narratives designed to create dissent and mistrust within NatioVeritas's populace. Funding opposition groups and employing tools of espionage, they seised government classified digital documents and amplified their messages, eroding public trust in the government.

*Sabotage*

As the subversion campaign unfolded, a more determined plan was set in motion. Sleeper agents activated by FalsitasGens infiltrated NatioVeritas's power grid. Exploiting vulnerabilities, they disrupted critical infrastructure nodes, leaving major cities without power. In a coordinated move, ransomware attacks targeted the healthcare system, demanding large sums of cryptocurrency as hospitals struggled to regain control of their systems.

• *Implant Time Bomb*

Unbeknownst to NatioVeritas, a digital time bomb had been concealed within the stolen data. It lay dormant, awaiting activation. Crafted by the FalsitasGens, this malicious code posed a significant threat. As tensions rose, the countdown began, and NatioVeritas grappled with simultaneous cybertheft, subversion, and sabotage.

The lines between these hybrid threats blurred, leaving NatioVeritas vulnerable. The nation's leaders raced against time to unravel the complex web of attacks, discover the identity of FalsitasGens, and defuse the digital time bomb.

In summary, NatioVeritas had been slowly crippled through these combined cyberattacks, propaganda efforts, and escalating provocations. The hybrid campaign achieved FalsitasGens's goals of sowing instability in NatioVeritas while maintaining plausible deniability and staying below the threshold of open war. NatioVeritas was left confused and divided, unprepared to defend itself against FalsitasGens aggression through both unconventional and conventional means.

### Hybrid cyberthreat ontology

Considering Figure 3, the taxonomy of threats associated with this Vignette, namely, Cybertheft, Subversion, and Sabotage, along with their respective sub-categories, if grouped together under one category, could be considered as a hybrid cyberthreat.

The ontology in our example could be described in this way:

***Classes:***

HybridCyberthreat: This class represents the overall category of hybrid cyber threats. It includes subcategories (sub-classes) such as Cybertheft, Subversion, and Sabotage.

*Cybertheft*: This sub-class represents a type of hybrid cyber threat related to stealing data, piracy, and intellectual property (IP).
*Subversion*: This sub-class represents a type of hybrid cyber threat involving espionage and political interference.
*Sabotage:* This sub-class represents a type of hybrid cyber threat involving destructive attacks, ransomware, and implanting time bombs.

***Relationships:***

*hasCategory:* This relationship indicates that a hybrid cyber threat falls into a specific category, such as Cybertheft, Subversion, or Sabotage.
*hasSubcategory*: This relationship specifies that a specific category, like Cybertheft, may have subcategories like Data, Piracy, and IP.

***Individuals:***

Each class represents a type of hybrid cyber threat, and individuals under these classes represent specific instances or examples of those threats.

In terms of a class hierarchy our ontology shows how different classes are related. For example, HybridCyberthreat is the parent class, and Cybertheft, Subversion, and Sabotage are its child classes. Cybertheft, in turn, has subcategories like Data, Piracy, and IP.

This descriptive ontology provides a structured way to categorise and understand hybrid cyber threats, including their subcategories and relationships. It can help organise information and knowledge related to these threats for analysis and decision-making in cybersecurity. In this context, our ontology differs from any of those presented in the literature identified by our Google search referred to earlier.

### Resource description framework (RDF)

RDF is a framework for representing knowledge in a structured manner using web resources. It provides a way to describe resources, their properties, and the relationships between them. RDF is commonly used in ontologies to define concepts and their interconnections. It follows a subject-predicate-object format, where resources are represented as nodes connected by directed edges called triples. These triples consist of a subject (resource), a predicate (property or relationship), and an object (value or another resource). This structure allows for the creation of detailed and interconnected knowledge graphs, facilitating data integration, sharing, and reasoning in ontologies.

Table 2 provides code in RDF/OWL syntax, and it's used to define the structure of an ontology related to hybrid cyberthreats. It defines classes, properties, and individuals within an ontology. It represents a basic ontology for hybrid cyberthreats, including classes like "HybridCyberthreat," and sub-classes such as "Cybertheft," "Subversion," and “Sabotage”, along with relationships between them.

**Table 2.** The RDF/OWL representation for the hybrid cyberthreat ontology embodied in Vignette 1.

1. <?xml version=”1.0”?> <!DOCTYPE rdf:RDF [ <!ENTITY xsd “http://www.w3.org/2001/XMLSchema#” > ]> <rdf:RDF xmlns:rdf=”http://www.w3.org/1999/02/22-rdf-syntax-ns#” xmlns:rdfs=”http://www.w3.org/2000/01/rdf-schema#” xmlns:owl=”http://www.w3.org/2002/07/owl#”> <!—Classes → <owl:Class rdf:about=”#HybridCyberthreat”/> <owl:Class rdf:about=”#Cybertheft”/> <owl:Class rdf:about=”#Subversion”/> <owl:Class rdf:about=”#Sabotage”/> <owl:Class rdf:about=”#Data”/> <owl:Class rdf:about=”#Piracy”/> <owl:Class rdf:about=”#IP”/> <owl:Class rdf:about=”#Espionage”/> <owl:Class rdf:about=”#PoliticalInterference”/> <owl:Class rdf:about=”#DestructiveAttack”/> <owl:Class rdf:about=”#Ransomware”/> <owl:Class rdf:about=”#ImplantTimeBomb”/> <!—Relationships → <rdf:Property rdf:about=”#hasCategory”/> <rdf:Property rdf:about=”#hasSubcategory”/> <!—Individuals → <HybridCyberthreat rdf:about=”#HybridCyberthreat”/> <Cybertheft rdf:about=”#Cybertheft”/> <Subversion rdf:about=”#Subversion”/>	2. <Sabotage rdf:about=”#Sabotage”/> <Data rdf:about=”#Data”/> <Piracy rdf:about="#Piracy"/> <IP rdf:about="#IP"/> <Espionage rdf:about="#Espionage"/> <PoliticalInterference rdf:about="#PoliticalInterference"/> <DestructiveAttack rdf:about="#DestructiveAttack"/> <Ransomware rdf:about="#Ransomware"/> <ImplantTimeBomb rdf:about="#ImplantTimeBomb"/> <!-- Class Hierarchy --> <rdf:Description rdf:about="#HybridCyberthreat"> <rdf:type rdf:resource="http://www.w3.org/2002/07/owl#Class"/> <rdf:subClassOf rdf:resource="http://www.w3.org/2002/07/owl#Thing"/> <hasCategory rdf:resource="#Cybertheft"/> <hasCategory rdf:resource="#Subversion"/> <hasCategory rdf:resource="#Sabotage"/> </rdf:Description> <rdf:Description rdf:about="#Cybertheft"> <rdf:type rdf:resource="http://www.w3.org/2002/07/owl#Class"/> <rdf:subClassOf rdf:resource="#HybridCyberthreat"/> <hasSubcategory rdf:resource="#Data"/> <hasSubcategory rdf:resource="#Piracy"/> <hasSubcategory rdf:resource="#IP"/> </rdf:Description> <!-- Additional relationships and individuals can be added as needed --> </rdf:RDF>

RDF/OWL is a standard language for representing ontologies and knowledge graphs in a machine-readable format. It's designed to define classes, properties, and individuals in a way that can be processed by semantic web tools and applications.

### ENISA Threat Landscape 2022

We now integrate the 'ENISA Threat Landscape 2022' report ^4^ into our discussion considering that it is a recent volume in a series of assessments that delve into the cybersecurity threat landscape. The report aims to identify the threats, major trends in those threats, the actors behind them, their attack techniques, as well as an analysis of their impact and motivation. The report has been crafted with assistance from ENISA’s ad hoc Working Group on Cybersecurity Threat Landscapes (CTL).

During the reporting period spanning July 2021 to July 2022 several noteworthy threats have emerged. These include ransomware, malware, social engineering attacks, data breaches, denial of service incidents affecting availability, internet based threats impacting availability disinformation campaigns involving misinformation spreading and supply chain attacks.

For each of these threats the report provides information about attack techniques employed by threat actors; incidents that have occurred; trends observed during this period; and recommended mitigation measures.

One standout trend during this reporting period revolves around how geopolitics has exerted an influence on the cybersecurity threat landscape; notably, so was the Russia Ukraine conflict which resulted in changes such as heightened activity coinciding with military actions and mobilization efforts among hacktivist groups. Furthermore, nation state actors were observed engaging in cyber activities linked to this conflict. Geopolitical dynamics continue to play a role, in shaping cyber operations with destructive attacks increasingly utilized by state sponsored entities.

The report also mentions the increasing trend of hacktivism and the use of disinformation as a tool in cyber warfare even before physical conflicts start. These trends demonstrate the ever-evolving nature of cyber threats. Threat actors adapt their strategies, methods and procedures to exploit emerging vulnerabilities and opportunities.

The ENISA Threat Landscape 2022 report addresses evolving threat vectors, analysis of targets, as well as categories of threat actors.

Important findings from the ENISA Threat Landscape 2022 report, which highlight trends observed during the reporting period are as follows:

- Ransomware and Attacks on Availability; Ransomware attacks and disruptions to availability; in particular, Distributed Denial of Service (DDoS) attacks were most prevalent during the reporting period. The ongoing conflict played a role in the rise of availability attacks.
- Phishing as the Primary Initial Access Method; Phishing continued to be the method for initial access. Phishing attacks have become more sophisticated taking advantage of user fatigue and utilizing targeted tactics based on contexts.
- Resurgence of Malware; After a decline linked to the COVID 19 pandemic incidents involving malware were on the rise again.
- Evolution of Extortion Techniques; Extortion techniques evolved with the use of leak sites where threat actors publish stolen data to exert pressure, on victims.
- Complex DDoS Attacks; DDoS attacks grew in terms of scale and complexity specifically targeting networks and IoT devices. They were increasingly used within the context of cyber warfare.
- Increasing Capabilities of Threat Actors; Resourceful threat actors utilised zero day exploits to achieve their strategic objectives. As organisations strengthened their defenses against cyber threats adversaries encountered obstacles prompting them to develop or obtain unknown vulnerabilities.
- Ransomware Groups Strategies; Ransomware groups frequently retired and rebranded themselves to elude law enforcement and avoid sanctions;
- Hacker, as a Service Business Model; The hacker as a service business model gained momentum. Experienced growth starting in 2021.
- Focus on Supply Chain Attacks; Threat groups exhibited a heightened interest in and improved capability for carrying out supply chain attacks and targeting Managed Services Providers (MSPs).
- Emergence of New, Combination and Emerging Threats; New, combination and emerging threats had an impact on the threat landscape. The Pegasus case received media attention. Prompted governmental responses, leading to similar cases involving surveillance activities and the targeting of civil society.
- Consent Phishing; Consent phishing attacks involve sending users links that grant attackers access, to applications and services when clicked. This tactic is increasingly employed by threat actors.
- Data compromise is increasing year on year; the number of incidents involving compromised data has been steadily increasing over the years. This can be attributed to the growing importance of data analysis and the large amount of data being collected in society.
- Machine Learning (ML) models; Cyberattacks are increasingly targeting machine learning (ML) models, which form the foundation of distributed systems.
- AI-enabled disinformation and deepfakes; One concerning trend is the use of intelligence (AI) to create disinformation and deepfake content. This includes bots that mimic personas and disrupt processes, such, as the 'notice and comment' rulemaking process and community interactions.

What is more, the ENISA report categorises threat actors into four groups.

State Sponsored Actors; These are threat actors backed by nation states.
Cybercrime Actors; Individuals or groups involved in motivated cybercrime.
Hacker for Hire Actors; Those who offer hacking services for a fee.
Hacktivists; Activists who engage in hacking activities for ideological reasons or social causes.

ENISA’s continuous analysis has identified trends, patterns and insights related to each threat discussed in the report. The findings and conclusions are based on available resources mentioned in the document. The report primarily targets decision makers and policymakers while also serving as a resource for the technical cybersecurity community.

It is essential to have an understanding of these changing patterns and the individuals that pose threats in order to develop robust cybersecurity measures and strategies for protection. We can consider the above delineation of cyber threats in taxonomic terms.

However, we can also consider the ontology for the broader set of cyberthreat elements contained in the ENISA Threat Landscape 2022 report structured according to Classes, Relationships, Individuals, and Class Hierarchy. This is accomplished in Table 3.

**Table 3.** The hierarchical structure of threats and attacks contained in ENISA Threat Landscape 2022.

**Classes:** 1. Threat Categories - Hybrid Cyberthreat - Other Cyber Threats 2. Attack Techniques - Phishing - Ransomware - Malware - Social Engineering - DDoS attacks - Supply-chain attacks - Machine Learning (ML) model attacks - AI-enabled disinformation and deepfakes - Espionage - Political Interference - Data compromise - Extortion techniques - Implant Time Bomb - Piracy - Intellectual Property (IP) theft - Disinformation – misinformation	3. Threat Actors - State-sponsored actors - Cybercrime actors - Hacker-for-hire actors - Hacktivists 4. Impact Categories - Data Breach - Financial Loss - Reputation Damage - Service Disruption - Critical Infrastructure Damage - Intellectual Property Theft 5. Motivation Categories - Financial Gain - Political Influence - Ideological Goals - Espionage - Disruption of Services - Nation-State Agendas
**Relationships:** 1. Utilises Attack Technique: Links Threat Categories to Attack Techniques. 2. Perpetrated by: Associates Threat Actors with Threat Categories. 3. Leads to Impact: Connects Attack Techniques to Impact Categories. 4. Driven by Motivation: Relates Threat Actors to Motivation Categories.	
**Individuals:** 1. Example Threats: - ‘Hybrid Cyberthreat’ (Individual representing a general hybrid threat) - ‘Phishing Attack’ (Individual representing a specific instance of a phishing attack) - ‘Ransomware Attack’ (Individual representing a specific instance of a ransomware attack) - (Similar individuals for other attack techniques) 2. Example Threat Actors: - ‘State-sponsored Actor’ (Individual representing a state-sponsored threat actor) - ‘Cybercrime Actor’ (Individual representing a cybercrime actor) - ‘Hacker-for-hire Actor’ (Individual representing a hacker-for-hire actor) - ‘Hacktivist’ (Individual representing a hacktivist) 3. Example Impact Incidents: - ‘Data Breach’ (Individual representing a specific data breach incident) - ‘Financial Loss Incident’ (Individual representing a specific financial loss incident) - (Similar individuals for other impact categories) 4. Example Motivation Instances: - ‘Financial Gain Motivation’ (Individual representing a motivation for financial gain) - ‘Political Influence Motivation’ (Individual representing a motivation for political influence) - (Similar individuals for other motivation categories)	
**Class Hierarchy:** - ‘Threat Categories’ is a superclass for ‘Hybrid Cyberthreat’ and ‘Other Cyber Threats’. - ‘Attack Techniques’ is a superclass for various attack techniques. - ‘Threat Actors’ is a superclass for different types of threat actors. - ‘Impact Categories’ is a superclass for various impact categories. - ‘Motivation Categories’ is a superclass for different motivation categories.	

Let us now expand upon the narrative of Vignette 1 and consider Vignette 2 in the context of the threat elements put forward in the ENISA Threat Landscape 2022 report.

***Vignette 2: Cyberthreat***

The respective roles of NatioVeritas and FalsitasGens remain the same but are enhanced to take into account additional taxonomic elements.

*Cybertheft*

In the hours of dawn a group of hackers targeted NatioVeritas government servers. They skillfully circumvented security measures gaining access to information such as records and intellectual property (IP). Swiftly copying the stolen data they transmitted it to servers located beyond NatioVeritas borders without detection.

During this cybertheft incident NatioVeritas experienced a surge in compromised data—a trend seen worldwide. The increasing reliance on data in society has led to a rise, in both its collection and importance. Unfortunately, this significance comes at a cost: a growing number of data compromises.

*Subversion*

At the time another group acting on behalf of FalsitasGens launched a subversion campaign.

They managed to infiltrate social media platforms, blogs and news websites with the aim of spreading narratives. Their goal was to sow discord and foster distrust among the people of NatioVeritas. By funding opposition groups and employing espionage techniques they were even able to gain access to government digital documents amplifying their messages and further eroding public trust in the government.

Their campaign of subversion wasn't limited to spreading disinformation: it also extended to manipulating the very foundations of digital society. Machine Learning (ML) models, at the core of modern distributed systems had become targets of attacks. AI-enabled disinformation and deepfakes flooded the online landscape, disrupting the notice-and-comment rulemaking process and community interactions.

*Sabotage*

However, their plan did not end there. Sleeper agents activated by FalsitasGens managed to infiltrate NatioVeritas power grid system. Exploiting vulnerabilities in the infrastructure, they carried out actions that left cities without electricity. As part of a coordinated effort ransomware attacks were launched against healthcare systems demanding amounts of cryptocurrency while hospitals struggled to regain control over their compromised systems.

These acts of sabotage went beyond cyberattacks; they included forms of threats that combined novel methodologies with emerging technologies. The ongoing geopolitical tensions contributed to a surge in attacks aimed at disrupting availability, Distributed Denial of Service (DDoS) attacks. Threat actors significantly expanded their capabilities by using undisclosed vulnerabilities, 0-day exploits, as well as adopting a business model known as ‘Hacker, as a Service.’ Additionally, these threat actors became more proficient in carrying out supply chain attacks, which had a significant impact on the threat landscape.

Unbeknownst to NatioVeritas, the stolen data contained a digital time bomb that remained inactive until triggered. This dangerous code, created by the group known as FalsitasGens bombers posed an additional threat. As tensions escalated, the countdown began. NatioVeritas grappled with simultaneous cyber thefts, subversion attempts, and acts of sabotage.

In summary, NatioVeritas experienced a deterioration due to a combination of cyber attacks, propaganda campaigns, escalating provocations and the emergence of threats from the ever-changing cyber landscape. The hybrid campaign successfully achieved FalsitasGens goals of creating instability within NatioVeritas while maintaining deniability and avoiding warfare. NatioVeritas was left confused and divided, unprepared to defend itself against FalsitasGens’s aggression through both unconventional and conventional means.

Consider once more the Hybrid Cyberthreat components in relation to the RDF coded in Table 4:

**Table:** 

*Cybertheft* - Data - Piracy - Intellectual Property (IP)	*Subversion* - Espionage - Political Interference	*Sabotage* - Destructive Attack - Ransomware - Implant Time Bomb

**Table 4.** The associated RDF for the above components.

@prefix rdf: <http://www.w3.org/1999/02/22-rdf-syntax-ns#>. @prefix rdfs: <http://www.w3.org/2000/01/rdf-schema#>. @prefix owl: <http://www.w3.org/2002/07/owl#>. @prefix ex: <http://example.org#>.	
1. ex:HybridCyberthreat a owl:Class; rdfs:subClassOf owl:Thing. ex:Cybertheft a owl:Class; rdfs:subClassOf ex:HybridCyberthreat. ex:Data a owl:Class; rdfs:subClassOf ex:Cybertheft. ex:Piracy a owl:Class; rdfs:subClassOf ex:Cybertheft. ex:IP a owl:Class; rdfs:subClassOf ex:Cybertheft. ex:Subversion a owl:Class; rdfs:subClassOf ex:HybridCyberthreat	2. ex:Espionage a owl:Class; rdfs:subClassOf ex:Subversion. ex:PoliticalInterference a owl:Class; rdfs:subClassOf ex:Subversion. ex:Sabotage a owl:Class; rdfs:subClassOf ex:HybridCyberthreat. ex:DestructiveAttack a owl:Class; rdfs:subClassOf ex:Sabotage. ex:Ransomware a owl:Class; rdfs:subClassOf ex:Sabotage. ex:ImplantTimeBomb a owl:Class; rdfs:subClassOf ex:Sabotage.

For the previous Hybrid Cyberthreat component, we put forward a different version of the RDF formulation from the one included above. In fact, both versions provided are valid RDF representations of the same ontology. The differences observed between them are primarily due to variations in RDF serialisation formats. RDF can be expressed using different syntax formats while conveying the same semantic information.

Version 1 above for Vignette 1 is in RDF/XML syntax, which is one of the standard serialisation formats for RDF data. Version 2 for Vignette 2 is in Turtle syntax, which is another common serialisation format for RDF. As can be seen, it is more concise and human-readable than RDF/XML.

Significantly, the content and structure of the ontology remain consistent between the two versions. The semantic information represented by the ontology remains the same.

Finally, consider the wide-ranging RDF in Table 5 for the broad elements of the Cyberthreat Ontology that incorporates the taxonomic elements contained in the ENISA Threat Landscape 2022 report.

**Table 5.** The RDF for the elements of the cyberthreat ontology of Vignette 2.

1. # Define the namespaces @prefix rdf: <http://www.w3.org/1999/02/22-rdf-syntax-ns#>. @prefix rdfs: <http://www.w3.org/2000/01/rdf-schema#>. @prefix owl: <http://www.w3.org/2002/07/owl#>. @prefix hybridthreat: <http://example.com/hybridthreat#>. @prefix threatcategory: <http://example.com/hybridthreat/threatcategory#>. @prefix attacktechnique: <http://example.com/hybridthreat/attacktechnique#>. @prefix threatactor: <http://example.com/hybridthreat/threatactor#>. @prefix impactcategory: <http://example.com/hybridthreat/impactcategory#>. @prefix motivationcategory: <http://example.com/hybridthreat/motivationcategory#>. @prefix relationship: <http://example.com/hybridthreat/relationship#>. @prefix individuals: <http://example.com/hybridthreat/individuals#>.	
2. # Define the class hierarchy hybridthreat: a owl:Class; rdfs:subClassOf owl:Thing. threatcategory: a owl:Class; rdfs:subClassOf hybridthreat:. attacktechnique: a owl:Class; rdfs:subClassOf hybridthreat:. threatactor: a owl:Class; rdfs:subClassOf hybridthreat:. impactcategory: a owl:Class; rdfs:subClassOf hybridthreat:. motivationcategory: a owl:Class; rdfs:subClassOf hybridthreat:. data: a owl:Class; rdfs:subClassOf threatcategory:. piracy: a owl:Class; rdfs:subClassOf threatcategory:. ip: a owl:Class; rdfs:subClassOf threatcategory:. espionage: a owl:Class; rdfs:subClassOf threatcategory:. politicalinterference: a owl:Class; rdfs:subClassOf threatcategory:.	3. destructiveattack: a owl:Class; rdfs:subClassOf threatcategory:. ransomware: a owl:Class; rdfs:subClassOf threatcategory:. implanttimebomb: a owl:Class; rdfs:subClassOf threatcategory:. phishing: a owl:Class; rdfs:subClassOf attacktechnique:. socialengineering: a owl:Class; rdfs:subClassOf attacktechnique:. ddosattacks: a owl:Class; rdfs:subClassOf attacktechnique:. supplychainattacks: a owl:Class; rdfs:subClassOf attacktechnique:. mlmodelattacks: a owl:Class; rdfs:subClassOf attacktechnique:. aienabledattacks: a owl:Class; rdfs:subClassOf attacktechnique:. state-sponsoredactors: a owl:Class; rdfs:subClassOf threatactor:. cybercrimeactors: a owl:Class; rdfs:subClassOf threatactor:.
4. hackerforhireactors: a owl:Class; rdfs:subClassOf threatactor:. hacktivists: a owl:Class; rdfs:subClassOf threatactor:. databreach: a owl:Class; rdfs:subClassOf impactcategory:. financialloss: a owl:Class; rdfs:subClassOf impactcategory:. reputationdamage: a owl:Class; rdfs:subClassOf impactcategory:. servicedisruption: a owl:Class; rdfs:subClassOf impactcategory:. criticalinfrastructuredamage: a owl:Class; rdfs:subClassOf impactcategory	5. iptheft: a owl:Class; rdfs:subClassOf impactcategory:. financialgain: a owl:Class; rdfs:subClassOf motivationcategory:. politicalinfluence: a owl:Class; rdfs:subClassOf motivationcategory:. ideologicalgoals: a owl:Class; rdfs:subClassOf motivationcategory:. espionagemotivation: a owl:Class; rdfs:subClassOf motivationcategory:. servicesdisruptionmotivation: a owl:Class; rdfs:subClassOf motivationcategory:. nationstateagendas: a owl:Class; rdfs:subClassOf motivationcategory:.
6. # Define relationships hasCategory: a owl:ObjectProperty; rdfs:domain hybridthreat:; rdfs:range threatcategory:, attacktechnique:, threatactor:, impactcategory:, motivationcategory:. hasSubcategory: a owl:ObjectProperty; rdfs:domain threatcategory:, attacktechnique:, threatactor:, impactcategory:, motivationcategory:; rdfs:range data:, piracy:, ip:, espionage:, politicalinterference:, destructiveattack:, ransomware:, implanttimebomb:, phishing:, socialengineering:, ddosattacks:, supplychainattacks:, mlmodelattacks:, aienabledattacks:, state-sponsoredactors:, cybercrimeactors:, hackerforhireactors:, hacktivists:, databreach:, financialloss:, reputationdamage:, servicedisruption:, criticalinfrastructuredamage:, iptheft:, financialgain:, politicalinfluence:, ideologicalgoals:, espionagemotivation:, servicesdisruptionmotivation:, nationstateagendas:.	
7. utilisesAttackTechnique: a owl:ObjectProperty; rdfs:domain threatcategory:; rdfs:range attacktechnique:. perpetratedBy: a owl:ObjectProperty; rdfs:domain attacktechnique:; rdfs:range threatactor:.	8. leadsToImpact: a owl:ObjectProperty; rdfs:domain attacktechnique:; rdfs:range impactcategory:. drivenByMotivation: a owl:ObjectProperty; rdfs:domain threatactor:; rdfs:range motivationcategory:.z
9. # Define individuals individuals:HybridCyberthreat a hybridthreat:; hasCategory threatcategory:. individuals:PhishingAttack a attacktechnique:; hasCategory phishing:. individuals:RansomwareAttack a attacktechnique:; hasCategory ransomware:. individuals:MalwareAttack a attacktechnique:; hasCategory malware:. individuals:SocialEngineeringAttack a attacktechnique:; hasCategory socialengineering:. individuals:DDoSAttack a attacktechnique:; hasCategory ddosattacks:. individuals:SupplyChainAttack a attacktechnique:; hasCategory supplychainattacks:. individuals:MLModelAttack a attacktechnique:; hasCategory mlmodelattacks:. individuals:AIEnabledAttack a attacktechnique:; hasCategory aienabledattacks:. individuals:EspionageActor a threatactor:; hasCategory espionage:. individuals:PoliticalInterferenceActor a threatactor:; hasCategory politicalinterference:. individuals:DataBreachIncident a impactcategory:; hasCategory databreach:.	10. individuals:FinancialLossIncident a individuals:ReputationDamageIncident a impactcategory:; hasCategory reputationdamage:. individuals:ServiceDisruptionIncident a impactcategory:; hasCategory servicedisruption:. individuals:CriticalInfrastructureDamageIncident a impactcategory:; hasCategory criticalinfrastructuredamage:. individuals:IPTheftIncident a impactcategory:; hasCategory iptheft:. individuals:FinancialGainMotivation a motivationcategory:; hasCategory financialgain:. individuals:PoliticalInfluenceMotivation a motivationcategory:; hasCategory politicalinfluence:. individuals:IdeologicalGoalsMotivation a motivationcategory:; hasCategory ideologicalgoals:. individuals:EspionageMotivation a motivationcategory:; hasCategory espionagemotivation:. individuals:ServicesDisruptionMotivation a motivationcategory:; hasCategory servicesdisruptionmotivation:. individuals:NationStateAgendasMotivation a motivationcategory:; hasCategory nationstateagendas:.

### 5G in the context of the cyber domain

In the realm of cybersecurity, 5G infrastructure introduces a heightened level of complexity and novel opportunities for malicious actors. This intricate landscape necessitates a detailed taxonomy of potential cyber threats tailored specifically to 5G networks. The significance of understanding and addressing these threats cannot be overstated, as they pose substantial risks to organisations, economic entities, and political bodies.

The inherent complexity of 5G networks, their increased reliance on virtualisation, densification, and the diverse array of new services and applications they enable all contribute to the evolving threat landscape. Malicious actors, especially those with advanced capabilities, can leverage these aspects to launch sophisticated cyberattacks. Indeed, numerous examples of such possibilities pervade this threat landscape. In 2020, for example, a group of hackers claimed to have exploited a vulnerability in the 5G core network that allowed them to access customer information, billing records, and network architecture diagrams. ^v^ Moreover, the numerous ways in which 5G networks are more vulnerable to cyberattacks than their predecessors ^vi^ have been explored by numerous practitioners and researchers in the field, giving rise to headlines such as ‘Analysis of 5G Network Security Reveals Attack Possibilities’ ^vii^ or ‘One of 5G’s Biggest Features Is a Security Minefield’ pointing to research undertaken at the Technical University of Berlin. ^viii^

### Ontology of 5G networks

Extending the Hybrid Cyberthreat class to encompass taxonomic categories related to 5G infrastructure architecture is a feasible approach. In our ontology development process, we have previously identified subcategories and relationships to effectively represent the connections between various concepts. Specifically, we've introduced new subcategories within the Hybrid Cyberthreat class to articulate threats associated with 5G infrastructure architecture.

Table 6 below presents the 5G ontology as a hierarchical taxonomy, with the top-level category being 5G Cyberthreats. Major branches include threats to network infrastructure, the core, edge computing, specific attack types, endpoints, physical infrastructure, information, IoT devices, subscribers, and supply chain. Subcategories provide further levels of detail.

**Table 6.** 5G cyberthreats.

1. • Network Infrastructure Threats ⚬ Signaling Attacks ⚬ Physical Layer Attacks ⚬ Link Layer Attacks • Core Network Threats ⚬ Attacks on 5G Core ■ Compromising Core Network Elements ■ Abusing Virtualisation and Slicing • Edge Computing Threats ⚬ Attacks on Edge Computing ■ Hacking Edge Servers ■ Attacking MEC Infrastructure • Specific Attack Types ⚬ Denial of Service ⚬ Man-in-the-Middle Attacks ⚬ Eavesdropping	2. • Endpoint Threats ⚬ Malware on User Devices • Physical Threats ⚬ Attacks on Cell Towers • Information Threats ⚬ Disinformation Campaigns • IoT Threats ⚬ IoT/IIoT Attacks ■ Forming Botnets ■ Manipulating IoT Data • Subscriber Threats ⚬ Subscriber Attacks ■ SIM Swapping ■ Location Tracking • Supply Chain Threats ⚬ Inserting Backdoors

A more elaborate ontology of our sample 5G threat landscape utilizing the power of RDF can be considered in Table 7 below.

**Table 7.** Sample 5G threat landscape utilizing the power of RDF.

[1] @prefix : <http://example.org/5g-threats#> @prefix rdfs: <http://www.w3.org/2000/01/rdf-schema#> :Threat a owl:Class . :NetworkInfrastructureThreat a :Threat; rdfs:subClassOf :Threat . :SignalingAttack a :NetworkInfrastructureThreat; rdfs:subClassOf :NetworkInfrastructureThreat . :PhysicalLayerAttack a :NetworkInfrastructureThreat; rdfs:subClassOf :NetworkInfrastructureThreat . :LinkLayerAttack a :NetworkInfrastructureThreat; rdfs:subClassOf :NetworkInfrastructureThreat . :CoreNetworkThreat a :Threat; rdfs:subClassOf :Threat . :AttackOn5GCore a :CoreNetworkThreat; rdfs:subClassOf :CoreNetworkThreat . :CompromisingCoreNetworkElement a :AttackOn5GCore;	[2] rdfs:subClassOf :AttackOn5GCore . :AbusingVirtualisationAndSlicing a :AttackOn5GCore; :EdgeComputingThreat a :Threat; rdfs:subClassOf :Threat . : AttackOnEdgeComputing a :EdgeComputingThreat; rdfs:subClassOf :EdgeComputingThreat . :HackingEdgeServers a :AttackOnEdgeComputing; rdfs:subClassOf :AttackOnEdgeComputing . :AttackingMECInfrastructure a :AttackOnEdgeComputing; rdfs:subClassOf :AttackOnEdgeComputing . # Additional detailed subclasses and properties :DenialOfService a :Threat; rdfs:subClassOf :Threat . :MITMAttack a :Threat; rdfs:subClassOf :Threat . :Eavesdropping a :Threat; rdfs:subClassOf :Threat . :Malware a :Threat; rdfs:subClassOf :Threat .	[3] :PhysicalAttack a :Threat; rdfs:subClassOf :Threat . :Disinformation a :Threat; rdfs:subClassOf :Threat . :IoTAttack a :Threat; rdfs:subClassOf :Threat . :FormingBotnets a :IoTAttack; rdfs:subClassOf :IoTAttack . :ManipulatingIoTData a :IoTAttack; rdfs:subClassOf :IoTAttack . :SubscriberAttack a :Threat; rdfs:subClassOf :Threat . :SIMSwapping a :SubscriberAttack; rdfs:subClassOf :SubscriberAttack . :LocationTracking a :SubscriberAttack; rdfs:subClassOf :SubscriberAttack . :SupplyChainThreat a :Threat; rdfs:subClassOf :Threat . :InsertingBackdoors a :SupplyChainThreat; rdfs:subClassOf :SupplyChainThreat .

If we return to Vignettes 1 and 2 and consider incorporating cyber threats into a 5G network, our Hybrid Cyberthreat scenario could look like this:

**Table:** 

*Cybertheft* - Data - Piracy - IP	*Subversion* - Espionage - Political Interference	*Sabotage* - Destructive Attack - Ransomware - Implant Time Bomb	*5G Infrastructure Threats* - Network Architecture Vulnerabilities - Piracy Virtualisation Exploits - Densification Risks - New Services Vulnerabilities

Each of these subcategories can represent different aspects of hybrid cyber threats, including those related to the architecture of 5G infrastructure. This allows us to organise and categorise threats in a structured manner, making it easier to analyze and address security concerns related to 5G technology.

The 5G infrastructure threats elements are susceptible to various vulnerabilities, exploits and inherent risks. The key categories in question are defined as follows:

*5G network architecture* **:** This is notably more intricate than previous cellular networks. It encompasses a greater number of components and interfaces, which, while enabling advanced functionalities, simultaneously opens up new avenues for potential cyber attackers to exploit.
*5G virtualisation:* One significant feature of 5G networks is their increasing virtualisation. This means that network functions are implemented using software rather than traditional hardware. While virtualisation offers benefits in terms of flexibility and scalability, it also introduces new cybersecurity challenges, as software-based elements can be more susceptible to cyber threats.
*5G densification:* Compared to earlier cellular networks, 5G networks are significantly denser. This density translates to more cells per square kilometer, enhancing network capacity and performance. However, this densification also increases the physical attack surface of 5G infrastructure, potentially making it more vulnerable to physical security breaches.
*5G network services and applications **:*** 5G technology unlocks a wide array of innovative services and applications, including self-driving cars and smart city solutions. While these advancements promise transformative benefits, they also bring with them new security considerations. The introduction of novel services and applications may expose previously unseen security vulnerabilities that need to be addressed to ensure the safety and integrity of 5G networks.

                                                                                                                                             ***

Here's an expanded scenario of Vignettes 1 and 2 that incorporates additional 5G risks, threats, exploits and vulnerabilities. Vignette 3 includes the following enhancements to Vignettes 1 and 2, in fact, a subset of those attacks listed above in segment Taxonomy of 5G Networks,

***Vignette 3: 5G onslaught***

NatioVeritas had barely recovered from the initial hybrid attacks when an even more insidious threat emerged. FalsitasGens, the state-adversary orchestrating the campaign, recognised that NatioVeritas's 5G infrastructure was a far-reaching element of its national security and economic competitiveness. The stage was set for a new phase of hybrid warfare, one that exploited the vulnerabilities of cutting-edge 5G technology as follows.

*Signaling Attacks*
  FalsitasGens launched signaling attacks against NatioVeritas's 5G network. By manipulating and exploiting vulnerabilities in the 5G signaling protocols, they were able to track the movements of users, deny service to critical applications, and intercept sensitive data. This posed a grave threat to the nation's privacy and security.
*Physical Layer Attacks*
  In a bid to disrupt connectivity and inject malicious traffic into NatioVeritas's 5G network, FalsitasGens deployed physical layer attacks. Jamming and spoofing radio signals disrupted critical communication channels, rendering many services inoperable.
*Link Layer Attacks*
  The link layer, responsible for maintaining the quality of service, became another battleground. FalsitasGens targeted this layer to degrade service quality and execute man-in-the-middle attacks, intercepting data as it flowed between users and critical infrastructure.
*Attacks on 5G Core*
  Recognizing the importance of the 5G core network, FalsitasGens devised a multifaceted attack. They compromised core network elements, including the Access and Mobility Management Function (AMF), Session Management Function (SMF), and User Plane Function (UPF), using a combination of exploits and misconfigurations.
*Attacks on Edge Computing*
  Edge computing, a key feature of NatioVeritas's 5G infrastructure, became a focal point for attack. FalsitasGens hacked into edge servers and Multi-Access Edge Computing (MEC) infrastructure to access sensitive data and facilitate lateral movement across the network.
*IoT/IIoT Attacks*
  Exploiting the vulnerabilities in 5G-enabled IoT and Industrial IoT (IIoT) devices, FalsitasGens formed massive botnets. They manipulated data from compromised IoT devices to cause physical effects, disrupting critical systems.
*Subscriber Attacks*
  NatioVeritas's 5G subscribers became targets of sophisticated attacks. FalsitasGens executed SIM swapping attacks and compromised subscriber credentials for surveillance and fraud. They also leveraged 5G's advanced capabilities like beamforming for location tracking.
*Supply Chain Attacks on a 5G Vendor*
  Recognizing the importance of supply chain security, FalsitasGens inserted backdoors into 5G network gear and software during manufacturing and distribution. This allowed them to maintain persistent access to critical network components.

The expanded bandwidth, virtualisation, and connectivity of 5G created new potential attack surfaces that FalsitasGens exploited as part of their broader hybrid campaign. NatioVeritas found itself under siege on multiple fronts, with critical infrastructure, data privacy, and national security all hanging in the balance. FalsitasGens had raised the stakes in the hybrid war, and NatioVeritas faced its most formidable challenge yet.

### Europol’s review of cyber intelligence

In addition to Vignettes 1, 2 and 3, we present Europol's analysis entitled ‘Sharing of knowledge, know-how and updates to enhance the fight against cybercrime.’ ^ix^

In particular, Europol's comprehensive analysis of ‘The Common Taxonomy for the National Network of CSIRTs’ ^x^ delineates a standardised terminology for classifying cyber incidents, attacks, and occurrences. It offers both a technical perspective and an elevated legal categorisation to facilitate the synchronisation of incidents within the global network of CSIRTs, encompassing national law enforcement bodies, Europol, and Interpol.

To foster enhanced collaboration on a global scale and among all relevant stakeholders involved in combating cybercrime, this Framework connects any cyber event, such as a malware infection, with specific articles within two international legal instruments: the Convention on Cybercrime (ETS 185) ^xi^ and the Directive on Attacks against Information Systems (Directive 2013/40/EU) ^xii^. This approach encourages a unified strategy for addressing cybercrime immediately after an incident and throughout subsequent investigation and prosecution efforts.

Moreover, Europol’s ‘Internet Organised Crime Threat Assessment (IOCTA)’ annual report ^xiii^ stands as Europol's flagship strategic publication, delivering a law enforcement-focused evaluation of evolving threats and significant advancements in the realm of cybercrime.

The IOCTA report provides insights into various cybercriminal activities, encompassing financial fraud, ransomware attacks, online child sexual exploitation, darknet marketplaces, and emerging threats. It sheds light on trends, methodologies, and the consequences of cybercriminal actions across diverse sectors and geographic regions. For a comparison of the Common Taxonomy and the IOCTA scheme, consider Table 8 below. This is a high-level comparative overview of the categories outlined in ‘The Common Taxonomy for the National Network of CSIRTs’ and those featured in the ‘Internet Organised Crime Threat Assessment (IOCTA).’

**Table 8.** A comparison of the Common Taxonomy and the IOCTA scheme.

Common Taxonomy for Law Enforcement and The National Network of CSIRTs		Internet Organised Crime Threat Assessment (IOCTA)
Class of Incidents	Type of Incident	IOCTA’s Position
**Malware/**	Infection Distribution Command & Control (C&C) Malicious connection	Mobile malware has become a scalable business model by introducing overlay attacks, two-factor authentication disruption and SMSspamming capabilities
**Availability/**	Denial of Service (DoS)/ Distributed Denial of Service (DDoS) Sabotage	DDoS for ransom seems to be making a return as criminals use the names of well-known advanced persistent threat (APT) groups to scare their targets into complying with ransom demands.
**Information** ** Gathering/**	Scanning Sniffing **Phishing**	Criminals use delivery services as **phishing** lures. Posing as delivery services, criminals contact potential victims with links to phishing websites pretending to offer information about a parcel delivery, with the aim of obtaining user credentials and payment card details.
**Intrusion ** **Attempt/** **Intrusion/**	Exploitation of vulnerability attempt Login attempt (Successful) Exploitation of vulnerability Compromising an account	Furthermore, IT-infrastructures are extremely intertwined, so a successful intrusion does not only put one company’s clients at risk, but potentially also opens doors to compromise other service providers, giving the attack even greater scalability. Numerous large-scale intrusions like those of Microsoft Exchange Server, SolarWinds and Kaseya coming to light in the past 12 months.
**Information** ** Security/**	Unauthorised access Unauthorised modification/deletion	Users rely on increasingly sophisticated operational security, migrating quickly to other (userless) markets or markets enforcing manual Pretty Good Privacy (PGP) after takedowns.
**Fraud/**	Misuse or unauthorised use of resources False representation	Phishing and social engineering remain the main vectors for payment fraud, increasing in both volume and sophistication. Investment fraud is thriving as citisens incur devastating losses, but business email compromise (BEC) and CEO fraud also remain key threats.
**Abusive** ** Content/**	SPAM Copyright Child Sexual Exploitation, racism or incitement to violence	Overall activity related to child sexual abuse material (CSAM) distribution on P2P networks has increased considerably. The Dark Web remains an important platform for the exchange of CSAM.
**Other/**	Undetermined incident Unclassified incident * Dark Web users are increasingly using Wickr and Telegram as communication channels or to bypass market fees. * Dark Web users are increasingly adopting anonymous cryptocurrencies, such as Monero, and swapping services. * Grey infrastructure is increasingly helping Dark Web users thrive.	*Ransomware affiliate programs are using supply- chain attacks to compromise the networks of large corporations and public institutions and utilise new multi-layered extortion methods. *Ransomware operations are becoming increasingly focused on high-value attacks on large organisations and their supply chains while social engineers are shifting their attention towards upper-level management.

In summary, Europol's Review of Cyber Intelligence offers a comprehensive overview of the current cybercrime landscape, highlighting the evolving threats and the need for international cooperation to combat them effectively. The analysis highlights the importance of sharing knowledge and know-how to enhance the fight against cybercrime. Europol's Common Taxonomy for the National Network of CSIRTs provides a standardized terminology for classifying cyber incidents, attacks, and occurrences, while the Internet Organised Crime Threat Assessment (IOCTA) offers a law enforcement-focused evaluation of evolving threats and significant advancements in the realm of cybercrime. Europol's work in these areas is essential for the global fight against cybercrime in the context of countering hybrid threat onslaughts.

## Part 2: 5G Action Plan for Europe

### Introduction

In Part 1, we delved into the intricate world of modeling the 5G cyberthreat landscape. We explored the realms of taxonomies and ontologies, dissecting ENISA's comprehensive threat taxonomy and venturing into the realms of ontology development. Our journey took us through the meticulous categorization of hybrid threats and the creation of high-level taxonomies for 5G-specific cyberthreats, all meticulously represented using RDF/OWL syntax. Vignettes illustrated hypothetical scenarios, and we incorporated the latest findings from the ENISA Threat Landscape report while considering Europol's cyberthreat analysis.

Now, in Part 2, our focus takes a strategic turn. We explore the integration of the 5G vision with the TEN-T initiative, a cornerstone of European connectivity. Here, we embark on a comprehensive review of the Connecting Europe Facility (CEF) work plan, dissecting its essential components, such as Multi-Access Edge Computing (MEC), Network Function Virtualization (NFV), Software-Defined Networking (SDN), Fog and EDGE and CLOUD computing. As we move forward, we'll unravel how these technological advancements pave the way for a resilient and robust 5G infrastructure across Europe.

### Europe's 5G initiative: progress and future plans

In early 2023, the European Commission unveiled the second phase of its ambitious 5G Action Plan for Europe, ^xiv^ marking a significant stride towards the plan's full realization. A third phase is slated for the end of the same year. This second phase brings into focus a meticulous evaluation of the Connecting Europe Facility (CEF) and the Digital sector's multiannual work programme spanning the years 2021-2025. It zeroes in on key aspects such as extending 5G coverage along planned transport corridors, fostering 5G deployment in Smart Communities, and seamlessly integrating 5G with edge computing and federated cloud facilities.

The overarching aim of the 5G Action Plan for Europe (5GAP) remains unaltered: achieving uninterrupted 5G coverage along the primary transportation routes that crisscross Europe by the year 2025. To attain this goal, the CEF Digital sector has committed to co-funding a series of projects dedicated to the deployment of 5G corridors. The objective here is to stimulate private investment, thereby establishing a comprehensive pan-European transport network interwoven with 5G corridors before the conclusion of the CEF program.

Specifically, the CEF outlines that these corridors must span at least one national border. The length of these corridors can vary, contingent on national circumstances. Factors such as the mode of transportation, geographical conditions, and the funding allocated in the call dictate the extent of deployment or study on each side of the border. Notably, Member States boasting extensive highway and rail networks, encompassing around 1000 kilometers or more, may allocate up to 15% of the corresponding comprehensive corridors within their territory to cross-border segments of 5G corridors.

As we delve into the present context, it becomes imperative to provide a comprehensive overview of the Trans-European Transport Network (TEN-T). ^xv^

### The Trans-European Transport Network (TEN-T)

TEN-T is a policy implemented by the European Union (EU) to support the development and construction of infrastructure networks throughout Europe. The TEN-T policy consists of guidelines that aim to implement an efficient, high-quality network of roads, railways, airports, seaports, inland waterways, and other transportation systems across the EU.

The TEN-T policy identifies nine major transport corridors, with each one involving multiple modes of transport (rail, air, sea, etc.), multiple countries, and encompassing both existing and planned infrastructure. The nine corridors are:

1. Baltic-Adriatic
2. North Sea-Baltic
3. Mediterranean
4. Orient/East-Med
5. Scandinavian-Mediterranean
6. Rhine-Alpine
7. Atlantic
8. North Sea-Mediterranean
9. Rhine-Danube

The aim is to close gaps, remove bottlenecks and technical barriers, and to strengthen social, economic and territorial cohesion in the EU. The strategy behind TEN-T is to create a more efficient and sustainable transportation network that can stimulate economic growth and competitiveness in Europe.

### Revolutionizing transport: merging 5G, NFV, SDN, MEC, Cloud, Fog/Edge computing for TEN-T

The European Union's Trans-European Transport Network (TEN-T) corridors represent a critical infrastructure for seamless mobility across the continent. To fully realize the potential of intelligent transportation systems (ITS) and autonomous vehicles, a robust and future-proof communication infrastructure is essential. The convergence of 5G, Network Functions Virtualization (NFV), Software-Defined Networking (SDN), Multi-Access Edge Computing (MEC), cloud computing, and fog/edge computing technologies offers a compelling solution for achieving this vision ^5– 10^.

### 5G network infrastructure: enabling ubiquitous connectivity

The foundation of this architecture lies in the deployment of a high-performance 5G network infrastructure along the TEN-T corridors. This entails:

- Base stations and RAN: Strategically placed 5G base stations (gNBs) to provide wide area coverage, including rural and underserved areas, ensuring ubiquitous connectivity for all transportation modes.
- Core network: implementation of a 5G core network (5GC) with Network Functions (NFs) handling both control and user plane functions.
- Network slicing: Dynamic network slicing to create virtual networks tailored for specific services and applications, ensuring efficient resource allocation and prioritized bandwidth for critical applications. ^xvi^
- Low latency and high bandwidth: Leveraging 5G's ultra-low latency and high bandwidth capabilities to support real-time applications like autonomous vehicles, augmented reality, and high-definition video streaming.

*NFV for network functions agility*

The adoption of NFV plays an essential role in enabling network agility and flexibility:

- Virtual Network Functions (VNFs): Implementation of VNFs for various network functions such as Evolved Packet Core (EPC), IP Multimedia Subsystem (IMS), and Deep Packet Inspection (DPI). These VNFs can run on NFV Infrastructure (NFVI) components.
- NFVI: Creation of a flexible NFVI comprising servers, storage, and networking equipment that supports VNF deployment. NFVI ensures resource virtualization, efficient management, and rapid scaling.
- NFV MANO: Employ NFV Management and Orchestration (NFV MANO) for VNF lifecycle management, orchestration, and scaling. This includes VNFM, VIM, and NFVO components.

*SDN integration for dynamic network control*

SDN integration provides centralized network control and optimization:

- SDN controller: Incorporation of an SDN controller to manage network traffic, separate control and data planes, and optimize network routing for real-time traffic demands.
- OpenFlow protocol: Utilization of OpenFlow or similar protocols to enable SDN to control and direct traffic efficiently, ensuring dynamic resource allocation and network adaptation.
- Network abstraction: Abstracting physical network resources like switches and routers, allowing for flexible configuration and reallocation of network resources based on traffic patterns and application requirements.

*MEC for low latency at the edge*

MEC nodes deployed along the corridors bring computation and storage resources closer to end-users: ^xvii^

- MEC nodes: Strategic deployment of MEC nodes at strategic points along the corridors, providing computation and storage resources closer to end-users for latency-sensitive applications.
- Local data processing: Enabling local data processing at MEC nodes to reduce latency for applications such as connected vehicles, real-time video analytics, and smart infrastructure.
- MEC orchestration: Integrating MEC orchestration into NFV MANO for seamless coordination between edge and core resources, ensuring optimal utilization and efficient resource allocation.

*Fog/Edge computing: enhancing edge intelligence*

Fog/edge computing extends the capabilities of MEC by enabling intelligent processing and decision-making capabilities at the edge of the network, further reducing latency and improving resource utilization:

- Fog/Edge nodes: Deployment of fog/edge nodes at distributed locations along the corridors, providing computational resources closer to data sources and IoT devices.
- Edge intelligence: Implementation of edge intelligence algorithms on fog/edge nodes to analyze data in real-time, enabling localized decision-making and autonomous operation.
- Fog/Edge orchestration: Integrating fog/edge orchestration with NFV MANO and SDN controllers to optimize the distribution of tasks and resources across the network, ensuring efficient utilization of both cloud, edge, and fog resources.

***Security and orchestration: ensuring network protection and optimization***

Security and orchestration are paramount for a reliable and secure network:

- Security Functions: Implementation of security VNFs and integrated security mechanisms at all levels of the network, including cloud, edge, and fog, to protect against cyber threats, ensuring data integrity, confidentiality, and availability.
- Orchestration: Ensuring coordinated orchestration across cloud, edge, and fog resources to optimize network performance and security dynamically.

*Application layer:*

- Customized network functions: Enable developers to create custom network functions and applications using open APIs provided by the Communication Interface within the application layer.

*Compliance with TEN-T guidelines:*

- Cross-border deployment: Design the architecture to support cross-border segments of 5G corridors in line with TEN-T guidelines.
- Scalability: Ensure the architecture's scalability to accommodate varying corridor lengths and transportation modes.

Merging 5G, NFV, SDN, MEC, Cloud, and Fog/Edge Computing for TEN-T Corridors enables efficient and flexible deployment of 5G infrastructure along transport corridors. It aligns with Europe's 5G Action Plan for Europe, aiming to provide uninterrupted 5G coverage to enhance transportation, economic growth, and competitiveness in Europe by 2025.

### Cloud-Powered scalability: enhancing 5G infrastructure along TEN-T corridors

Centralised cloud computing ^11, 12^ can play a significant role in supporting the implementation of the TEN-T transport corridors plan, particularly when integrated with the 5G, NFV, SDN, MEC and EDGE technologies. Due to its importance, we extend the discussion from above and elaborate here on how traditional cloud computing might be integrated into the TEN-T implementation plan:

*Infrastructure as a Service (IaaS):* Cloud providers can offer IaaS solutions, which include virtualised computing resources such as servers, storage, and networking. These resources can supplement the NFV infrastructure, providing additional scalability and flexibility. Operators can leverage cloud-based IaaS for their NFV infrastructure components, reducing the need for significant on-premises hardware investments.
*Edge Cloud Services:* Cloud providers can extend their services to the edge by deploying cloud nodes at strategic points along the transport corridors. These edge cloud services, often referred to as ‘edge cloud’ or ‘edge computing,’ can provide computation and storage resources closer to end-users and IoT devices, reducing latency and improving the performance of real-time applications.
*Network Function Virtualization (NFV) in the Cloud:* NFV components, including Virtual Network Functions (VNFs) and the NFV Infrastructure (NFVI), can be hosted in the cloud. Cloud-based NFV allows for more efficient resource management, rapid scalability, and the ability to allocate resources on-demand. This can enhance the flexibility and agility of the 5G infrastructure.
*Scalability and Elasticity:* Cloud computing offers inherent scalability and elasticity. Operators can dynamically scale their infrastructure up or down based on traffic demand. During peak periods or when expanding corridor coverage, additional cloud resources can be provisioned and allocated as needed.
*Management and Orchestration:* Cloud-based management and orchestration tools can enhance the automation and optimization of 5G, NFV, and SDN resources. Cloud-native orchestration platforms can efficiently manage the deployment and scaling of network functions and services across the entire infrastructure.
*Data Analytics and Insights:* Cloud platforms provide powerful data analytics and machine learning capabilities. Operators can utilize these capabilities to gain insights from the vast amount of data generated by the 5G infrastructure. This data can be used for traffic optimization, predictive maintenance, and improving the overall efficiency of the transport corridors.
*Security Services:* Cloud providers often offer robust security services, including DDoS protection, threat detection, and encryption. These services can enhance the security of the entire 5G infrastructure, safeguarding against cyber threats and ensuring data privacy.
*Federated Cloud:* Collaboration between multiple cloud providers can establish a federated cloud model, ensuring redundancy and high availability. This approach can further enhance the reliability and resilience of critical services along the transport corridors.

By integrating cloud computing into the TEN-T implementation plan, operators can leverage cloud-based resources, scalability, and advanced services to create a more agile, cost-effective, and high-performance 5G infrastructure. This integration can help achieve the goals of the 5G Action Plan for Europe, ensuring uninterrupted coverage and enhancing transportation, economic growth, and competitiveness by 2025.

                                                                                                                                             ***

### ENISA taxonomy of 5G threats

Consider once more the comprehensive Taxonomy of 5G threats contained in the 'ENISA Threat Landscape 2020' report ^2^ that is delineated in Table 1. Now, if we try to identify the 5G threats that are most likely to thwart the progress of the TENT-T 5G implementation plan across the whole of Europe, we find that most of the individual threats listed in the table and highlighted in blue text could be candidates for execution of malicious acts perpetrated by nefarious adversaries. Recall that these candidates are listed under the following major headings: Manipulation of network configuration/data forging; Malicious code/software; Exploitation of software, hardware vulnerabilities; Abuse of remote access to the network; Abuse of information leakage; Denial of service (DoS); Abuse of authentication; and Lawful interception function abuse.

Furthermore, for each of the threats highlighted in blue text in Table 1 the ‘Affected Assets’ are listed: SDN, NFV, MANO, RAN, RAT, MEC, and Cloud technologies. In the present context, these make up critical aspects of our TEN-T 5G implementation plan. It is essential to recognize that these 5G technologies can be susceptible to threats, and preserving their security needs to be a high priority.

The importance of implementing robust security measures in 5G technologies cannot be underestimated; not doing so can lead to severe privacy and security breaches, economic losses, and disruptions in critical communications infrastructure and services. For example, the lack of adequate security measures can lead to i) increased vulnerability to various attacks, affecting the privacy and security of the users and the system as a whole; ii) exploitation of vulnerabilities in the physical layer involving eavesdropping and data breaches, significantly compromising the confidentiality and integrity of the data transmitted over the network; iii) bandwidth spoofing attacks and intrusions on relays, small cell access points, and base stations; and iv) problematic functional and economic impacts that may disrupt the complex dynamics of the communications ecosystem and impede the performance of critical infrastructure and services.

Let us now produce for the first time in the literature a preliminary ontology in descriptive language according to the required categories. This ontology includes the identified threats and technologies of Table 1 within the context of the TEN-T initiative implementation. Below we define classes, specify relationships, and include attributes for a comprehensive ontology.

*Classes:*

1. ThreatCategory:

   - Description: Represents the general category of threats.

   - Attributes: To Be Determined (TBD).

2. SpecificThreat:

   - Description: Represents specific threats within each threat category.

   - Attributes: TBD.

     - Description: A detailed description of the specific threat.

3. Technology: TBD.

   - Description: Represents the key technologies used in the TEN-T initiative.

   - Attributes: TBD.

*Relationships:*

1. BelongsToCategory:

   - Description: Indicates that a specific threat belongs to a particular threat category.

   - Attributes: TBD.

2. TargetsTechnology:

   - Description: Indicates that a specific threat targets a particular technology.

   - Attributes: TBD.

*Attributes:*

1. Name:

   - Description: The name of a threat category, specific threat, or technology.

   - Data Type: Text.

Now, let's map the identified threats and technologies of Table 1 into this ontology:

*Threat Categories:*

- ThreatCategory 1 (Manipulation of network configuration/data forging')

- ThreatCategory 2 (Exploitation of network vulnerabilities)

- ThreatCategory 3 (Denial of Service)

- ThreatCategory 4 (Remote access exploitation)

- ThreatCategory 5 (Malicious code/software)

- ThreatCategory 6 (Abuse of remote access)

- ThreatCategory 7 (Abuse of information leakage)

- ThreatCategory 8 (Abuse of authentication)

- ThreatCategory 9 (Lawful interception function abuse)

*Specific Threats:*

- SpecificThreat 1.1 (Manipulation of network configuration/data forging)

- SpecificThreat 1.2 (Exploitation of software, hardware vulnerabilities)

- SpecificThreat 1.3 (Denial of service (DoS))

- SpecificThreat 1.4 (Remote access exploitation)

- SpecificThreat 1.5 (Malicious code/software)

- SpecificThreat 1.6 (Abuse of remote access to the network)

- SpecificThreat 1.7 (Abuse of information leakage)

- SpecificThreat 1.8 (Abuse of authentication)

- SpecificThreat 1.9 (Lawful interception function abuse)

*Technologies:*

- Technology 1 (SDN, NFV, MANO)

- Technology 2 (RAN, RAT)

- Technology 3 (MEC)

- Technology 4 (API)

- Technology 5 (Cloud, Virtualisation)

*Relationships:*

- SpecificThreat 1.1 BelongsToCategory ThreatCategory 1

- SpecificThreat 1.2 BelongsToCategory ThreatCategory 2

- SpecificThreat 1.3 BelongsToCategory ThreatCategory 3

- SpecificThreat 1.4 BelongsToCategory ThreatCategory 4

- SpecificThreat 1.5 BelongsToCategory ThreatCategory 5

- SpecificThreat 1.6 BelongsToCategory ThreatCategory 6

- SpecificThreat 1.7 BelongsToCategory ThreatCategory 7

- SpecificThreat 1.8 BelongsToCategory ThreatCategory 8

- SpecificThreat 1.9 BelongsToCategory ThreatCategory 9

- SpecificThreat (1.1, 1.2, 1.3, 1.4, 1.5, 1.6, 1.7, 1.8, 1.9) TargetsTechnology Technology 1

- SpecificThreat (1.1, 1.2, 1.3, 1.4, 1.5, 1.6, 1.7, 1.8, 1.9) TargetsTechnology Technology 2

- SpecificThreat (1.1, 1.2, 1.3, 1.4, 1.5, 1.6, 1.7, 1.8, 1.9) TargetsTechnology Technology 3

- SpecificThreat (1.1, 1.2, 1.3, 1.4, 1.5, 1.6, 1.7, 1.8, 1.9) TargetsTechnology Technology 4

- SpecificThreat (1.1, 1.2, 1.3, 1.4, 1.5, 1.6, 1.7, 1.8, 1.9) TargetsTechnology Technology 5

This preliminary ontology provides a structured framework for understanding the relationships between different threat categories, specific threats, and the technologies they may target within the context of the TEN-T initiative implementation. Each specific threat is categorised, and its potential targets are identified, including the critical technologies involved.

Now, given this ontology, we can transform its equivalent into languages like RDF and OWL, as can be seen in Table 9 below.

**Table 9.** Representation of the ontology in both RDF and OWL formats.

RDF (Resource Description Framework):	OWL (Web Ontology Language):
<?xml version="1.0" encoding="UTF-8"?> <rdf:RDF xmlns:rdf="http://www.w3.org/1999/02/22-rdf-syntax-ns#" xmlns:rdfs="http://www.w3.org/2000/01/rdf-schema#"> <!-- Classes --> <rdf:Description rdf:about="#ThreatCategory"> <rdf:type rdf:resource="http://www.w3.org/2000/01/rdf-schema#Class"/> </rdf:Description> <rdf:Description rdf:about="#SpecificThreat"> <rdf:type rdf:resource="http://www.w3.org/2000/01/rdf-schema#Class"/> </rdf:Description> <rdf:Description rdf:about="#Technology"> <rdf:type rdf:resource="http://www.w3.org/2000/01/rdf-schema#Class"/> </rdf:Description> <!-- Relationships --> <rdf:Description rdf:about="#BelongsToCategory"> <rdf:type rdf:resource="http://www.w3.org/1999/02/22-rdf-syntax-ns#Property"/> <rdfs:domain rdf:resource="#SpecificThreat"/> <rdfs:range rdf:resource="#ThreatCategory"/> </rdf:Description> <rdf:Description rdf:about="#TargetsTechnology"> <rdf:type rdf:resource="http://www.w3.org/1999/02/22-rdf-syntax-ns#Property"/> <rdfs:domain rdf:resource="#SpecificThreat"/> <rdfs:range rdf:resource="#Technology"/> </rdf:Description> <!-- Individual Instances --> <rdf:Description rdf:about="#ThreatCategory1"> <rdf:type rdf:resource="#ThreatCategory"/> <rdf:Description rdf:about="#Name"> <rdf:type rdf:resource="http://www.w3.org/2000/01/rdf-schema#Literal"/> <rdf:value>Manipulation of network configuration/data forging</rdf:value> </rdf:Description> </rdf:Description> <!-- Add more instances for other Threat Categories, Specific Threats, and Technologies as needed --> </rdf:RDF>	<?xml version="1.0" encoding="UTF-8"?> <!DOCTYPE rdf:RDF [ <!ENTITY rdf "http://www.w3.org/1999/02/22-rdf-syntax-ns#"> <!ENTITY rdfs "http://www.w3.org/2000/01/rdf-schema#"> ]> <rdf:RDF xmlns:rdf="http://www.w3.org/1999/02/22-rdf-syntax-ns#" xmlns:rdfs="http://www.w3.org/2000/01/rdf-schema#"> <!-- Classes --> <rdf:Description rdf:about="#ThreatCategory"> <rdf:type rdf:resource="http://www.w3.org/2002/07/owl#Class"/> </rdf:Description> <rdf:Description rdf:about="#SpecificThreat"> <rdf:type rdf:resource="http://www.w3.org/2002/07/owl#Class"/> </rdf:Description> <rdf:Description rdf:about="#Technology"> <rdf:type rdf:resource="http://www.w3.org/2002/07/owl#Class"/> </rdf:Description> <!-- Relationships --> <rdf:Description rdf:about="#BelongsToCategory"> <rdf:type rdf:resource="http://www.w3.org/2002/07/owl#ObjectProperty"/> <rdf:domain rdf:resource="#SpecificThreat"/> <rdf:range rdf:resource="#ThreatCategory"/> </rdf:Description> <rdf:Description rdf:about="#TargetsTechnology"> <rdf:type rdf:resource="http://www.w3.org/2002/07/owl#ObjectProperty"/> <rdf:domain rdf:resource="#SpecificThreat"/> <rdf:range rdf:resource="#Technology"/> </rdf:Description> <!-- Individual Instances --> <rdf:Description rdf:about="#ThreatCategory1"> <rdf:type rdf:resource="#ThreatCategory"/> <rdf:Description rdf:about="#Name"> <rdf:type rdf:resource="http://www.w3.org/2002/07/owl#DatatypeProperty"/> <rdf:range rdf:resource="http://www.w3.org/2001/XMLSchema#string"/> <rdf:Description rdf:about="#value"> <rdf:type rdf:resource="http://www.w3.org/2002/07/owl#DatatypeProperty"/> </rdf:Description> </rdf:Description> <rdf:value>Manipulation of network configuration/data forging</rdf:value> </rdf:Description> <!-- Add more instances for other Threat Categories, Specific Threats, and Technologies as needed --> </rdf:RDF>

These examples provide the basic structure for RDF and OWL representations of the ontology. These can be expanded by adding more instances for other Threat Categories, Specific Threats, and Technologies as required.

The ontology, RDF, and OWL representations provide a structured way to define and represent threats and their relationships within the TEN-T implementation, making them valuable tools for managing and mitigating potential risks to the project. Consider their use for protecting the TEN-T implementation:

*Threat Identification:* The ontology categorizes and defines various threats, serving as a comprehensive reference for understanding relevant threats.
*Risk Assessment:* RDF/OWL representations enable the creation of a knowledge base that associates threats with the targeted technologies, allowing for detailed risk assessments, including severity levels, likelihood scores, and potential impacts.
*Vulnerability Analysis:* Potential vulnerabilities and weaknesses in technology components can be identified, involving specific configurations, software versions, and security measures.
*Mitigation Planning:* Develop a mitigation plan based on identified risks and vulnerabilities, which may include implementing security patches, enhancing access controls, and strengthening network monitoring.

Furthermore, in the interests of strengthening the resilience of the network, we can undertake the following:

*Incident Response:* Establish an incident response plan outlining steps to take during security incidents or breaches, along with defined roles and responsibilities.
*Continuous Monitoring:* Regularly monitor the security posture of the TEN-T implementation using security tools, intrusion detection systems, and log analysis to detect and respond to emerging threats.
*Security Awareness and Training:* Ensure the project team and stakeholders are well-informed about security best practices through training programs and awareness campaigns.
*Compliance and Regulation:* Stay up to date with relevant regulatory requirements and standards related to 5G and network infrastructure to ensure project compliance.
*Collaboration:* Engage with relevant stakeholders, including governmental agencies, law enforcement, and regulatory bodies, to coordinate efforts in addressing security threats and sharing threat intelligence.
*Adaptive Security:* Continuously adapt and improve security measures based on the evolving threat landscape by revisiting the ontology, updating threat assessments, and adjusting mitigation strategies.

Using the ontology and associated RDF/OWL representations, a comprehensive automated security framework tailored to the specific technologies and threats relevant to the TEN-T implementation can be created. This proactive approach to security helps safeguard the project and ensures its successful deployment across EU transport corridors.

These tools provide a structured and standardized way to represent and manage information related to threats, vulnerabilities, and mitigations, offering several benefits, for example: ontologies provide structured frameworks for categorizing threats, vulnerabilities, and their relationships; RDF and OWL representations offer semantic clarity, enhancing communication among stakeholders dealing with complex security concepts; OWL-based ontologies support automated reasoning and inference, identifying potential security implications and suggesting mitigations based on predefined rules; and RDF and OWL are widely recognized and standardized formats, facilitating integration with other systems, tools, and databases.

However, it's essential to consider the complexity, resource intensity, scalability, and required human expertise when undertaking such an approach for ontology development and RDF/OWL usage.

Significantly, the implementation of the ambitious 5G Action Plan for Europe is a multifaceted endeavor fraught with hurdles to be overcome. But the challenges associated with the implementation of the TEN-T initiative underscore the value of the Ontological-RDF-OWL approach. In enhancing Europe's transport corridors with 5G technology, requires seamless integration of 5G, NFV, SDN, MEC, Cloud Computing, Fog/Edge technologies. Moreover, various stakeholders across numerous transport corridors present complexities at every turn. Ensuring security, efficient resource allocation, and adherence to stringent regulatory frameworks are paramount. To address these challenges effectively, a holistic approach is required. The utilization of ontological representations, such as RDF and OWL, offers a structured and standardized means of threat analysis, risk mitigation, and real-time adaptation. By comprehensively mapping threats, assets, and dependencies, this approach empowers stakeholders to make informed decisions, proactively manage risks, and optimize the TEN-T implementation across the diverse European landscape.

## Part 3: Meeting Europe's cybersecurity challenges

For full compliance with the digital security requirements put forward in the call, ‘CEF-DIG-2022-5GSMARTCOM — 5G for Smart Communities,’ ^xviii^ hereafter referred as CEF – Digital, the various consortia proposing to outfit a 5G infrastructure across the TEN-T transportation corridors would have to address certain conditions contained in the Connecting Europe Facility – Digital sector (CEF) work programme, specifically, as put forward in ‘Section 3. Deployment of 5G infrastructures in Europe, 3.1 5G coverage along transport corridors.’ ^xix^

Cybersecurity risks related to suppliers are emphasized as these are more prevalent in 5G projects if the suppliers are based in third countries. The CEF guidance mandates that:

In the context of 5G networks, the role of suppliers has been identified in the EU coordinated risk assessment and the EU Toolbox on 5G cybersecurity as of particular relevance for cybersecurity. In particular, the Toolbox recommends assessing the risk profile of suppliers and applying appropriate restrictions - including necessary exclusions - for key assets considered as critical and sensitive. ^xx^

Moreover, the CEF guidance emphasizes that it is important to have efficient measures in place to tackle any security concerns that may arise. These measures should also encompass steps to prevent being bound by foreign jurisdiction obligations or influenced by third countries, whenever applicable.

In the CEF – Digital call, candidate consortia are asked not only to address issues associated with high-risk suppliers but required to describe how they will address the following digital security requirements, ^xxi^ notably:

• measures to promote supply chain resilience and strategic autonomy (in line with the 5G networks EU Toolbox of risk mitigating measures)
• security requirements for network operators (e.g. strict access controls, rules on secure operation and monitoring, limitations on outsourcing of specific functions, etc.)
• measures adopted to prevent the unsolicited transfer to, or access by, third parties of the data (personal or non-personal) stored or transported in the context of the project.

Furthermore, in order to be considered at all, proposals for funding must contain security declarations from the entities involved. These declarations should show that the project's network technologies and equipment (including software and services) that are financed by the project will adhere to the security requirements of the call, in line with the relevant EU law, national law, and EU cybersecurity guidance as can be found in the following official documents:

Commission Recommendation (EU) 2019/534 of 26 March 2019 Cybersecurity of 5G networks, C/2019/2335; the Report on EU Coordinated Risk Assessment of the Cybersecurity of 5G Networks of 9 October, 2019; the Council Conclusions on the Significance of 5G to the European Economy and the Need to Mitigate Security Risks Linked to 5G of 3 December, 2019; the Cybersecurity of 5G networks - EU Toolbox of Risk Mitigating Measures of 29 January, 2020; and COM(2020)50 of 29 January 2020 on Secure 5G deployment in the EU – implementing the toolbox. ^xxii^

Transitioning from the requirements outlined above to the real-world challenges of implementation is a formidable task. The call for proposals under the CEF – Digital initiative has set forth a comprehensive set of digital security requirements, reflecting the gravity and complexity of securing 5G networks within the European transport corridors. In this context, we embark on a meticulous examination of the European Union's guiding documents for 5G cybersecurity. These documents are not mere abstractions; they are the linchpins that bridge theory and practice, offering concrete solutions to the cybersecurity conundrum. As we delve into the specifics of each document, one overarching issue becomes apparent—the remarkable diversity of interpretations and responses by consortia that have sought funding in the CEF – Digital call in the early part of 2023. This inconsistency in approach, as we shall see, underscores the pressing need for a standardized ontological framework. Such a framework, capable of encoding the intricacies of these documents into RDF/OWL formats, has the potential to foster clarity, consistency, and, ultimately, more effective cybersecurity strategies.

A summary of the key aspects contained in the five pivotal EU documents referred to above are summarized below. These provide guidance and recommendations on ensuring the cybersecurity of 5G networks.

1. Commission Recommendation (EU) 2019/534 of 26 March 2019 Cybersecurity of 5G networks, C/2 335: ^xxiii^

This recommendation outlines guidelines for securing 5G networks across the EU. It highlights the need to identify and mitigate security risks throughout the 5G network lifecycle, including testing, certification, operation, and incident response. The goal is to ensure 5G networks are secure, resilient, and trustworthy components of critical EU infrastructure and services.

2. Report on EU Coordinated Risk Assessment of the Cybersecurity of 5G Networks of 9 October, 2019: ^xxiv^

This report comprehensively examines cybersecurity threats and vulnerabilities associated with 5G networks in the EU. It highlights risks including expanded attack surfaces, supply chain compromises, and state-sponsored threats. To address these, the report recommends enhanced security standards, supply chain transparency and accountability, and collaboration between stakeholders. Overall, it underscores the criticality of a coordinated, multi-stakeholder approach to securing 5G for EU digital transformation.

3. Council Conclusions on the Significance of 5G to the European Economy and the Need to Mitigate Security Risks Linked to 5G of 3 December, 2019: ^xxv^

These conclusions acknowledge 5G's importance for the EU economy while recognizing its security risks. They advocate a coordinated EU approach to 5G security, based on comprehensive risk assessments and resilience across the 5G supply chain. The conclusions also emphasize including industry, academia and civil society stakeholders in developing and deploying 5G security measures.

4. Cybersecurity of 5G networks - EU Toolbox of Risk Mitigating Measures of 29 January, 2020: ^xxvi^

This toolbox provides a framework of risk mitigation measures for Member States to secure 5G networks. It recommends network security controls, supply chain protections, network resilience measures, and emergency response plans. The goal ensures the security, resilience, and availability of 5G networks.

5. COM (2020)50 of 29 January 2020 on Secure 5G deployment in the EU – implementing the toolbox: ^xxvii^

This communication gives guidance for implementing the 5G toolbox measures to support secure 5G in the EU. It stresses resilient, EU-wide security approaches for 5G's critical applications like healthcare and transportation. Close industry collaboration on standards and product development is also key.

                                                                                                                                             ***

These core EU documents addressing cybersecurity requirements directed at consortia intent on submitting 5G-related project proposals to EC share the common objective of providing guidance to mitigate cybersecurity risks and ensure the secure, resilient, and trustworthy deployment of 5G networks across the European Union.

However, it is important to note that the common thread emanating from these five security-related documents maps directly to the requirements put forward in the CEF - Digital call, as identified earlier at the beginning of this section. These are:

- Managing engagement with high-risk suppliers through due diligence, restrictions, and avoiding over-dependence.
- Ensuring resilience of the 5G supply chain and strategic autonomy of the EU by minimizing risks that could compromise integrity and independence.
- Empowering and enabling network operators to effectively protect 5G networks through resources, technologies, and training.
- Implementing robust measures to prevent unauthorized access to or transfer of personal and non-personal data in 5G networks, including encryption, access controls, and compliance with data regulations.

*Unpacking EU's communication COM (2020)50 and 5G security toolbox*

At this stage, it is imperative for us to delve more deeply into the contents of two of the aforementioned documents. Specifically, No. 5 COM (2020)50 and the latest news concerning the EU Toolbox of Risk Mitigating Measures (2020). This is because the Communication is especially noteworthy in the manner in which it lays out a comprehensive framework of measures aimed at bolstering the secure deployment of 5G networks within the European Union (EU), together with its identification of the transportation use case as being of vital importance in the context of ensuring the security and resilience of 5G networks in critical infrastructure sectors.

This Communication builds upon the EU Toolbox of Risk Mitigating Measures for 5G Networks, also published on January 29, 2020, and provides essential guidance to EU member states on the practical implementation of the toolbox measures and other pertinent security requisites. The Communication addresses the four principal commonalities that we have identified across all five documents by:

i) Addressing Engagement with High-Risk Suppliers: The communication recognizes the security implications arising from member states engaging with high-risk suppliers. It underscores the significance of prudently managing such engagements to safeguard the integrity of 5G networks.
ii) Ensuring Resilience of 5G Networks, Supply Chains, and Strategic Autonomy: In alignment with the objectives of the EU, the communication not only underscores the resilience of 5G networks but also emphasizes the need to preserve supply chain resilience and strategic autonomy. It acknowledges that certain risks have the potential to compromise the resilience of 5G networks, as well as supply chains, and even impact the strategic autonomy of EU Member States. The measures advocated in the communication serve to enhance transparency, accountability, and security within the supply chain while endorsing independent third-party assessments to reinforce its robustness.
iii) Empowering Network Operators: The communication acknowledges the existence of risks that might impede network operators from effectively safeguarding their 5G networks. It highlights the imperative of empowering network operators to surmount these challenges and bolster the security, resilience, and availability of 5G networks.
iv) Addressing the criticality of data security: The communication emphasizes measures adopted to prevent the unsolicited transfer to, or access by, third parties of both personal and non-personal data that is stored or transported as part of 5G network projects. This highlights the EU's commitment to safeguarding data privacy and integrity in the 5G ecosystem, reinforcing the importance of comprehensive security in all dimensions of 5G deployment.

Moreover, the Communication accentuates the critical importance of ensuring the security and resilience of 5G networks in the EU, given their pivotal role in supporting various vital sectors like healthcare, transportation, and energy. It delineates a series of key actions for member states, encompassing the development of a national 5G risk assessment and mitigation strategy, the establishment of a certification framework for 5G suppliers, and the enhancement of EU-level cooperation in matters of 5G security.

It further underscores the need for a coordinated, EU-wide approach to address the security risks associated with 5G networks. Recognizing the transnational nature of this technology and the potential consequences of security breaches, it calls for close collaboration with industry stakeholders to devise and implement security measures, ensuring the availability of secure 5G products and services.

*The Importance of COM(2020)50 for the TEN-T Initiative*

Ensuring the security and resilience of 5G networks within the TEN-T (Trans-European Transport Network) initiative is of paramount importance to the EU. The TEN-T initiative aims to create a seamless and efficient transport network across Europe, serving vital sectors such as healthcare, transportation, and energy as identified in COM(2020)50. The success and reliability of the entire TEN-T infrastructure depends on the following key factors:

Reliable Connectivity: In the context of the TEN-T initiative, 5G networks are the backbone for various applications and services, including real-time monitoring of transportation systems, intelligent traffic management, and emergency response systems. Ensuring their security and resilience guarantees reliable connectivity, which is essential for the safety and efficiency of transportation corridors.
Resilience in Critical Scenarios: 5G networks must be resilient to withstand cyber threats and physical disruptions. This resilience is crucial during emergency situations and natural disasters, where the transportation network's uninterrupted functionality is a matter of public safety.
Supply Chain Security: The supply chain for 5G infrastructure components, including hardware and software, must be secure to prevent vulnerabilities in the network's architecture. This aligns with the supply chain resilience aspect mentioned in COM(2020)50 and contributes to the overall reliability of the TEN-T initiative.
Strategic Autonomy: Security measures also enhance strategic autonomy, ensuring that European nations have control over their critical infrastructure. This is particularly significant in an era where technology dependencies can impact national sovereignty.
Data Protection: The TEN-T initiative involves the exchange of vast amounts of data, both personal and non-personal, to manage and optimize transportation processes. Security measures, as emphasized in COM(2020)50, protect this data from unauthorized access and transfer, thereby safeguarding privacy and preventing potential disruptions in data flow.

In summary, the security and resilience of 5G networks in the TEN-T initiative are vital for maintaining the efficiency, safety, and integrity of Europe's transportation networks. These measures not only align with the objectives set forth in COM(2020)50 but also guarantee the continued success of the initiative and its contributions to the European economy and society.

*Second progress report on the implementation of the EU Toolbox on 5G cybersecurity*

EU Member States’ authorities (NIS Cooperation Group), with the support of the European Commission and ENISA, the EU Agency for Cybersecurity, published a second progress report on the implementation of the EU Toolbox on 5G cybersecurity. ^xxviii^ This announcement of June 15, 2023, signifies notable advancements by Member States in this endeavor. However, it is evident that there remains room for further improvements in several areas. The report aptly recognizes the challenges that Member States confront while executing the toolbox's provisions. Notably, these challenges encompass the absence of standardized cybersecurity prerequisites across the European Union and the scarcity of cybersecurity expertise.

The Commission's Communication accompanying this report delineates a comprehensive strategy designed to fortify Member States' efforts in executing the toolbox effectively. These strategies encompass the development of a cohesive cybersecurity framework for the European Union, the provision of comprehensive training and support mechanisms for cybersecurity professionals, and substantial investments in cybersecurity research and development.

This latest progress report marks a pivotal milestone in the journey toward enhancing the cybersecurity of 5G networks within the European Union. Notable findings within the report underscore the following key points:

- Member States have undertaken a series of initiatives aimed at translating the EU Toolbox for 5G cybersecurity into practical action. These include the formulation of national cybersecurity strategies and the establishment of national coordination bodies.
- There exists commendable cooperation among Member States, characterized by the exchange of critical information and the sharing of best practices concerning cybersecurity.

Nevertheless, Member States continue to grapple with an array of challenges during the toolbox's implementation. These persisting challenges include the absence of uniform cybersecurity requisites throughout the European Union and the prevailing shortage of cybersecurity professionals. Furthermore, some Member States have not yet adopted or fully implemented all the necessary legal or regulatory measures, some have not yet completed their risk assessments or mitigation plans, and some have not yet taken concrete steps to diversify their 5G suppliers or reduce their dependency on high-risk suppliers.

In light of these findings, the report puts forward a series of noteworthy recommendations, highlighting the ways in which Member Staes need to persist in their efforts to implement the EU Toolbox for 5G cybersecurity; collaborate in the development of standardized cybersecurity prerequisites tailored for 5G networks; invest in cybersecurity education and training programs to address the skills gap effectively; actively support research and development endeavors in the realm of cybersecurity, recognizing the pivotal role these play in bolstering digital resilience.

 Moreover, the same progress report also deals comprehensively with the risk and threat categories already examined, mirroring the consistent focus on the four key risk factors embodied in the EC documents dedicated to ensuring the cybersecurity of 5G networks. The progress report goes on in much detail in this regard, however, these risk factors will be explored in much more detail later when we consider the question of cybersecurity taxonomy and ontology. For now, consider that the report highlights Member State initiatives across four key risk dimensions:

i) Member States are identifying high-risk suppliers based on EU assessments, enforcing access restrictions to critical assets, and working to reduce dependency on high-risk suppliers.
ii) Member States are focused on boosting supply chain resilience and strategic autonomy through R&D investments, supporting domestic suppliers, and promoting cooperation.
iii) Network operators are implementing security requirements like strict access controls, rules for secure operations and monitoring, and limitations on outsourcing certain functions.
iv) Data protection steps like encryption, access controls, and security monitoring are being implemented to prevent unauthorized data access or transfer.

Significantly, the report does reflect continued progress by Member States in executing the EU Toolbox measures to address risks around suppliers, supply chain, network security, and data protection as part of ensuring robust 5G cybersecurity.

### Charting the path forward: strengthening Europe's cybersecurity in the 5G era

The European Union's steadfast dedication to fortifying its digital infrastructure through the CEF – Digital initiative is of paramount importance to sustain a viable and secure 5G network across all the EU. Nevertheless, our thorough exploration of the key cybersecurity documents underscores the challenges associated with the intricate journey toward implementation. The inherent complexity of these documents, including their quantity, naturally leads to diverse interpretations and responses, presenting a formidable challenge to EU consortia applying for project funding from the EC. Herein lies the pivotal role of an ontological approach.

More specifically, ontology can play a pivotal role in harmonizing the diverse interpretations of cybersecurity documents, thereby streamlining the implementation process and ensuring a more uniform approach to cybersecurity across the EU. At the heart of the challenges lies the difficulty in achieving a common understanding and approach among diverse stakeholders, which is key for effective implementation and security measures. An ontological approach, therefore, serves a pivotal role by providing a structured and unified framework that translates complex cybersecurity requirements into a universally understandable and actionable format. This not only aids in streamlining the application process for EC funding but also ensures that all parties are aligned in their understanding and approach to cybersecurity, ultimately enhancing the resilience and effectiveness of the 5G network across the EU.

The creation of a comprehensive cybersecurity ontology, meticulously encoded in RDF/OWL formats, and seamlessly integrated into the operations of consortia seeking funding, would signify a monumental stride forward. Beyond a theoretical construct, this ontological framework would stand as a practical and pragmatic response to an immediate need. Its potential lies in harmonizing varied interpretations, aligning collaborative efforts, and forging a united defence against cybersecurity threats.

As Europe stands at the cusp of a 5G revolution poised to redefine the digital landscape, the significance of cybersecurity cannot be overlooked. It transcends theory; it is a concrete solution to the pressing challenges at hand. By embracing this approach, we empower consortia to navigate the complexities of the digital era with confidence and coherence, ensuring the success of the 'CEF-DIG-2022-5GSMARTCOM' initiative and the safeguarding of Europe's digital future.

### ENISA’S role in risk identification and mitigation in 5G networks

This section provides an overview of the contributions made by ENISA to enhancing the cybersecurity of 5G networks with special attention to the role of Risk.

*ENISA: 5G cybersecurity standards in relation to policy*

ENISA's report on 5G Cybersecurity Standards ^13^ highlights the critical role of standardization in managing risks and bolstering trust in the 5G ecosystem. This comprehensive report covers technical and organizational aspects, analyzing over 150 security measures and 140 documents to identify gaps in standardization.

The report suggests a progressive approach to 5G standardization, emphasizing the need for tailored standards and their alignment with strategic objectives. It calls for harmonizing risk assessment practices across all 5G stakeholders to enhance risk identification and mitigation.

While technical and organizational standards contribute to 5G security, the report underscores that they are not exhaustive. Given 5G's complexity, a holistic view of risk and resilience is vital, independent of network configurations. Risk remains a central focus in our discussion.

*ENISA's EECC security measures guideline*

ENISA's guideline ^14^ addresses Article 40 of the European Electronic Communications Code (EECC), focusing on risk management for electronic communications providers and competent authorities. This article mandates appropriate technical and organizational measures to mitigate security risks to networks and services. The guideline offers detailed guidance on risk identification and specific security measures.

Article 40 of the EECC sets rigorous security standards for electronic communications providers, giving competent authorities the power to enforce them (Article 41). This aligns with the broader goals of the EECC to modernize EU telecom regulations and establish a common regulatory framework, ensuring secure and reliable 5G coverage for all EU citizens.

*ENISA's 5G security supplement under EECC*

This supplement ^15^ builds upon the previous guideline, offering additional guidance to competent authorities and network providers regarding security measures for 5G networks. It highlights the unique security challenges posed by 5G, including heightened complexity, the use of new technologies like virtualization and network slicing, and increasing reliance on 5G networks for critical infrastructure applications, making them attractive targets for cyber threats.

The guidance emphasizes specific risk-mitigation measures, such as secure network design, robust monitoring, audits, and employee awareness. Additionally, it includes informative annexes covering 5G security threats, controls, and best practices.

*ENISA: Security controls in 5G SA 3GPP specifications*

This document ^16^ offers a comprehensive overview of security controls specified in 3GPP standards for 5G Standalone (SA) networks. ^xxix^

It begins by outlining 5G SA architecture and the security threats facing these networks. Key security controls include:

1. Authentication and Authorization: Ensuring only authorized users and devices access 5G SA networks, as defined in 3GPP specifications.

2. Integrity and Confidentiality: Protecting data integrity and confidentiality through encryption, integrity checks, and tampering prevention.

3. Availability: Ensuring network and service availability with load balancing, DoS attack mitigation, and self-healing mechanisms.

4. Audit and Accountability: Tracking and auditing user and device activity with logs, security reports, and real-time monitoring.

The report provides detailed analysis for each control, explaining its function, threat mitigation, implementation challenges, and offering recommendations for 5G SA network deployments.

                                                                                                                                             ***

We note here that implementing the TEN-T project, which involves critical transportation infrastructure, would benefit from the use of 5G SA (Standalone) architecture, wherever feasible. 5G SA provides several advantages, which are summarized in Table 10 below. Overall, 5G SA architecture aligns well with the requirements of the TEN-T project, making it a suitable choice for improving and expanding transportation infrastructure across the European Union.

**Table 10.** Benefits of 5G SA networks.

Benefit	Description
Independence	No existing infrastructure reliance; ideal for greenfield projects, offering more flexibility in network design and deployment.
Low Latency	Ultra-low latency is crucial for real-time communication, coordination, traffic management, and safety systems in transportation applications.
High Capacity	Smart transportation systems, including autonomous vehicles, IoT devices, and high-definition video streaming, require higher capacity and data rates.
Reliability	Ensures high reliability and availability for transportation systems where downtime can have serious consequences.
Security	Critical infrastructure requires enhanced security features to protect against cyber threats.

In the analysis of the ENISA reports, we have examined the intricate landscape of 5G cybersecurity with a steadfast emphasis on risk. These reports serve as practical guides, directing stakeholders through the multifaceted challenges of securing 5G networks and services. Throughout, risk emerges as the central theme. It underpins the imperative for comprehensive security controls, necessitates ongoing vigilance, and propels proactive measures. As elucidated by ENISA, understanding and managing risk is not a mere facet of 5G security; it constitutes the foundational framework upon which secure, resilient, and dependable 5G networks are erected. In our pursuit of robust 5G infrastructure to support the TEN-T implementation, risk mitigation remains our guiding principle, illuminating the path toward a prototype ontology.

### Towards a prototype ontology

In a previous paper ^1^, I showed how the ENISA 5G Threat Landscape Report ^2^ revealed ENISA’s painstaking efforts to stand out among other leading-edge players in the 5G arena and achieve its strategic aims of integrating cybersecurity considerations with threats, risks, and vulnerabilities into an architecture of 5G right from the start of the design and development process. Taking into account an excerpt from that paper, we will now turn to how ENISA’s treatment of Risks to 5G infrastructure, might help us move towards a workable Ontology.

**Figure 4.**
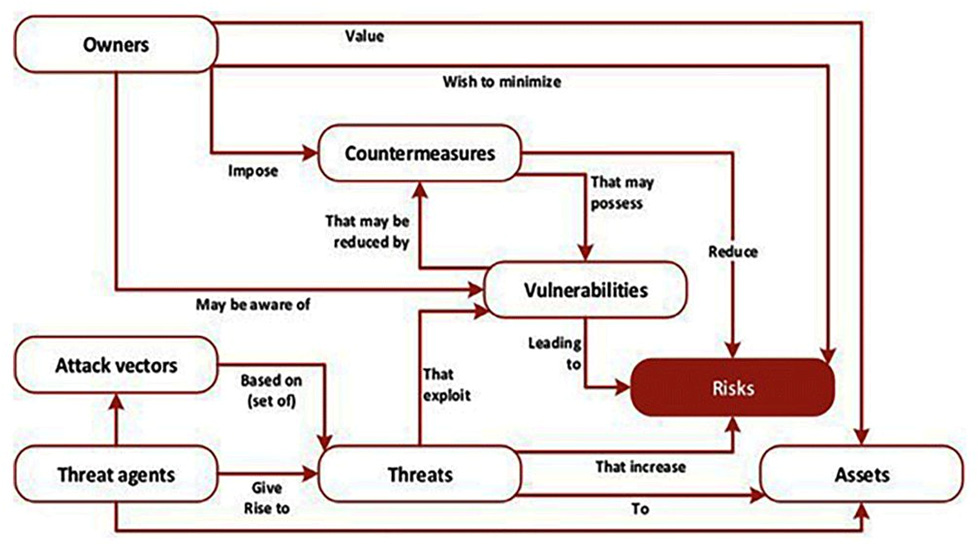
The multifaceted aspects of risks. The various elements of cyberthreats, vulnerabilities, assets and their relationship to risks are depicted in the figure; all are woven into the design and architecture of the ENISA framework. © European Union Agency for Cybersecurity (ENISA), 2020.

The power of ENISA’s recommendations comes into play when dealing with cybersecurity risks. The relationships that are depicted in Figure 4 ^2, p. 12^ are based on the ISO 27005 standard and connect vulnerabilities directly with threats and risks in relation to owners and assets, attack vectors, threat agents, and countermeasures. These are integrated into the overall design and architecture of the ENISA framework and can easily form part of a prototype Ontology for representing 5G cybersecurity risks.

We have already suggested that creating a standard ontological framework for EU consortia applying for EC 5G project funding would provide a consistent and uniform context to support the 5G Action Plan for Europe, which would be particularly helpful for implementing the 5G infrastructure that could support the TEN-T transportation corridors across the EU. The hierarchy associated with risks presented below will now serve as a framework for our prototype Ontology.

Consider the ontological hierarchy of risks as depicted in the figure.

*Classes:*

- Risk
- Owner
- Threat
- Vulnerability
- Asset
- Control
- Countermeasure
- Attack vector

*Relationships:*

- Is owned by: A risk is owned by one or more owners.
- Is mitigated by: A risk is mitigated by a control or countermeasure.
- Exploits: A threat exploits a vulnerability.
- Impacts: A threat impacts an asset.
- Protects: A control protects an asset from a threat.
- Enables: An attack vector enables a threat to exploit a vulnerability.

*Attributes:*

- Risk: Likelihood, impact, severity, exposure
- Owners: Responsibility, authority
- Threat: Agent, capability, motivation
- Vulnerability: Type, severity, exploitable
- Asset: Value, criticality, sensitivity
- Control: Type, effectiveness, cost
- Countermeasure: Type, effectiveness, cost
- Attack vector: Type, severity, exploitability

*Sub-Classes/Sub-Elements* (of above named Classes):

(Risk)

- Strategic risk: A risk that could have a significant impact on the organization's overall strategy or objectives.
- Operational risk: A risk that could impact the organization's day-to-day operations.
- Financial risk: A risk that could impact the organization's financial performance.
- Reputational risk: A risk that could damage the organization's reputation.

(Owners)

- Risk owner: The person or team who is primarily responsible for managing a risk.
- Risk manager: A person or team who is responsible for developing and implementing risk management strategies and processes.
- Stakeholder: A person or group who has an interest in the outcome of a risk management process.

(Threat)

- Natural threat: A threat caused by a natural event, such as a hurricane or earthquake.
- Man-made threat: A threat caused by human activity, such as a cyberattack or terrorist attack.

(Vulnerability)

- Technical vulnerability: A weakness in a system or application that could be exploited by a threat.
- Process vulnerability: A weakness in a process or procedure that could be exploited by a threat.
- Human vulnerability: A weakness in human behavior or knowledge that could be exploited by a threat.

(Asset)

- Information asset: Data that is valuable to the organization.
- Physical asset: Property or equipment that is valuable to the organization.
- People asset: The organization's employees or customers.

(Control)

- Preventive control: A control that aims to prevent a threat from exploiting a vulnerability.
- Detection control: A control that aims to detect a threat that has exploited a vulnerability.
- Corrective control: A control that aims to recover from the impact of a threat that has exploited a vulnerability.

(Countermeasure)

- Technical countermeasure: A countermeasure that uses technology to reduce the likelihood or impact of a risk.
- Procedural countermeasure: A countermeasure that uses policies and procedures to reduce the likelihood or impact of a risk.
- Training countermeasure: A countermeasure that provides training to employees to reduce the likelihood or impact of a risk.

(Attack vector)

- Technical attack vector: An attack vector that exploits a technical vulnerability.
- Process attack vector: An attack vector that exploits a process vulnerability.
- Human attack vector: An attack vector that exploits a human vulnerability.

### RDF / OWL formulation

Significantly, a standard ontological framework for EU consortia applying for EC 5G project funding would provide a consistent and uniform basis for formulating project plans to address the requirements of the CEF - Digital call and more broadly the 5G Action Plan for Europe. This would be particularly helpful for defining and implementing the security measures necessary to safeguard the 5G infrastructure that would support the TEN-T transportation corridors across the EU. The RDF/OWL code presented below is a preliminary step towards achieving a full ontological framework that could eventually support the 5G Action Plan.

As a start, let us employ the hierarchy of risks described above as a basis for coding the RDF/OWL representation of our ontology. The structure outlined in the risk hierarchy provides a workable framework for representing various aspects of risks, including their ownership, mitigation, and relationships with threats, vulnerabilities, assets, controls, countermeasures, and attack vectors.

To represent this ontology in RDF/OWL format, we would need to create classes, properties, relationships, and attributes based on the structure formulated. Below is a high-level overview of how we could represent some of these elements in RDF/OWL format:

*Classes:*

We would define classes for each of the entities mentioned, such as Risk, Owner, Threat, Vulnerability, Asset, Control, Countermeasure, and Attack vector. We would also create subclasses for specific types of risks, owners, threats, vulnerabilities, assets, controls, countermeasures, and attack vectors.


<!-- Define classes for various entities -->
<rdf:Description rdf:about="#Owner">
 <rdf:type rdf:resource="http://www.w3.org/2002/07/owl#Class"/>
</rdf:Description>


*Properties:*

We would define properties to represent the relationships between entities, such as "Is owned by," "Is mitigated by," "Exploits," "Impacts," "Protects," and "Enables."


<!-- Define properties for relationships -->
<rdf:Description rdf:about="#isOwnedBy">
 <rdf:type rdf:resource="http://www.w3.org/2002/07/owl#ObjectProperty"/>
</rdf:Description>



<!-- Define domains and ranges for properties -->
<rdf:Description rdf:about="#isOwnedBy">
 <rdf:type rdf:resource="http://www.w3.org/2002/07/owl#ObjectProperty"/>
 <rdfs:domain rdf:resource="#Risk"/>
 <rdfs:range rdf:resource="#Owner"/>
</rdf:Description>


*Attributes:*

We would create data properties to represent attributes associated with entities, such as "Likelihood," "Impact," "Severity," "Exposure," etc.


<!-- Define data properties for attributes -->
<rdf:Description rdf:about="#likelihood">
 <rdf:type rdf:resource="http://www.w3.org/2002/07/owl#DatatypeProperty"/>
 <rdfs:domain rdf:resource="#Risk"/>
 <rdfs:range rdf:resource="http://www.w3.org/2001/XMLSchema#float"/>
</rdf:Description>


*Sub-Classes/Sub-Elements:*

We would create subclasses as per our ontology hierarchy to represent specific types of entities.


<!-- Define subclasses for Risk -->
<rdf:Description rdf:about="#Risk">
 <rdf:type rdf:resource="http://www.w3.org/2002/07/owl#Class"/>
</rdf:Description>

<rdf:Description rdf:about="#StrategicRisk">
 <rdf:type rdf:resource="http://www.w3.org/2002/07/owl#Class"/>
 <rdfs:subClassOf rdf:resource="#Risk"/>
</rdf:Description>


These code examples represent a first step in what could be a lengthy process. We would still need to extend this structure to represent all the classes, properties, relationships, and attributes in our ontology.

As well, consider that in RDF/OWL, URIs (Uniform Resource Identifiers) ^xxx^ are used to uniquely identify entities, so we will need to define appropriate URIs for each class, property, and instance in our ontology. What is more, we note that the actual RDF/OWL code for our Risks ontology could be extensive, and we may need to use ontology editing tools or libraries to create and manage the ontology effectively.

### 5G Action Plan and TEN-T transportation corridors

It is important to note that risks faced by an organization may vary, which can impact the hierarchy of risks. Thus, creating a standard ontological framework for EU consortia applying for EC funding would provide a consistent and uniform infrastructure to support the 5G Action Plan for Europe, which would be particularly helpful for implementing the 5G infrastructure that could support the TEN-T transportation corridors across the EU. The ontological hierarchy of risks presented above is just one example of a possible framework.

Our previously proposed approach for implementing the 5G Action Plan in Europe involved deploying 5G base stations along transport corridors, using network slicing to create virtual networks, implementing VNFs for various network functions, employing NFV Management and Orchestration (MANO), incorporating SDN controllers to manage network traffic, deploying Multi-Access Edge Computing (MEC) nodes at strategic points along the corridors, implementing security VNFs, ensuring coordinated orchestration between NFV MANO and SDN controllers, utilizing cloud computing, deploying FOG/EDGE infrastructure at strategic points along the transport corridors, and collaborating with multiple traditional cloud providers to establish a federated cloud model that can ensure redundancy and high availability.

The ontological approach to risk management provides a systematic way to identify, assess, and mitigate risks. It is based on a well-defined conceptual framework that can be used to model and reason about risks.

The ontological hierarchy of risks can be used to integrate risk management into the 5G Action Plan in the following ways:

*Risk identification*: The ontological hierarchy of risks can be used to identify all of the potential risks to the 5G Action Plan. This can be done by enumerating all of the assets, threats, and vulnerabilities that are relevant to the plan.
*Risk assessment*: The ontological hierarchy of risks can be used to assess the likelihood and impact of each risk. This can be done by considering the factors that contribute to each risk, such as the severity of the threat, the vulnerability of the asset, and the effectiveness of the controls in place.
*Risk mitigation*: The ontological hierarchy of risks can be used to develop and implement risk mitigation strategies. This can be done by identifying and implementing controls that reduce the likelihood or impact of each risk.

The following is an example of how the ontological hierarchy of risks can be used to assess and mitigate a specific risk to the 5G Action Plan:

*Risk:* Loss of confidentiality of user data due to a cyberattack on the 5G network.
*Threat:* Cyberattack by a malicious actor.
*Vulnerability*: Security vulnerability in the 5G network.
*Asset:* User data.
*Control:* Implement strong security measures, such as encryption and intrusion detection systems.

The ontological hierarchy of risks can also be used to monitor and improve risk management over time. By tracking the status of risks and the effectiveness of controls, project consortia can identify and address new risks as they emerge.

The ontological approach to risk management is a powerful tool that can be used to improve the security and reliability of the 5G Action Plan. By integrating risk management into the plan from the outset, organizations can reduce the likelihood and impact of risks, and ensure that the plan is successful.

Here are some additional examples of how the ontological hierarchy of risks can be used to integrate risk management into the 5G Action Plan:

1.

*Risk:* Failure of a critical component of the 5G network, leading to disruption of service.
*Threat:* Natural disaster, cyberattack, or technical failure.
*Vulnerability:* Single point of failure in the 5G network.
*Asset:* 5G network infrastructure.
*Control:* Design the 5G network with redundancy and diversity to reduce the risk of single points of failure.

*2.*

*Risk:* Delay in the deployment of the 5G network, leading to a competitive disadvantage.
*Threat:* Technical challenges, regulatory delays, or opposition from stakeholders.
*Vulnerability:* Complex and distributed nature of the 5G network.
*Asset:* 5G network infrastructure and deployment schedule.
*Control:* Develop a comprehensive deployment plan and identify and mitigate potential risks and delays early on.

By using the ontological hierarchy of risks to identify, assess, and mitigate risks, consortia can improve the security, reliability, and efficiency of their TEN-T implementations.

### The European Cybersecurity Taxonomy

The European Commission, through its technical entity Joint Research Council (JRC), has developed a common cybersecurity taxonomy for the EU ^17^. Although the taxonomy has potential, it does not yet represent a complete cybersecurity ontology.

The potential of the JRC's cybersecurity taxonomy, especially when compared to ENISA's 5G Threat Taxonomy, lies in its broader scope and its specific focus on categorizing existing institutions and expertise across Europe. This comprehensive categorization is pivotal for creating an interconnected cybersecurity framework within the EU. Moreover, its machine-readable format, coded in RDF, offers a robust foundation for developing an ontology specifically tailored to 5G infrastructure in the context of cybersecurity. The potential, therefore, extends beyond mere categorization; it includes the taxonomy's adaptability and suitability for evolving into a more complex and interconnected ontology. This machine-readable format facilitates automation and integration with various cybersecurity tools and platforms, enhancing the practical utility of the taxonomy in real-world applications.

The JRC's taxonomy, while advanced in its scope and format, does not yet encapsulate a complete cybersecurity ontology, particularly for 5G infrastructure, because of the specific demands of an ontology in this context. For an ontology to be fully functional, it must go beyond categorization and include intricate relationships and dependencies specific to 5G cybersecurity. This involves a detailed understanding and representation of the unique threats, vulnerabilities, and countermeasures associated with 5G networks. While JRC's taxonomy provides a strong starting point with its machine-readable format and comprehensive categorization, the transition to a full-blown ontology requires building upon these features to develop a framework that encapsulates the dynamic and complex nature of 5G cybersecurity. This includes integrating specific 5G taxonomical structures and ensuring the ontology is sufficiently detailed and dynamic to adapt to the rapidly evolving 5G cybersecurity landscape.

To address this, we suggest taking several critical steps towards transitioning from a cybersecurity taxonomy to a proper ontology with special attention given to 5G infrastructure and the EU’s TEN-T initiative during the initial phases. Our aim is to put forward a tentative foundation that will lead to ensuring the resilience and security of Europe's digital infrastructure.

**Figure 5.**
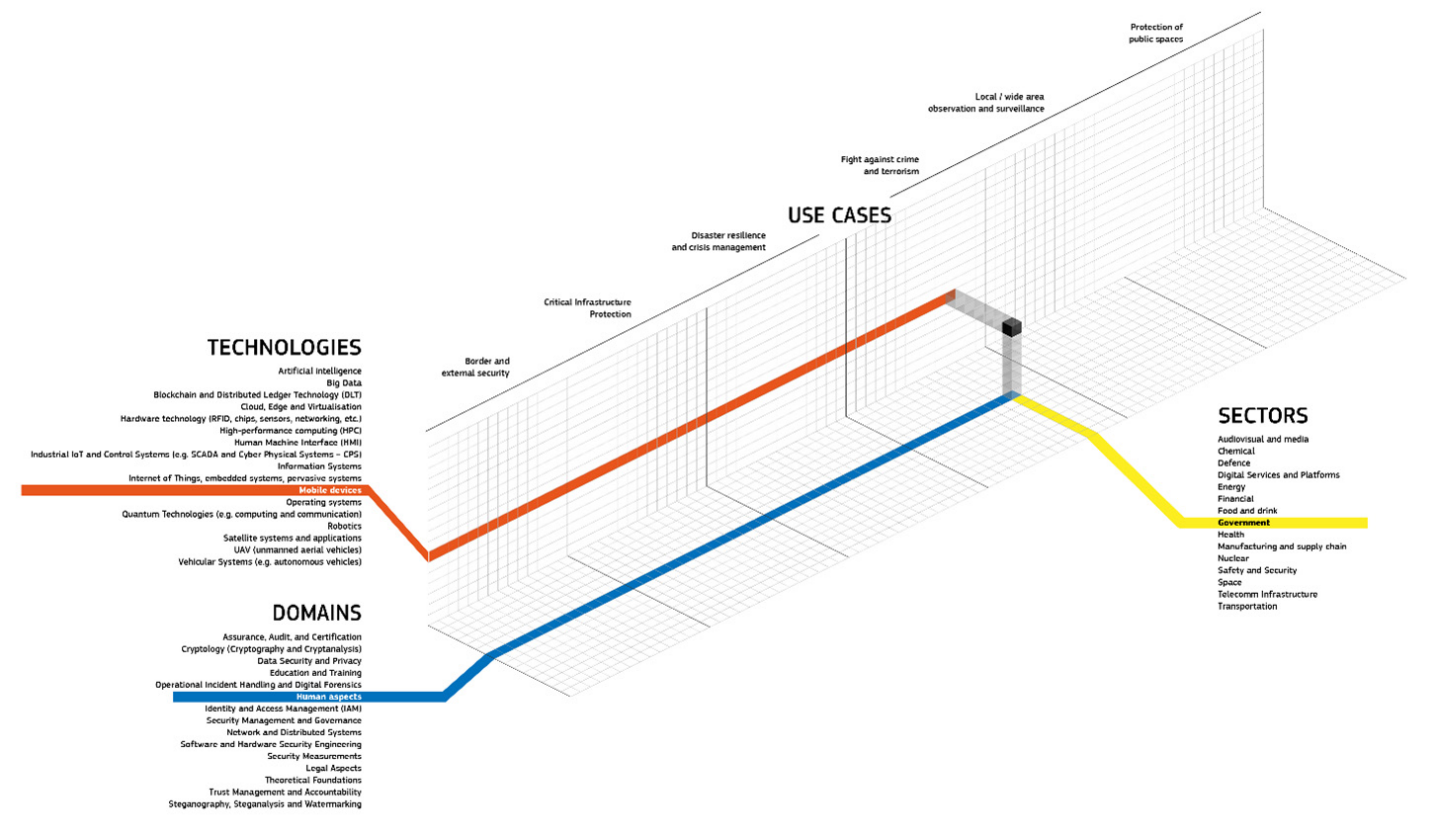
The specific Technologies, Domains, Use Cases and Sectors are represented by Mobile Devices (red) and Human Aspects (blue) and Government (yellow). ^xxxi^ For our purposes we can interpret the figure as connecting Mobile Device Technologies and Domain category, Human Aspects (any of Automating security functionality, Cybersecurity profiling, Enhancing risk perception, Human-related risks/threats, Direct Human perception of cybersecurity, User acceptance of security policies and technologies) ^xxxii^ to Sector category Government (EU, EC, Member States). As far as other categories are concerned, we could connect any of those in red – Cloud, Edge and Virtualisation; Hardware Technology, Mobile Devices; or those in blue – Cryptology, Data Security and Privacy, Network and Distributed Systems to that in yellow – Transportation. © European Union 2019.

JRC's European Cybersecurity Taxonomy ^18^ aligns cybersecurity definitions and terminologies to categorize existing institutions and expertise across Europe. As depicted in Figure 5, the taxonomy is based on four main dimensions: knowledge domains, sectors, technologies, and use cases, and can be represented as a three-dimensional visual representation.

A simplified version of JRC’s taxonomical structure ^xxxiii^ can be represented as follows:

***1. Cybersecurity Taxonomy*** ^xxxiv^

- …
- Hardware Technology
- …
- Manufacturing and Supply Chain
- …
- Mobile Devices
- Network and Distributed Systems
- …
- Telecomm Infrastructure
- …

***2. Knowledge Domains***

- …
- Network and Distributed Systems
- Distributed Systems Security Analysis and Simulation
- Distributed Systems Security
- Network Security (Principles, Methods, Protocols, Algorithms, and Technologies)
- Network Attack Propagation Analysis
- Network Layer Attacks and Mitigation Techniques
- Network Interoperability
- Network Security Requirements
- Resilience Aspects
- Data Security and Privacy
- Software and Hardware Security Engineering
- Symmetric Cryptography
- Asymmetric Cryptography
- Cryptanalysis Methodologies, Techniques, Tools
- Cryptography Mathematical Foundations
- …

***3. Sector***

- **…**
- Transportation (Aligns with TEN-T implementation)
- …

***4. Technology***

- …
- Mobile Devices
- …
- Hardware Technology
- …
- Cloud Edge Virtualization (Aligns with TEN-T implementation)

***5. Use Case***

- **…**
- Critical Infrastructure Protection (create a new use case for TEN-T)
- …

### JRC Cybersecurity Taxonomy as target for addressing CEF – Digital requirements

JRC’s Cybersecurity Taxonomy provides a work-in-progress framework to address the CEF – Digital cybersecurity requirements of our TEN-T implementation, namely, our four risk categories: i) Involvement of High-Risk Suppliers; ii) Measures to Promote Supply Chain Resilience and Strategic Autonomy; iii) Security Requirements for Network Operators; and iv) Measures to Prevent Unsolicited Data Transfer or Access.

Let us consider these targets in the JRC Taxonomy:

i) Involvement of High-Risk Suppliers:

   Cybersecurity Taxonomy

Hardware Technology (High-risk suppliers of hardware technology) ^xxxv^
  Manufacturing and Supply Chain (High-risk suppliers in the supply chain) ^xxxvi^
  Telecomm Infrastructure ^xxxvii^ (High-risk suppliers in telecommunications infrastructure) ^xxxviii^

ii) Measures to Promote Supply Chain Resilience and Strategic Autonomy:

   Cybersecurity Taxonomy

Manufacturing and supply chain ^xxxix^
  Mobile Devices ^xxxx^

iii) Security Requirements for Network Operators:

   Knowledge Domains

Network and Distributed Systems ^xxxxi^
  • Requirements for network security ^xxxxii^

iv) Measures to Prevent Unsolicited Data Transfer or Access aligns with:

   Knowledge Domains

Data Security and Privacy ^xxxxiii^
  • Data usage control ^xxxxiv^

Having examined the foundations of the European Cybersecurity Taxonomy, and how it aligns with the specific requirements outlined in the CEF - Digital call, we will now illustrate how our proposed ontology seamlessly integrates with the target taxonomy, aligning the essential cybersecurity elements needed for consortia seeking funding for 5G TEN-T infrastructure projects. Our goal is to demonstrate how this synergy can address the critical aspects of managing high-risk suppliers, ensuring the resilience of the 5G supply chain, empowering network operators, and implementing robust data security measures. Let's proceed with the task of mapping our ontology to the JRC Cybersecurity Taxonomy, paving the way for a comprehensive approach to secure and resilient 5G networks within the framework of Europe's TEN-T initiative.

### Mapping Ontology to JRC Cybersecurity Taxonomy for 5G TEN-T Projects with RDF

To establish proper relationships between our prototype ontology and the JRC Cybersecurity Taxonomy in the context of the requirements for consortia seeking funding for 5G TEN-T infrastructure projects we begin by mapping the concepts and categories in our ontology to the relevant categories in the JRC Cybersecurity Taxonomy. The RDF code below establishes these relationships as follows:

1. Managing Engagement with High-Risk Suppliers:

   - In our ontology, we create a subclass of class Owner that represents "Supplier," and "High-risk Supplier" as a subclass of “Supplier.”

   - We also establish a relationship between our ontology's "Risk" and "Supplier" (and "High-risk Supplier") to indicate that certain risks are associated with suppliers.

# Creating subclasses for "Supplier" and "High-risk Supplier"
ex:Supplier rdf:type owl:Class ;  rdfs:subClassOf ex:Owner.
ex:HighRiskSupplier rdf:type owl:Class ;  rdfs:subClassOf ex:Supplier.
ex:Risk ex:hasAssociatedSupplier ex:Supplier.
ex:Risk ex:hasAssociatedSupplier ex:HighRiskSupplier.

   - We now link this relationship to the JRC Cybersecurity Taxonomy. In this scenario, distinct threats are outlined individually, representing specific types of risks. The "hybrid" nature of these threats emerges when they are combined to target Europe's vital 5G infrastructure along TEN-T corridors. The alignment between the ontology and the JRC Cybersecurity Taxonomy effectively identifies these threats, triggers appropriate preventive measures under the "Countermeasure" class, and empowers network operators with cybersecurity best practices from the "Control" class, ultimately ensuring the resilience of Europe's digital infrastructure against the multifaceted challenges posed by hybrid threats.

   - We then link risks involving suppliers to the JRC Cybersecurity Taxonomy, Telecomm Infrastructure category.

# Linking "Supplier" and "High-risk Supplier" to JRC Cybersecurity Taxonomy
ex:Supplier rdfs:subClassOf
<http://data.jrc.ec.europa.eu/ontology/cybersecurity/telecomm_infrastructure>.
ex:HighRiskSupplier rdfs:subClassOf
<http://data.jrc.ec.europa.eu/ontology/cybersecurity/telecomm_infrastructure>.

(Similar code would be constructed above for “Hardware Technology,” and “Manufacturing and Supply Chain.”)

2. Ensuring Resilience of the 5G Supply Chain and Strategic Autonomy:

   - In our ontology, we create a class related to "Supply Chain Resilience" and "Strategic Autonomy."

   - We then establish a relationship between our ontology's "Risk" and "Supply Chain Resilience" and "Strategic Autonomy" to indicate risks related to these aspects.

ex:SupplyChainResilience rdf:type owl:Class.
ex:StrategicAutonomy rdf:type owl:Class.
ex:Risk ex:hasSupplyChainResilience ex:SupplyChainResilience.
ex:Risk ex:hasStrategicAutonomy ex:StrategicAutonomy.

   - We now link these relationships to the JRC Cybersecurity Taxonomy's "Manufacturing and Supply Chain" and "Mobile Devices" categories to show alignment with measures to promote supply chain resilience and strategic autonomy.

ex: SupplyChainResilience rdfs:subClassOf
 <http://data.jrc.ec.europa.eu/ontology/cybersecurity/manufacturing_supply_chain>
ex: StrategicAutonomy rdfs:subClassOf
 <http://data.jrc.ec.europa.eu/ontology/cybersecurity/mobile_devices>

3. Empowering and Enabling Network Operators:

   - In our ontology, we create a subclass under "Owner" named "Network Operator."

   - We then establish a relationship between our ontology's "Risk" and "Network Operator" to indicate risks that network operators may face.

ex:NetworkOperator rdf:type owl:Class ;  rdfs:subClassOf ex:Owner.
ex:Risk ex:hasNetworkOperator ex:NetworkOperator.

   - We link this relationship to the JRC Cybersecurity Taxonomy's “Requirements for network security” within "Network and Distributed Systems" category to show alignment with security requirements for network operators.

ex: NetworkOperator rdfs:subClassOf
<http://data.jrc.ec.europa.eu/ontology/cybersecurity/network-security_requirements>

4. Implementing Robust Measures to Prevent Unauthorized Access to or Transfer of Data:

   - In our ontology, we create a subclass under "Control" named "Data Security Measures."

   - We also establish a relationship in our ontology between "Risk" and "Data Security Measures" to indicate risks related to data security.

ex:DataSecurityMeasures rdf:type owl:Class ;  rdfs:subClassOf ex:Control.
ex:Risk ex:hasDataSecurityMeasures ex:DataSecurityMeasures

   - We link this relationship to the appropriate categories in the JRC Cybersecurity Taxonomy: i.e., Data usage control within Data Security and Privacy;

ex: DataSecurityMeasures rdfs:subClassOf
<http://data.jrc.ec.europa.eu/ontology/cybersecurity/data_usage_control>

By creating these relationships and mappings, we can effectively connect our ontology to the relevant categories in the JRC Cybersecurity Taxonomy, illustrating how our ontology aligns with the specific requirements outlined for consortia seeking funding for 5G TEN-T infrastructure projects. This approach helps establish a clear connection between our ontology and the JRC Cybersecurity Taxonomy, enhancing understanding and communication within the context of the funding requirements.

### The hybrid threat context

We began our earlier discussion with an approach for constructing a prototype ontology that is based on the hierarchy of risks embodied in ISO 27005 ^xxxxv^ as applied by ENISA in the context of 5G.

However, it is important to note that ISO 27005 is a general-purpose standard for information security risk management. While it is often applied in cybersecurity contexts to manage risks related to information systems and data, its principles and framework can be adapted to other contexts, including dealing with hybrid threats.

The key strength of ISO 27005 lies in its systematic approach to identifying, assessing, and managing risks. It provides a structured methodology that can be tailored to the specific needs and objectives of an organization or project. This adaptability makes it suitable for addressing a wide range of risks, including those associated with hybrid threats.

We have already examined how such threats typically involve a combination of conventional and non-conventional methods, often spanning different domains such as cyber, political, economic, and military. ISO 27005's risk management process can be extended to consider these diverse elements when assessing and managing risks associated with hybrid threats. Organizations and projects facing hybrid threats can use ISO 27005 as a foundation and then incorporate additional context-specific elements and risk factors into their risk assessment and management processes.

Indeed, ISO 27005 is a versatile standard that provides a solid foundation for risk management and can be adapted to various contexts, including addressing risks related to hybrid threats when supplemented with domain-specific knowledge and considerations.

How the ontological hierarchy of risks can be used to integrate risk management into the 5G Action Plan, and taking into account the notion of possible hybrid threats, can be understood as follows:

### Scenario: safeguarding Europe's 5G infrastructure against hybrid threats

*Background*:

In the not-so-distant future, Europe heavily relies on its advanced 5G infrastructure, especially along the major TEN-T transportation corridors. However, this critical infrastructure is exposed to a comprehensive set of threats, which combine conventional and cyber methods to undermine its integrity and security. This scenario highlights the four critical requirements outlined in the CEF - Digital call (i-iv).

*Hybrid Threat context:*

i) Managing Engagement with High-Risk Suppliers:

   - Threat: Infiltration by a malicious state-sponsored entity into trusted suppliers' networks, compromising 5G infrastructure integrity.

ii) Ensuring Resilience of the 5G Supply Chain:

   - Threat: Disinformation campaigns targeting supply chain stakeholders, disrupting the production and delivery of critical 5G components.

iii) Empowering Network Operators:

   - Threat: Coordinated cyberattacks exploiting vulnerabilities in network operator services.

iv) Implementing Robust Data Security Measures:

   - Threat: A state-sponsored entity orchestrates unauthorized access to sensitive data through intermediaries, seeking to exploit personal and non-personal data.

*Response and Resilience:*

In this scenario, distinct threats are outlined individually, representing specific types of risks. The "hybrid" aspect comes into play when these individual threats converge to create a multifaceted attack. In the ontology-JRC Taxonomy alignment, risk assessments automatically take shape in the context of the sub-classes of "Risk" (refer to section ‘Towards a prototype ontology’ above), including their potential to be part of a hybrid threat scenario. The "Countermeasure" class triggers preventive measures, ensuring the security of the supply chain, safeguarding against disinformation, protecting network operator services, and fortifying data security.

*Infiltration by a malicious state-sponsored entity into trusted suppliers' networks*

In this threat scenario, we also consider the possibility of a malicious state-sponsored entity attempting to compromise the integrity of 5G infrastructure by infiltrating the networks of trusted suppliers. This threat can have severe consequences for the security and resilience of 5G networks, as it involves a covert and sophisticated adversary seeking to exploit vulnerabilities within the supply chain.

Risk Description:

The risk here involves the compromise of trusted suppliers' networks, which may provide essential components or services to 5G infrastructure projects. The compromise could lead to the insertion of malicious elements, the theft of sensitive information, or the disruption of the supply chain. The potential consequences include the compromise of 5G network integrity, data breaches, and operational disruptions.

Recall that the principal Classes in our ontology are Risk, Owner, Threat, Vulnerability, Asset, Control, Countermeasure, and Attack vector.

Resilience Measures:

To enhance resilience against this threat while leveraging our ontology and the JRC Cybersecurity Taxonomy, consortia seeking funding for 5G TEN-T projects can implement the following measures:

1. Supplier Classification: Utilize our ontology to classify suppliers (subClass of Owner) based on risk levels. High-risk suppliers (subclass of Supplier) can undergo more stringent vetting and monitoring.

This classification can be aligned with the JRC Taxonomy's categories "Hardware Technology," "Manufacturing and Supply Chain," and "Telecomm Infrastructure".

2. Threat Intelligence Integration: Integrate threat intelligence feeds, based on the threat categories in our ontology, aligned with JRC Taxonomy's cyber monitoring systems, yet to be defined. This would allow for real-time assessment of potential threats to supplier networks.

3. Risk Assessment Framework: Develop a risk assessment framework using our ontology to model the relationships between suppliers, assets, and vulnerabilities. This framework can align with the JRC Taxonomy's relevant categories, yet to be determined.

4. Incident Response Planning: Utilize our ontology to map out incident response procedures specific to supplier compromises. This ensures that countermeasures are consistent and aligned with the threat context.

Countermeasures:

In the event of an infiltration or suspected compromise, consortia can leverage our ontology and the JRC Taxonomy to guide countermeasure strategies:

1. Ontology-Driven Incident Response: Our ontology can provide a structured framework for incident response, mapping out the affected assets, vulnerabilities, and controls. The ontology’s threat categories will have defined relationships to appropriate countermeasures, which in turn will be aligned with relevant taxonomical elements.

2. Resilience Enhancement: By continually updating the ontology with information from the data collection elements defined in the JRC Taxonomy, consortia can enhance their overall resilience by staying informed about emerging threats and effective countermeasures.

3. Cross-Referencing: The ontology can enable cross-referencing between supplier classifications, threat categories, and risk assessments. This helps consortia make informed decisions about supplier relationships and the allocation of resources for security measures.

By incorporating our ontology and the JRC Cybersecurity Taxonomy into the consortium's cybersecurity framework, the risk posed by the infiltration of trusted supplier networks can be addressed systematically. This integrated approach enhances the consortium's ability to identify, respond to, and mitigate threats against trusted suppliers effectively, ultimately bolstering the security and resilience of 5G infrastructure within the context of TEN-T projects.

The "hybrid" nature of these threats emerges when they are combined to target Europe's vital 5G infrastructure along TEN-T corridors. The alignment between the ontology and the JRC Cybersecurity Taxonomy effectively identifies these threats, triggers appropriate preventive measures under the "Countermeasure" class, and empowers network operators with cybersecurity best practices from the "Control" class, ultimately ensuring the resilience of the 5G supply chain and Europe's digital infrastructure against the multifaceted challenges posed by hybrid threats.

## Conclusions

This exposition represents a significant step forward in understanding and managing hybrid threats within the context of 5G mobile networks. It achieves this through a systematic exploration and integration of various elements: the incorporation of ENISA’s threat taxonomy into an ontology for 5G cybersecurity, insights into EU cybersecurity strategies, and considerations of the broader implications of 5G technology in the context of the Connecting Europe Facility (CEF) work plan, the 5G Action Plan for Europe, and specifically within that the TEN-T initiative.

The paper successfully delineates the complexities of the 5G threat landscape, categorizing various cybersecurity challenges and presenting a nuanced understanding of hybrid threats. It emphasizes the importance of a machine-readable ontology, utilizing RDF and OWL coding techniques, to facilitate automated analysis and decision-making in cybersecurity.

The study also underscores the critical role of 5G in European infrastructure and the need for robust cybersecurity measures in line with EU directives and funding programmes. It highlights the importance of understanding and addressing the cybersecurity implications of 5G technology, especially in the context of hybrid threats, and proposes a structured approach to manage these risks effectively.

To be sure, it is important to highlight the successful integration of Resource Description Framework (RDF) coding in aligning the developed ontology with the JRC Cybersecurity Taxonomy. This article marks a pivotal advancement in the field of 5G cybersecurity by meticulously aligning a comprehensive ontology, crafted to understand and manage hybrid threats in 5G networks, with the JRC Cybersecurity Taxonomy. This alignment is achieved through the effective application of RDF coding techniques, which enhances the ontology's machine-readability and interoperability.

The integration of RDF allows for a nuanced and detailed representation of the relationships and attributes within the ontology, ensuring a high degree of precision and clarity in the categorization of cyberthreats and their countermeasures. This approach not only facilitates a better understanding of the complex cybersecurity landscape within 5G networks but will allow for automated analysis and decision-making, critical for responding to dynamic cyberthreats in real-time.

Moreover, the successful RDF coding signifies a breakthrough in harmonizing different cybersecurity frameworks and standards, demonstrating a practical methodology for bridging gaps between various cybersecurity taxonomies and ontologies. This harmonization is particularly significant in the context of aligning with EU standards and directives, as it provides a coherent framework for policymakers, security experts, and stakeholders in the 5G ecosystem to collaboratively enhance the resilience and security of 5G infrastructures.

Overall, the article's contribution through the effective use of RDF in aligning the ontology with the JRC Cybersecurity Taxonomy represents a substantial stride in securing 5G networks against hybrid threats, aligning with European cybersecurity strategies, and setting a precedent for future research and practice in the domain of 5G cybersecurity.

Moving forward, the focus will be on refining and expanding the ontology to cover emerging threats and technologies in the 5G domain. Future work will also explore the integration of this ontology into real-world cybersecurity systems and the development of automated tools for threat detection and response. Collaborative efforts with EU regulatory bodies and cybersecurity agencies will be essential in advancing these objectives, ensuring that the ontology remains relevant and effective in the ever-evolving landscape of 5G cybersecurity.

## Data Availability

The description of the JRC Cybersecurity Taxonomy ^xxxxvi^ contains a link to the file consisting of the RDF code (data) provided by JRC and characterizing categories 1 – 5 in section ‘The European Cybersecurity Taxonomy’ above. That file contains 400 pages; 21,172 words; and 13,285 lines of RDF code. ^xxxxvii^ The data associated with the RDF code is covered by the European Commission Reuse and Copyright Notice © European Union, 1995–2023 which states that reuse is authorized, provided the source is acknowledged. The reuse policy of the European Commission is implemented by a *Decision of 12 December 2011*. ^xxxxviii^

## Footnotes

^i^ A general overview of what constitutes the idea of hybrid threats is nicely put by The European Centre of Excellence for Countering Hybrid Threats (Hybrid CoE): https://www.hybridcoe.fi/hybrid-threats-as-a-phenomenon/

^ii^ See ITU-T X.1205, TELECOMMUNICATION STANDARDISATION SECTOR OF ITU (04/2008) SERIES X: DATA NETWORKS, OPEN SYSTEM COMMUNICATIONS AND SECURITY Telecommunication security Overview of cybersecurity, https://www.itu.int/rec/T-REC-X.1205-200804-I

^iii^ The Resource Description Framework (RDF) is a standard from the World Wide Web Consortium (W3C) originally designed as a data model for metadata. It has come to be used as a general method for the description and exchange of graph data. https://www.w3.org/RDF/

^iv^ OWL is a semantic markup language for publishing and sharing ontologies on the World Wide Web. It has been developed as a vocabulary extension of RDF and is intended to facilitate interpretability among web content using vocabulary and formatting that allows automatic machine processing. OWL Web Ontology Language Reference (w3.org) and OWL 2 Web Ontology Language Quick Reference Guide (Second Edition) (w3.org)

^v^ Are 5G Networks Setting The Stage For A New Wave Of Cyberattacks? (forbes.com)

^vi^ Why 5G requires new approaches to cybersecurity | Brookings

^vii^ Analysis of 5G Network Security Reveals Attack Possibilities - Infosecurity Magazine (infosecurity-magazine.com)

^viii^ One of 5G’s Biggest Features Is a Security Minefield | WIRED

^ix^ Cyber Intelligence | Europol (europa.eu)

^x^ See Common Taxonomy for Law Enforcement and CSIRTs | Europol (europa.eu)

^xi^ Convention on Cybercrime

^xii^ Directive on Attacks against Information Systems

^xiii^ Internet Organised Crime Threat Assessment (IOCTA) | Europol (europa.eu)

^xiv^ Europe’s 5G Action Plan | Shaping Europe’s digital future (europa.eu)

^xv^ Trans-European Transport Network (TEN-T) (europa.eu)

^xvi^ With network slicing, TEN-T could allocate specialized slices for different transportation modes, optimizing traffic management, and enhancing user experiences.

^xvii^ The ability of MEC to bring cloud resources closer to the users could revolutionize real-time applications within the transport system, enabling seamless monitoring, data processing, and ensuring low-latency responses.

^xviii^ Funding & tenders (europa.eu)

^xix^ See ‘ANNEX to the Commission Implementing Decision on the financing of the Connecting Europe Facility – Digital sector and the adoption of the multiannual work programme for 2021-2025,’ pp. 11-18; Brussels, 6.12.2021 C(2021) 9463 final. Multiannual work programme for 2021-2023 - Connecting Europe Facility - Digital | Shaping Europe’s digital future (europa.eu)

^xx^ Ibid., p. 18.

^xxi^ Funding & tenders (europa.eu)

^xxii^ As quoted in CEF – Digital, p. 18, footnote 28.

^xxiii^ COMMISSION RECOMMENDATION (EU) 2019/ 534 - of 26 March 2019 - Cybersecurity of 5G networks (europa.eu)

^xxiv^ EU-wide coordinated risk assessment of 5G networks security | Shaping Europe’s digital future (europa.eu)

^xxv^ Council Conclusions on the significance of 5G to the European Economy and the need to mitigate security risks linked to 5G2019/C 414/03 - Publications Office of the EU (europa.eu)

^xxvi^ Cybersecurity of 5G networks - EU Toolbox of risk mitigating measures | Shaping Europe’s digital future (europa.eu)

^xxvii^ Secure 5G deployment in the EU: Implementing the EU toolbox - Communication from the Commission | Shaping Europe’s digital future (europa.eu)

^xxviii^ Full press release and Commission announces next steps on cybersecurity of 5G networks in complement to latest progress report by Member States | Shaping Europe’s digital future (europa.eu)

^xxix^ 5G SA refers to a fully independent 5G network architecture that does not rely on or require the presence of existing 4G or LTE infrastructure. In this architecture, 5G networks operate autonomously, providing enhanced capabilities and performance compared to non-standalone modes. It allows for the full realization of 5G's potential, including low latency, high data rates, and support for a wide range of applications and services.

^xxx^ A Uniform Resource Identifier is a compact string of characters that uniquely identifies a particular resource, whether it's a document, a web page, a concept, or any other item that can be accessed via the internet. URIs are used to provide a standardized and unambiguous way to reference resources on the web or within computer systems. They serve as addresses or labels for identifying and locating resources, making it possible to access and interact with them in a consistent manner.

^xxxi^ Cybersecurity Taxonomy | European Cybersecurity Atlas (europa.eu)

^xxxii^ Cybersecurity taxonomy - EU Vocabularies - Publications Office of the EU (europa.eu)

^xxxiii^ JRC Cybersecurity Taxonomy - EU Vocabularies - Publications Office of the EU (europa.eu) contains the link to the RDF code delineating categories 1. – 5. cybersecurity-taxonomy-skos-ap-eu.rdf (The RDF file contains 400 pages; 21,172 words; 13,285 lines.)

^xxxiv^ In JRC's taxonomy, the categories directly under Cybersecurity Taxonomy loosely mirror those identified in the Knowledge Domains category. European Commission publishes EU Cybersecurity Taxonomy | Shaping Europe’s digital future (europa.eu)

^xxxv^ Hardware technology - EU Vocabularies - Publications Office of the EU (europa.eu)

^xxxvi^ Manufacturing and supply chain - EU Vocabularies - Publications Office of the EU (europa.eu)

^xxxvii^ Telecomm Infrastructure - EU Vocabularies - Publications Office of the EU (europa.eu)

^xxxviii^ Includes companies and internet service providers, as well as the infrastructures required to realise such communications.

^xxxix^ Includes a vast range of supply chain activities and production techniques, from small-scale enterprises using traditional production techniques to very large enterprises sitting atop a high and broad pyramid of parts and components suppliers collectively manufacturing complex products (e.g. system or product integration). Manufacturing and supply chain - EU Vocabularies - Publications Office of the EU (europa.eu)

^xxxx^ Supporting domestic mobile device suppliers. Mobile devices - EU Vocabularies - Publications Office of the EU (europa.eu)

^xxxxi^ Network and Distributed Systems - EU Vocabularies - Publications Office of the EU (europa.eu)

^xxxxii^ Requirements for network security - EU Vocabularies - Publications Office of the EU (europa.eu)

^xxxxiii^ Cybersecurity taxonomy - EU Vocabularies - Publications Office of the EU (europa.eu)

^xxxxiv^ Data usage control - EU Vocabularies - Publications Office of the EU (europa.eu)

^xxxxv^ ISO/IEC 27005:2018 pdf download - Free Standards Download
