# Decoding 5G security: toward a hybrid threat ontology

**DOI:** 10.12688/openreseurope.16916.3

**Published:** 2025-10-22

**Authors:** R. Andrew Paskauskas

**Affiliations:** 1 Lithuanian Cybercrime Center of Excellence for Training, Research and Education (L3CE), Vilnius, 08393, Lithuania

**Keywords:** 5G Cybersecurity, Hybrid Threats, Ontology Development, RDF (Resource Description Framework), ENISA Threat Taxonomy, EU Cybersecurity Strategies, Automated Analysis in Cybersecurity

## Abstract

This Open Letter announces a new initiative, designed from the ground up, incorporating key cybersecurity standards while providing a novel framework for modelling hybrid threats in 5G infrastructure. The 5G Hybrid Threat Ontology is a structured framework designed to reduce risk and achieve sustainable resilience against hybrid threats targeting 5G infrastructure. As fifth-generation (5G) networks become integral to critical infrastructure, they introduce new vulnerabilities that adversaries can exploit through hybrid threats—multifaceted attacks spanning cyber, physical, and socio-political domains. Existing security approaches focus on specific technical vulnerabilities or predictive threat modelling but lack a unified framework to address hybrid threat scenarios systematically.

This paper advances the study of 5G security by proposing an ontology-driven approach prioritising resilience through risk reduction rather than threat elimination or prediction. Developed using Semantic Web technologies, specifically the Resource Description Framework (RDF) in Turtle representation for structured threat modelling, the ontology ensures machine-readability, structured threat intelligence sharing, and validation through Shapes Constraint Language (SHACL). It integrates established frameworks, including the 5G Threat Taxonomy compiled by the European Union Agency for Cybersecurity (ENISA), the Structured Threat Information eXpression (STIX) standard, and other widely used cybersecurity standards.

By formalising relationships between adversarial tactics, 5G vulnerabilities, and cascading risks, the ontology enables semantic reasoning using tools within the Protégé framework. Although still under development, the 5G Hybrid Threat Ontology demonstrates strong Description Logic (DL) expressivity, ensuring adaptability for evolving security challenges. This approach bridges high-level policy directives with operational cybersecurity needs, reinforcing a resilience-driven security posture. Future research will build on the phased development of the 5G Hybrid Threat Ontology to enhance adaptive threat modelling and dynamic risk assessment, ensuring that the ontology continues to evolve as a strategic tool for strengthening the resilience of 5G infrastructures against hybrid threats.

## Introduction

This Open Letter presents a structured ontological approach to hybrid threats in 5G security, aligning with Open Research Europe (ORE) guidelines on policy-relevant contributions. By addressing the intersection of cybersecurity, resilience, and risk reduction, this work provides a foundational framework for safeguarding essential services and reinforcing the resilience of critical infrastructures within the European Union (EU).

Hybrid threats exploit vulnerabilities across multiple domains—cyber, physical, and socio-political—creating cascading effects that undermine the security and continuity of essential services. In the context of 5G networks, these threats pose a significant risk due to the technology’s role as a backbone for critical infrastructure operations. The CER Directive explicitly mandates that critical entities identify and assess risks to essential services, implement appropriate security measures, and develop resilience plans to ensure continuity. ^i^ Similarly, the NIS2 Directive strengthens security and resilience requirements for network and information systems that underpin essential services. ^ii^ To meet these objectives, NIS2 emphasises advanced monitoring, risk assessment, and modelling techniques to prevent, detect, and mitigate cyber threats affecting critical infrastructures. Both directives recognise the evolving landscape of hybrid threats, particularly in the 5G domain, where cyber and physical vulnerabilities intersect. By emphasising advanced risk assessment and resilience planning, CER and NIS2 provide a regulatory foundation for mitigating these multi-domain threats.

The increasing complexity of hybrid threats—combining cyber operations, disinformation campaigns, and geopolitical coercion—demands a systematic, intelligence-driven approach to security and resilience. Addressing these evolving risks requires structured knowledge representation that enables threat intelligence modelling, dynamic risk assessment, and cross-sectoral security planning. This necessitates interoperability across different security frameworks, ensuring that hybrid threats can be assessed using a unified and structured approach.

Against this backdrop, this paper introduces an ontology-driven approach to modelling hybrid threats in 5G infrastructure. Ontologies provide a formalised structure for representing threat intelligence, enabling interoperability, automated reasoning, and resilience-based security planning. This work ensures alignment with recognised security standards while extending them to model cross-domain hybrid threat scenarios affecting critical infrastructures.

Our proposed ontology systematically represents threat actors, attack vectors, vulnerabilities, and mitigation strategies, allowing for structured risk assessment and strategic security planning in compliance with EU resilience mandates. This work contributes to a more secure and reliable environment for essential services across Europe by focusing on risk reduction, multi-hazard modelling, and data-driven decision-making. Specifically, the ontology’s structured classification of hybrid threats facilitates multi-hazard modelling by capturing the interdependencies between diverse attack vectors and their cascading effects.

## Open Letter to the research community

As an Open Letter addressing policy-relevant issues, this work serves three essential functions defined by Open Research Europe (ORE). First, it examines key European policies on hybrid threats, cybersecurity, and resilience. Second, it proposes an ontological framework supporting policy-driven risk management and resilience planning. Third, it announces a new initiative—the 5G Hybrid Threat Ontology—and details its development, validation, and future trajectory.

The paper begins by introducing the concept of hybrid threats in the context of 5G security, explaining how these threats span cyber, physical, and socio-political domains. From there, it situates the ontology within European security policy frameworks, highlighting its alignment with major institutional players, cybersecurity frameworks, and standards organisations.

A key focus is placed on the ontology development process, which unfolded in four iterative phases. These phases are structured to demonstrate the ontology’s expansion, integration of external knowledge sources, and increasing representational complexity. The case study on 5G-enabled threats to U.S. election infrastructure 2024 reflects the ontology’s capabilities, illustrating how it captures evolving hybrid threat scenarios.

Validation plays a central role in this initiative, and the paper outlines various validation procedures, reasoning tests, and ontology metrics analyses that ensure structural consistency and expressivity. The concluding discussion turns toward future directions, emphasising the potential to expand the ontology’s capabilities by incorporating dynamic datasets, enabling adaptive risk assessment, and enhancing resilience-driven security planning.

This initiative remains a work in progress, and by presenting it in an Open Letter format, we aim to encourage continued refinement, collaboration, and engagement from the broader research and security community.

This work contributes to the broader effort to protect essential services and critical infrastructures from hybrid threats by bridging the gap between high-level European resilience strategies and practical cybersecurity applications. Integrating formal ontologies and cybersecurity knowledge graphs provides a scalable, adaptable framework for policymakers, security professionals, and researchers working on threat intelligence, risk assessment, and cyber-resilience strategies. This aligns with ongoing European cybersecurity initiatives, including the European Centre of Excellence for Countering Hybrid Threats (Hybrid CoE) ^1^, which promotes resilience-based strategies for countering multi-domain threats. It is to Hybrid CoE’s pivotal role in defining all aspects of the hybrid threat phenomena that we now turn.

## Hybrid threats in context: 5G and the cyber domain

Hybrid threats represent a confluence of coercive and subversive activities, combining conventional and unconventional methods to destabilise targets. These threats leverage diverse political, diplomatic, military, economic, and technological domains and often exploit critical information systems, mainly through sophisticated cyber-attacks. In the cyber domain, hybrid threats increasingly target 5G infrastructure and associated technologies. With their unprecedented speed, low latency, and reliance on software-defined and virtualised components, these networks introduce new attack surfaces, and hybrid adversaries exploit vulnerabilities to disrupt essential services such as telecommunications, transport, energy, and financial systems, thereby magnifying the impact of their operations. These cascading disruptions underscore the need for structured, resilience-based security strategies capable of mitigating cross-domain impacts.

Hybrid CoE provides a general overview of hybrid threats ^1^. Such threats operate through clandestine actions designed to evade detection, attribution, and retaliation. State and non-state actors deploy these tactics to exploit societal, institutional, and technological vulnerabilities, undermining democratic values and public trust. This includes psychological operations to create confusion, deepen societal divisions, and erode confidence in governance through disinformation, fake news, and AI-driven propaganda campaigns.

Strategically, hybrid threats can function in two distinct phases:

- *Priming phase:* Adversaries subtly monitor and influence the target, strengthening their tools and assets while maintaining plausible deniability. This involves reconnaissance, testing vulnerabilities, and deploying covert influence campaigns.
- *Operational phase:* This escalates to overt actions, such as direct cyber-attacks on critical infrastructure, resulting in widespread disruption, erosion of societal stability, and potential long-term damage to public trust and institutional credibility.

The complexity and interconnected nature of hybrid threats in 5G networks demand a structured approach to their analysis and understanding. While existing frameworks provide valuable insights into specific threats, they lack a unified model that captures the hierarchical relationships between threat components and their dynamic evolution within the 5G ecosystem. This paper develops such a model, building upon established frameworks while addressing the unique characteristics of hybrid threats in 5G networks. To bridge this gap, we propose an ontology-driven framework that formalises these interdependencies, integrating cyber and non-cyber threat dimensions to enhance cross-sectoral security planning.

## Hybrid threats and 5G security

In the cyber realm, particularly in the context of 5G, hybrid threats exploit the critical reliance on high-speed, low-latency communication networks to target infrastructure, manipulate information flows, and disrupt vital services.

Hybrid threats in 5G networks are characterised by:

- *Complex cyber-attacks:* These include advanced persistent threats (APTs), ransomware, supply chain compromises, and attacks on core network functionalities such as 5G slicing, virtualisation, and radio access network (RAN) components.
- *Disinformation and propaganda:* Leveraging 5G's ubiquity to amplify social media's reach, adversaries engage in targeted disinformation campaigns to influence public opinion, deepen societal divisions, and destabilise democratic processes.
- *Exploitation of technological vulnerabilities:* Hybrid actors exploit vulnerabilities inherent in 5G's open architecture, software-defined networks, and reliance on third-party suppliers, creating potential backdoors for surveillance and sabotage.
- *Proxy actors and plausible deniability:* State and non-state actors often collaborate covertly – deployment of proxies to execute cyber operations allows for plausible deniability and avoidance of direct attribution.

In the context of 5G, hybrid threats aim to:

- Undermine critical information and operational systems in healthcare, energy, transportation, and finance industries.
- Disrupt supply chains by compromising software, hardware, or service providers integral to the 5G ecosystem.
- Weaponise data through interception, exfiltration, or manipulation of sensitive information transmitted over 5G networks.

## Operational framework

In 5G networks, the phases take on distinct characteristics due to the network's software-defined nature and virtualised components.

- *Priming phase:* Adversaries can exploit the expanded attack surface created by 5G's distributed architecture, conducting reconnaissance across multiple network slices and edge computing nodes while remaining undetected. This reconnaissance phase benefits from the dynamic reconfiguration of 5G architectures, allowing attackers to map vulnerabilities across different layers of the network.
- *Operational phase:* Hybrid threats become particularly potent in 5G environments. The high degree of network automation and interconnectivity means that successful attacks can cascade rapidly across systems and services, potentially affecting multiple critical infrastructure sectors simultaneously. This amplification effect distinguishes 5G-based hybrid threats from those targeting traditional telecommunications infrastructure. Unlike conventional IT environments, 5G’s reliance on dynamic resource allocation and ultra-low latency increases the speed at which attacks escalate, making early-stage threat detection even more critical.

Hybrid threats in the 5G era present unique challenges due to:

- *Global interconnectivity:* 5G's borderless nature expands the attack surface for hybrid adversaries, making attribution and defence increasingly complex.
- *Technological dependency:* The reliance on 5G for critical services amplifies the potential impact of disruptions, ranging from localised outages to systemic failures.
- *Evolving adversarial strategies:* Adversaries continuously adapt their tools, tactics, and procedures, leveraging emerging technologies like artificial intelligence (AI) to enhance the sophistication of their attacks. AI-driven attack automation further complicates defence strategies, allowing adversaries to adjust their methods in response to evolving security measures rapidly.

Hybrid threats in 5G demand a proactive and adaptive response beyond traditional security approaches. While robust security measures, cross-sector collaboration, and threat intelligence remain essential, the complex nature of these threats requires a more structured understanding of their components and relationships. An ontological framework provides this structure, enabling systematic analysis of threat patterns, more informed decision-making, and a deeper understanding of how different threat elements interact within the 5G ecosystem. Rather than focusing solely on threat elimination or early-stage mitigation, our framework supports a strategy centred on building technical resilience through effective risk reduction.

The ontological framework developed in this paper builds upon and synthesises key insights from established security frameworks, specifically ENISA's 5G Threat Landscape ^2^, STIX 2.1, and MITRE ATT&CK. Each framework contributes elements crucial to understanding hybrid threats:

- ENISA's security-by-design principles for 5G architectures ^3^
- STIX 2.1's standardised approach to threat intelligence sharing
- MITRE ATT&CK's comprehensive attack patterns and adversarial tactics

By integrating these complementary perspectives, this paper develops a unified ontological model that captures the full complexity of hybrid threats in 5G networks and facilitates interoperability between different cybersecurity frameworks, ensuring actionable insights for achieving system resilience through systematic risk reduction. Next, a more detailed account of the knowledge acquisition process, including how these frameworks informed the ontology's structure and design choices, follows.

## Knowledge acquisition

Our new initiative integrates insights from various Knowledge Organisation Systems (KOSes) and cybersecurity frameworks. The knowledge acquisition phase followed ontology engineering best practices to ensure interoperability, completeness, and relevance. This phase is embedded within the iterative development process of the ontology, reflected in its structure, classification system, and alignment with established security standards. The following sections outline the key sources and frameworks that informed the ontology’s design.

- **MITRE ATT&CK® Framework:** Provided a comprehensive taxonomy of tactics, techniques, and procedures (TTPs) relevant to cyber and hybrid threats. https://attack.mitre.org/
- **STIX 2.1 (Structured Threat Information eXpression):** A framework for representing and exchanging cyber threat intelligence informed the ontology's design of relationships and properties. https://oasis-open.github.io/cti-documentation/stix/intro
- **NIST Cybersecurity Framework:** Contributed to identifying key controls and their alignment with hybrid threat scenarios, especially concerning Critical Infrastructure Protection. https://www.nist.gov/cyberframework
- **ENISA Threat Landscape:** Enhanced the ontology's catalogue of 5G threats, risks, and vulnerabilities alongside its cybersecurity by design advocacy. https://www.enisa.europa.eu/publications/enisa-threat-landscape-report-for-5g-networks

By incorporating these well-established frameworks, our ontology benefits from their accumulated expertise and ensures alignment with industry standards. This structured approach enhances the ontology’s credibility and enables seamless integration with other cybersecurity tools and initiatives.

Significantly, our initiative followed ontology engineering best practices for transparency and reusability in the following ways:

- Implementation available at permanent namespace: https://purl.org/5g-hybrid-threats#
- Publicly accessible through GitHub repository ^4^: https://github.com/SecOntologyLab/5G-hybrid-threats
- Aligns with established standards (STIX 2.1, MITRE ATT&CK)

Our ontology ensures broad accessibility and compliance with recognised standards, providing a novel, practical framework for understanding and addressing hybrid threats in next-generation networks.

### Original contribution

Our ontology models hybrid threats against 5G infrastructures comprehensively. Key innovations include:

- Explicitly integrates hybrid threats in 5G infrastructure contexts.
- Implements comprehensive validation using advanced tools (SHACL, Protégé – HermiT, ELK, Pellet).
- Provides actionable insights for achieving enhanced resilience through vital risk reduction strategies specific to 5G infrastructure.

### Visualisation and analysis

We utilise STARDOG's Knowledge Graph functionality to visualise and explore the ontology's structure, including its classes, relationships, and properties. This facilitates analysis and understanding of the complex interplay between different elements within the hybr **i**d threat domain. ^iii^ This knowledge graph representation enables interactive threat modelling, supporting enhanced situational awareness and strategic decision-making. Several excerpts from our ontology’s Knowledge Graph will appear later in this paper.

### Advancing beyond existing tools

Our ontological approach offers several advantages:

- *Hybrid threat integration*: Models the intersection of cyber, physical, and socio-political threats in 5G contexts; by capturing these interdependencies, the ontology supports structured risk assessment across multiple threat domains
- *5G-specific focus*: Addresses unique aspects of 5G infrastructure and associated vulnerabilities.
- *Resilience through risk reduction*: Emphasises proactive resilience and risk analysis; rather than focusing solely on threat mitigation, this approach enhances long-term security planning by embedding resilience-based security principles.
- *Semantic integration*: Enables advanced reasoning and validation; ontology-driven semantic reasoning allows for automated consistency checking and inference-based threat detection, reinforcing adaptive cybersecurity strategies.

## Strategic positioning of resilience in the EU concerning hybrid threats

Recognising hybrid threats as a complex, multi-domain challenge has led European institutions to prioritise resilience as their primary strategic response. This shift was catalysed by Russia's actions in Ukraine in 2014, which demonstrated how conventional and unconventional tactics could be combined to exploit societal and technological vulnerabilities. The growing emphasis on resilience as a policy priority for the EU and its Member States has been extensively analysed by Bajarūnas ^5^, underscoring how hybrid threat strategies require cross-sectoral risk reduction measures rather than purely defensive postures. This shift highlights the necessity of preemptive risk mitigation strategies that enhance systemic robustness rather than relying solely on reactive security measures.

The establishment of the European Centre of Excellence for Countering Hybrid Threats (Hybrid CoE) ^iv^ in 2017 marked a crucial institutional step toward formalising resilience-based policy strategies. Rather than focusing solely on eliminating threats or preventing attacks, the European approach increasingly emphasises systemic resilience, achieved through effective risk reduction across multiple domains, including cybersecurity, critical infrastructure, social cohesion, and democratic institutions. Hybrid CoE is central to fostering cross-sector collaboration, bridging intelligence-sharing gaps, and supporting resilience-oriented security frameworks. Furthermore, it is key in shaping knowledge-driven strategies for countering hybrid threats, reinforcing the need for structured intelligence frameworks that support adaptive risk assessment.

By situating our research within this established resilience framework, we demonstrate how ontological modelling for 5G cybersecurity aligns with and extends these foundational efforts. Hybrid threats target interdependencies across technological and societal systems, requiring a formalised knowledge representation that enables threat intelligence modelling, structured risk assessment, and policy-driven security planning. Our ontological framework integrates these dimensions, ensuring a machine-readable, adaptable security model that supports policy-level analysis and operational cybersecurity implementations. Indeed, this approach bridges the gap between policy frameworks on hybrid threats and technical cybersecurity implementation, offering a scalable, structured method for strengthening resilience in 5G networks.

## The role of Hybrid CoE 

Headquartered in Helsinki, Hybrid CoE serves as a hub for research and training on hybrid threats, focusing on enhancing resilience among EU and NATO member states. Its work emphasises understanding the strategic goals of hybrid adversaries and how they exploit societal and institutional vulnerabilities. Hybrid CoE focuses on developing resilience and building capacities to counter hybrid threats through research, practical training, and exercises with cross-sector participants. It aims to strengthen alignment between private and public, civil and military, and academic sectors. By bridging these sectors, Hybrid CoE is critical in fostering information-sharing mechanisms and cross-domain situational awareness.

### 1. Historical context

The foundational documents that have supported the work of Hybrid CoE include the following:

- The "Joint Framework on Countering Hybrid Threats" (2016), developed by the European Union, is a cornerstone for the Hybrid CoE's work. ^v^ It broadly defines hybrid threats and outlines a comprehensive approach to countering them.
- NATO's "Strategic Concept" (various iterations) provides crucial context for understanding the alliance's perspective on hybrid threats and informs the Centre's research. ^vi^

The framework and the strategic concept reinforce the need for coordinated resilience measures across allied nations, emphasising multi-domain risk reduction strategies.

### 2. Modern-day context

Key publications from Hybrid CoE, in collaboration with the European Commission Joint Research Centre (EC JRC), include:

- *The Landscape of Hybrid Threats: A Conceptual Model* (2020) provides a structured framework for understanding the components and dynamics of hybrid threats ^6^.
- *Hybrid Threats: A Comprehensive Resilience Ecosystem* (2023) emphasises the importance of societal resilience and introduces the CORE model as a structured approach to countering hybrid threats ^7^.

For brevity, we will hereafter refer to these two documents collectively as the ‘JRC/CoE hybrid threats ecosystem.’

By integrating insights from these and other key sources, ^vii^ Hybrid CoE continues to advance a nuanced and evolving understanding of hybrid threats, strengthening its capacity to contribute to countering these complex challenges. Its research contributes to operational resilience strategies by informing tactical and strategic-level policy decisions. Our approach in the present paper aligns with Hybrid CoE's mission by addressing hybrid threats' technical and operational dimensions, particularly in the cyber domain. Focusing on 5G networks, our research provides a specialised perspective on how hybrid adversaries exploit technological vulnerabilities to achieve strategic objectives. Additionally, our use of ontologies and knowledge graphs supports Hybrid CoE’s emphasis on innovative methodologies for analysing and mitigating hybrid threats. By employing structured knowledge representation, our work facilitates interoperability between threat intelligence models, reinforcing Hybrid CoE’s goal of advancing data-driven security solutions.

Apart from its contributions to Hybrid CoE’s seminal publications ^6, 7^, JRC also plays a crucial role in shaping cybersecurity policy and technical frameworks, mainly through the JRC Cybersecurity Taxonomy ^8^. The taxonomy deals primarily with cybersecurity competencies across various domains, industry sectors and technologies ^viii^. Hence, our ontology does not incorporate any of its elements. However, we acknowledge its role in shaping European cybersecurity policy and its relevance in broader efforts to systematise cybersecurity intelligence.

### 3. Landscape of hybrid threats

In the first document ^6^ of the ‘JRC/CoE hybrid threats ecosystem,’ the collaborators produced a conceptual model outlining key components, domains, and phases of hybrid threats in this way:

Components

- Actors (and their strategic objectives)
- Tools applied by the actor
- Domains that are targeted
- Phases (including the types of activity observed in each phase)

Domains

- Physical Domains: Cyber, Infrastructure, Space, Military/Defense
- Cognitive Domains: Information, Intelligence, Legal, Social/Societal, Culture
- Functional/Organisational Domains: Economy, Administration, Diplomacy, Political

Phases

- priming, destabilisation, and coercion

The Landscape taxonomy is particularly relevant to understanding hybrid threats in 5G networks due to the interconnected nature of different domains. While in our case cyber forms the primary attack domain, 5G's role as critical infrastructure means hybrid threats targeting these networks cascade across multiple domains. For example, cyberattacks on 5G networks may simultaneously impact financial transactions, emergency communication systems, and industrial control networks, demonstrating the cross-domain implications of hybrid threats.

This multi-domain impact potential makes 5G networks an attractive target for hybrid threats, requiring a comprehensive ontological approach that captures these complex interdependencies. By structuring threat components within a formalised ontology, our research extends this taxonomy to provide a machine-readable model that enables systematic risk assessment and automated threat analysis.

Our research builds on this taxonomy by operationalising the cyber dimension, specifically in the context of 5G infrastructure. This approach facilitates enhanced situational awareness, allowing cybersecurity professionals to model and analyse hybrid threat scenarios more precisely.

### 4. A comprehensive resilience ecosystem

In the second document ^7^ of the ‘JRC/CoE hybrid threats ecosystem,’ the collaborators introduce the Comprehensive Resilience Ecosystem (CORE) model, a framework for understanding and strengthening societal resilience against hybrid threats. CORE embodies the interconnectedness of different sectors within a whole-of-society approach.

Key aspects of the CORE model include:

- *Interdependencies*: Recognising the interconnectedness of various sectors and the need for coordinated responses
- *Multi-layered resilience*: Advocating for resilience across individual, community, national, and international levels
- *Dynamic and adaptive aspects*: Emphasising the need for continuous monitoring, learning, and adaptation to evolving threats

Our ontological framework aligns with CORE by providing a structured model for these interdependencies. By formalising these relationships within an ontological structure, our approach enables machine-readable representations of resilience factors, allowing for enhanced risk modelling and adaptive response strategies.

## Extending the European frameworks

While these European models provide robust foundations, they often stop short of offering technical tools for implementation, particularly in the rapidly evolving 5G domain. Our ontological framework addresses these gaps by:

- *Formalising technical-strategic relationships:* Creating explicit mappings between high-level hybrid threat concepts and specific 5G technical vulnerabilities and models the cascading effects of cyber-attacks. By structuring these relationships within a machine-readable ontology, our approach enables automated reasoning over hybrid threat scenarios, supporting more effective situational awareness and decision-making.
- *Enhancing operational integration:* Aligning with established standards (STIX 2.1, MITRE ATT&CK, ENISA's threat taxonomies) while extending them to address 5G-specific challenges and providing a structured framework for threat intelligence sharing. This ensures interoperability with existing cybersecurity workflows, facilitating the exchange of structured threat intelligence across different security domains.
- *Operationalising resilience*: Transforming abstract resilience principles into concrete technical requirements for 5G systems and enabling real-time risk assessment. By embedding resilience-driven security parameters into the ontology, our model supports automated threat detection and dynamic risk mitigation strategies.

This integration provides security practitioners with actionable tools for implementing resilience through effective risk reduction in 5G networks. By bridging high-level European resilience strategies with operational cybersecurity needs, our framework ensures that resilience is not just a conceptual goal but a practical, enforceable aspect of 5G security.

## Related work

Conducting a comprehensive literature review on 5G security and ontology-based cybersecurity modelling presents a significant challenge due to the sheer volume of research in this area. A search of academic databases reveals well over 4,000 publications focusing on “5G infrastructure and security”, while more than 5,000 entries address “ontology and cybersecurity as applied to 5G infrastructure.” Given these vast numbers, it is impossible to exhaustively cover all relevant work within the scope of this Open Letter.

Recognising these limitations, we have carefully selected a representative subset of studies that align most closely with the objectives of this research—namely, advancing an ontology-driven approach to hybrid threats in 5G security. The selection process focused on works that contribute to:

- Security of 5G infrastructure
- Ontology-Based Approaches to Cybersecurity

We conclude the section with our contribution to modelling 5G networks in the context of hybrid threats. Our goal is to situate our work within key strands of existing research while highlighting the gaps our hybrid threat ontology seeks to address.

### Security of 5G infrastructure

Recent studies on 5G security have introduced a variety of approaches to mitigate threats against critical network infrastructures. Nkosi and Mathonsi ^9^ propose a hybrid security algorithm for 5G-IoT, integrating Double Q-learning and Poisson distribution theory to enhance intrusion detection and network slicing to limit attack propagation. Singh *et al*. ^10^ advocate for a defense-in-depth strategy that combines Zero Trust principles with multi-layered security, addressing threats such as rogue base stations and DDoS attacks. Kholidy *et al*. ^11^ explore a hybrid machine learning (ML) approach, leveraging Deep Extra-Trees (DET) classifiers to detect 5G-IoT cyberattacks. Suomalainen *et al*. ^12^ analyse public safety communications in hybrid 5G networks, identifying security risks in spectrum sharing, Advanced Persistent Threats (APTs), and network slicing. Sun *et al*. ^13^ propose an automated attack and defense framework using hierarchical security models and AI-driven threat analysis. Odarchenko *et al*. ^14^ introduce security Key Performance Indicators (KPIs) for 5G network slicing, focusing on risk assessment and continuous security monitoring, while Serrano ^15^ proposes a blockchain-integrated neural network model for decentralised authentication in 5G-enabled IoT smart city environments. Lastly, Oruma *et al*. ^16^ focus on security risks associated with 5G-enabled social robots in public spaces, emphasising cybersecurity, physical security, and supply chain vulnerabilities. These diverse approaches highlight the growing need for adaptive, multi-layered security frameworks tailored to the dynamic and interconnected nature of 5G networks.

Earlier studies also contributed significantly to understanding 5G security challenges. Ahmad *et al*. ^17^ broadly assess security threats across 5G network components, aligning solutions with ITU-T and 3GPP standards. Fang *et al*. ^18^ introduce a flexible authentication and identity management system for 5G, proposing cryptographic techniques and physical layer security mechanisms. Rupprecht *et al*. ^19^ systematise security research across multiple mobile network generations, identifying fundamental weaknesses and open research questions for future 5G security enhancements. Carvalho *et al*. ^20^ explore agile security as a dynamic, real-time approach to securing 5G networks, proposing a risk-aware edge orchestrator for Mobile Edge Computing (MEC) and Network Functions Virtualisation (NFV). These works focus on shifting from traditional mobile networks to 5G, advocating for security-by-design principles and resilience-focused frameworks that proactively address emerging threats.

### Our contribution

While these studies provide valuable insights into 5G security, they primarily focus on cybersecurity threats, risk-aware monitoring, access control, and attack detection techniques. In contrast, our work introduces an ontological framework tailored to hybrid threats—attacks that combine cyber, physical, and socio-political dimensions in line with established security frameworks and standards already identified (ENISA’s 5G Threat Landscape, STIX 2.1, and MITRE ATT&CK) and including the ‘JRC/CoE hybrid threats ecosystem’. By formalising these hybrid threat interactions within a machine-readable ontology, our approach enables structured threat intelligence modelling and cross-sector security planning.

Unlike performance-based security monitoring, blockchain-driven authentication, or algorithmic survey-based models, our ontology provides a structured representation of hybrid threats, capturing interdependencies between adversarial tactics, techniques, and affected assets while facilitating interoperability, adaptive risk assessment, and knowledge-driven mitigation strategies. This comprehensive approach ensures security practitioners can systematically analyse hybrid threat scenarios, improving situational awareness and proactive defence mechanisms.

This shift from isolated threat detection and security metrics monitoring to an extensible, knowledge-driven framework enhances 5G security’s strategic resilience and improves cross-domain threat intelligence interoperability. By integrating multi-layered risk assessment with formalised knowledge representation, our approach strengthens the foundation for resilience-driven security architectures in 5G networks.

### Ontology-based approaches to 5G security

In their ‘work-in-progress’ undertaking, Bernardini *et al*. ^21^ conducted an ongoing assessment of 5G security ontologies, highlighting the limited scope of existing work. They classify previous efforts into three main categories: *5G architecture, 5G cybersecurity, and 5G ontologies*. They reveal that most studies focus on network slicing, virtualisation, and service orchestration while neglecting security modelling beyond isolated technical concerns. Their review identifies significant gaps in hybrid threat representation, emphasising that existing 5G security ontologies lack multi-layered risk assessment and resilience-based modelling. This limitation underscores the need for a more comprehensive approach capable of integrating cyber, physical, and socio-political dimensions into 5G security ontologies.

To address these gaps, Bernardini *et al*. propose an ontological framework aligned with 3GPP Release 17, structured to support end-to-end security assessments across different layers of 5G infrastructure. However, their approach is still in its early stages, lacking iterative validation mechanisms and real-world security modelling beyond 5G service-based concerns. While their work provides a valuable starting point for formalising security in 5G networks, it does not fully capture the cross-domain nature of hybrid threats that integrate cyber, physical, and socio-political attack vectors. The absence of an adaptive risk assessment mechanism further limits its applicability in dynamically evolving threat landscapes.

Rivadeneira and Gómez ^22^ provide a comprehensive systematic review of cybersecurity ontologies, categorising existing work into four primary areas: *general cybersecurity ontologies, networking-focused cybersecurity ontologies, software security ontologies, and human-factor cybersecurity ontologies*. General cybersecurity ontologies consist of broad taxonomies that classify cyber threats, vulnerabilities, and security measures at a conceptual level. Networking-focused cybersecurity ontologies emphasise network security, intrusion detection, and protocol-based risk assessment, while software security ontologies model software vulnerabilities, access control mechanisms, and malware detection. Lastly, human-factor cybersecurity ontologies address risks related to social engineering attacks, misinformation, and decision-making biases in cybersecurity.

Despite the breadth of research, these authors identify several critical limitations in cybersecurity ontologies. One key shortcoming is the lack of dynamic threat modelling, as most ontologies function as static classification systems rather than adaptive frameworks capable of evolving with emerging cyber threats. Additionally, there is limited cross-domain integration, with most ontologies addressing only technical risks while overlooking socio-political and physical security factors, essential components of modern cybersecurity threats. Furthermore, few studies explicitly focus on resilience and risk reduction—crucial factors for maintaining long-term security, particularly in complex infrastructures like 5G networks. Their review highlights the need for more comprehensive and adaptable ontological frameworks to bridge these gaps and support the dynamic and multi-layered nature of cybersecurity challenges. This highlights the need for an ontology-driven approach that classifies threats and facilitates structured risk intelligence, enabling proactive mitigation strategies.

Rivadeneira and Gómez's findings complement Bernardini *et al*.’s assessment, reinforcing the conclusion that cybersecurity ontologies—particularly in the 5G domain—remain limited in their ability to model hybrid threats and multi-layered risk scenarios. While both studies highlight progress in security ontologies, they also expose persistent gaps in cross-domain threat representation, resilience-focused modelling, and adaptability to evolving attack landscapes. This underscores the need for a more integrated approach that moves beyond static classifications to support structured risk intelligence, hybrid attack modelling, and dynamic security assessments. Addressing these gaps requires an integrated ontological framework that moves beyond static classifications, supporting real-time risk assessment, hybrid attack modelling, and cross-domain security analysis.

### Further studies on ontology-based approaches to 5G security

In their work on ontology-based intrusion detection, Bisht *et al*. ^23^ propose OIDS, a security framework for identifying intrusions in 5G wireless sensor networks (WSNs). Their approach models sensor node interactions, patrol node behaviour, and attack classifications, leveraging ontology to detect anomalies and security breaches. The system evaluates attacks based on live packet monitoring and hierarchical security modelling, improving attack detection accuracy while maintaining low computational overhead. By integrating semantic reasoning, OIDS enhances the adaptability of intrusion detection mechanisms in resource-constrained 5G environments.

Randles *et al*. ^24^ introduce an ontology-driven closed control loop framework for autonomic 5G network management, using semantic graphs to monitor, validate, and maintain intent-based network operations. Their Intent-Based Control Loop Ontology (IBCLO) ensures that network service goals—such as Quality of Service (QoS) in 5G slices—are dynamically upheld via machine-driven reasoning and automated corrective actions. This approach aligns with the broader trend of intent-based networking, where automated policies adjust network behaviour in response to real-time security and performance metrics.

Salazar *et al*. ^25^ present a mutation-based ontology for cybersecurity testing, focusing on 5G network protocols. Their ontology structures network protocol elements, mutation operators, and traffic flow patterns to facilitate automated fuzz testing for security vulnerabilities. Implemented within the 5Greplay tool, their ontology simulates scenarios such as NAS replay attacks, malformed NGAP packet injections, and IoT traffic encapsulation. This demonstrates the potential of ontology-driven frameworks in automating security validation processes, reducing manual intervention in protocol vulnerability assessments.

Mozzaquatro *et al*. ^26^ propose an ontology-based cybersecurity framework for IoT security, called IoTSec, which classifies threats, vulnerabilities, and security services, allowing for dynamic security service provisioning. The framework integrates a model-driven security design phase with real-time security monitoring, leveraging semantic reasoning to automate security decisions. This adaptive approach enables IoT systems to dynamically adjust security measures in response to evolving threat landscapes.

Mahmud *et al*. ^27^ examine the role of software-defined networking (SDN) security in tactical military environments, proposing an ontology for managing SDN-based network policies, resource allocation, and interoperability. The study emphasises security vulnerabilities in SDN controllers and suggests resilient multi-controller architectures for secure battlefield communications. By formalising SDN security concepts within an ontology, this work enhances policy-driven security enforcement in dynamic and mission-critical environments.

Globa *et al*. ^28^ propose an ontology for data processing optimisation in telecommunications networks, focusing on network scalability, workload management, and energy efficiency. Their model consists of three layers—processing system components, workload classification, and quality assessment metrics—designed to enhance telecom resource allocation. This structured representation improves the efficiency of network resource distribution, aligning with broader efforts to optimise performance in large-scale 5G deployments.

### Our contribution: toward a hybrid threat ontology for 5G security

Building on the insights from Bernardini *et al*. and Rivadeneira & Gómez, our work introduces a hybrid threat ontology that addresses several critical gaps identified in these assessments. Unlike existing 5G security ontologies, which primarily focus on network security, service-layer protection, and individual threat categories, our approach integrates multi-domain threat intelligence to model hybrid attacks that span cyber, physical, and socio-political dimensions. Furthermore, whereas many prior ontologies adopt static taxonomies, our framework enables dynamic risk assessment and adaptive threat modelling, supporting structured mitigation strategies and resilience planning. This adaptability ensures that hybrid threats can be assessed in real time, accommodating evolving adversarial tactics and newly emerging attack vectors.

In contrast to Bernardini *et al*.’s early stage 5G security ontology, our work incorporates iterative validation techniques, including SHACL testing and real-world case studies, to refine its practical applicability for cybersecurity operations. This ensures that our ontology is theoretically robust and capable of real-world deployment within evolving 5G security landscapes. Moreover, while Rivadeneira & Gómez classify cybersecurity ontologies into discrete technical and human-centred domains, our hybrid threat ontology transcends these divisions by incorporating interdependent attack vectors, adversarial behaviours, and cascading effects that cut across multiple threat categories. By bridging these classifications, our approach provides a more comprehensive and context-aware understanding of hybrid threats.

By synthesising knowledge from existing cybersecurity ontologies while addressing their structural limitations, our work offers a novel contribution to the field of 5G security modelling. It extends beyond traditional cyber risk frameworks by emphasising structured hybrid threat intelligence, dynamic resilience strategies, and compliance with recognised cybersecurity standards, including ENISA’s 5G Threat Landscape, MITRE ATT&CK, and STIX. Additionally, it aligns with the ‘JRC/CoE hybrid threats ecosystem’, integrating multi-domain threat modelling approaches to capture the interplay between cyber, physical, and socio-political attack vectors. This integration facilitates cross-sector security coordination, allowing cybersecurity professionals to align their threat modelling efforts with broader resilience-driven strategies.

Unlike prior ontologies that focus on specific aspects of cybersecurity, such as intrusion detection, service automation, or fuzz testing, our work introduces a comprehensive hybrid threat ontology that integrates cyber, physical, and socio-political attack dimensions within 5G security modelling. While studies like Randles *et al*. and Salazar *et al*. emphasise network automation and security testing, our ontology moves beyond protocol-layer risks to address structured threat intelligence and adversary behaviour modelling. Similarly, works like Bisht *et al*. and Mozzaquatro *et al*., which focus on anomaly detection within sensor networks and IoT security, remain limited to isolated threat categories. In contrast, our approach is designed to holistically assess hybrid attack scenarios across the broader 5G infrastructure ecosystem.

In contrast to Mahmud *et al*., whose SDN-based security ontology is tailored to mission-critical tactical networks, our work provides a generalisable framework for hybrid threat intelligence that is not restricted to a specific network architecture or operational environment. Likewise, while Globa *et al*. explore efficiency and scalability in telecom networks, their ontology lacks structured security intelligence and adversarial modelling, which are central to our approach. Our framework, in contrast, is designed to accommodate evolving threat scenarios and resilience-based mitigation strategies within diverse 5G infrastructure applications.

By bridging network security ontologies, software security ontologies, and human-centred risk models, our hybrid threat ontology provides a resilience-driven approach that extends beyond threat detection to strategic threat mitigation and risk-informed decision-making in 5G infrastructures. This positions our work as a necessary evolution of cybersecurity ontologies, addressing the interconnected nature of modern hybrid threats while enabling real-world security applications and compliance-driven risk management strategies.

## Design rationale and competing approaches

The development of the 5G Hybrid Threat Ontology involved deliberate technical choices that distinguish it from existing cybersecurity ontologies. This section articulates key design decisions in contrast to alternative approaches.

### Knowledge representation and validation architecture

We selected OWL 2 DL over lighter-weight alternatives (RDFS, property graphs) because hybrid threat modelling requires the expressivity of description logic for complex relationship reasoning – specifically, property chains for modelling threat actor collaboration patterns and qualified cardinality restrictions for group membership requirements. This contrasts with Bernardini *et al*.'s framework, which relies primarily on taxonomic classification without semantic reasoning capabilities, and Salazar *et al*.'s mutation-based ontology, which uses RDF optimised for protocol testing rather than cross-domain risk assessment.

Our dual-layer validation approach, combining OWL axioms with separate SHACL constraints, differs fundamentally from Randles *et al*.'s IBCLO framework, which embeds validation logic within class definitions. By separating ontological structure from constraint validation through Python scripting, we enabled controlled iteration – refining validation rules without modifying the ontology structure itself. This architectural separation proved essential for the phased development methodology, allowing rapid refinement during the WIRED case study integration without structural modifications.

### Ontological structure and framework integration

The choice of ThreatConcept as the root class, rather than directly adopting the STIX vocabulary, reflects the hybrid nature of threats across multiple domains. While STIX 2.1 excels at cyber threat intelligence exchange, it lacks explicit constructs for socio-political dimensions like 5G-enabled disinformation campaigns. Our federated alignment approach – maintaining distinct STIX, ENISA, MITRE ATT&CK, and NIST vocabularies through custom annotation properties – preserves the semantic intent of each framework while enabling cross-framework reasoning. This differs from Mahmud *et al*.'s SDN security ontology, which standardises on domain-specific vocabularies, limiting cross-sector applicability.

The three-branch structure (NefariousActivity, ThreatActor, AttackPattern) emerged from SHACL validation iterations that revealed modelling conflicts when representing attack instances versus attack methodologies. Separating NefariousActivity as a distinct branch-maintained alignment with ENISA's activity-based taxonomy while resolving these conflicts – a challenge Bernardini *et al*.'s architecture-centric model does not address.

### Expressivity enhancement through iterative development

The WIRED case study revealed limitations in modelling complex APT group coordination using 5G amplification mechanisms, driving systematic DL expressivity enhancements from ALHI(O) to ALCHFRSIOQ(D). These additions – including disjoint classes for actor type exclusivity, property chains for collaboration inference, and transitive properties for group hierarchies – directly support the case study's requirement to model multi-actor disinformation campaigns exploiting 5G infrastructure. Detailed expressivity analysis and ontology metrics documenting this evolution are available in our GitHub repository (SecOntologyLab/5G-hybrid-threats).

By explicitly addressing design alternatives, we demonstrate that the ontological framework emerged from systematic evaluation rather than arbitrary choices, directly supporting resilience through structured risk reduction in hybrid threat scenarios.

## Ontology development

The ontology development process was rooted in established semantic best practices and industry standards to ensure clarity, consistency, and interoperability. Using the Web Ontology Language (OWL) to define classes and properties, the ontology employs the concise and readable Resource Description Framework (RDF) in Turtle format for representation, ensuring both formal expressivity and machine-readability.

Key aspects of the development process included:

- *Hierarchical structure*: The ontology was designed as hierarchical, facilitating efficient knowledge organisation and reasoning.
- *Standardised terminology*: PascalCase naming conventions and singular class names were adopted for consistency and clarity, aligning with ontology engineering best practices.
- *Reusability*: Existing terms and concepts were reused from relevant cybersecurity resources to promote interoperability and avoid redundancy.
- *Extensibility*: The ontology was designed to be extensible, allowing the incorporation of new threat information and evolving attack patterns.

Significantly, the ENISA taxonomy, the STIX 2.1 standard, and the MITRE ATT&CK framework primarily served as valuable knowledge bases, informing the structure and content of the ontology. These frameworks provided a standardised foundation for classifying and modelling hybrid threats, ensuring alignment with established cybersecurity practices.

## Overview of the developmental process

The development of the 5G ontology unfolded through a dynamic and iterative process, reflecting the ‘toward’ aspect emphasised in the paper's title. This encompassed four distinct phases, each contributing to the ontology's robustness and responsiveness to evolving threats.

**Phase 1** laid the groundwork by integrating elements from ENISA's 5G cybersecurity framework and the ‘JRC/CoE hybrid threats ecosystem’. This phase established a basic ontological structure for representing 5G vulnerabilities and incorporated preliminary validation procedures.
**Phase 2** enhanced the framework by integrating STIX elements and the ENISA taxonomy. SHACL validation was implemented to ensure data integrity and consistency. This phase also involved iterative improvement based on validation results and refinement of the relationships between threats, actors, and vulnerabilities.
**Phase 3** delved into the technical implementation and validation of the ontology. Further constraint validation ensured adherence to predefined rules. The Protégé development environment was utilised, and validation was conducted using multiple reasoners (HermiT, ELK, Pellet) to verify logical consistency and enhance semantic relationships.
**Phase 4** focused on the practical application and validation of the ontology through a case study examining hybrid threats to election infrastructure. The study explored multiple dimensions of interference, including disinformation campaigns, targeted attacks on election systems, and the role of APT42 as an emerging threat actor. By mapping these threats within the ontology, the study demonstrated how 5G-enabled infrastructure can be exploited to amplify disinformation and disrupt electoral processes.
This phase also revealed structural gaps in the ontology, leading to key enhancements. The APT42 class was formally incorporated, alongside various supporting entities such as Campaign and ElectionInfrastructure, refining the representation of adversarial tactics and their impact on critical systems. These additions expanded the ontology’s DL expressivity, improving its ability to capture complex hybrid threat relationships.
Finally, reasoning validation and cross-framework alignment confirmed that the ontology maintains conceptual consistency, interoperability with standardised threat intelligence models, and compliance with established security frameworks. These refinements reinforce its applicability in real-world cybersecurity operations, supporting structured intelligence modelling and resilience-focused risk reduction.

### Opening up new opportunities

This phased development process represents significant progress towards a comprehensive ontological framework for addressing hybrid threats in 5G environments. While focusing on achieving resilience through risk reduction, the framework remains deliberately dynamic, ready to evolve with emerging threats and security requirements. By presenting this as a ‘new initiative’, in the style of an Open Letter, we open opportunities for:

- Enhanced threat modelling capabilities
- Improved integration of security frameworks
- Advanced validation methodologies
- Practical application to emerging threats

The framework's evolution continues, guided by the dual objectives of resilience and risk reduction while maintaining the flexibility needed to address the dynamic nature of hybrid threats to 5G infrastructure.

## The ontology development process

To better understand the evolution of the ontology, we examine each phase in more depth, tracing the iterative process of development and refinement.

### Phase 1

In this first phase, we developed a conceptual ontology to capture hybrid threat scenarios, focusing on APT29, a state-linked cyber threat actor, to structure historical data to support a strategy for achieving resilience and reducing risk. Our methodological approach involved multiple iterative steps, beginning with an initial, minimal ontology and progressing towards a more robust yet still low-level structure. The figure below situates APT29 as a sub-class of Advanced Persistent Threat (APT).

This initial phase produced an early-stage ontology for modelling hybrid threats. The outcomes are summarised below:

*Ontology Metrics*

- Classes: 25, representing APT groups, malware types, assets, and cyber operations
- Properties: 15, including relationships such as usedByAPT, targets, and techniquesUsed
- Instances: 10, including specific cyberattacks like Solar Winds supply Chain Attack, an instance of APT29Activity and WannaCry, an instance of Malware.

**Figure 1.**
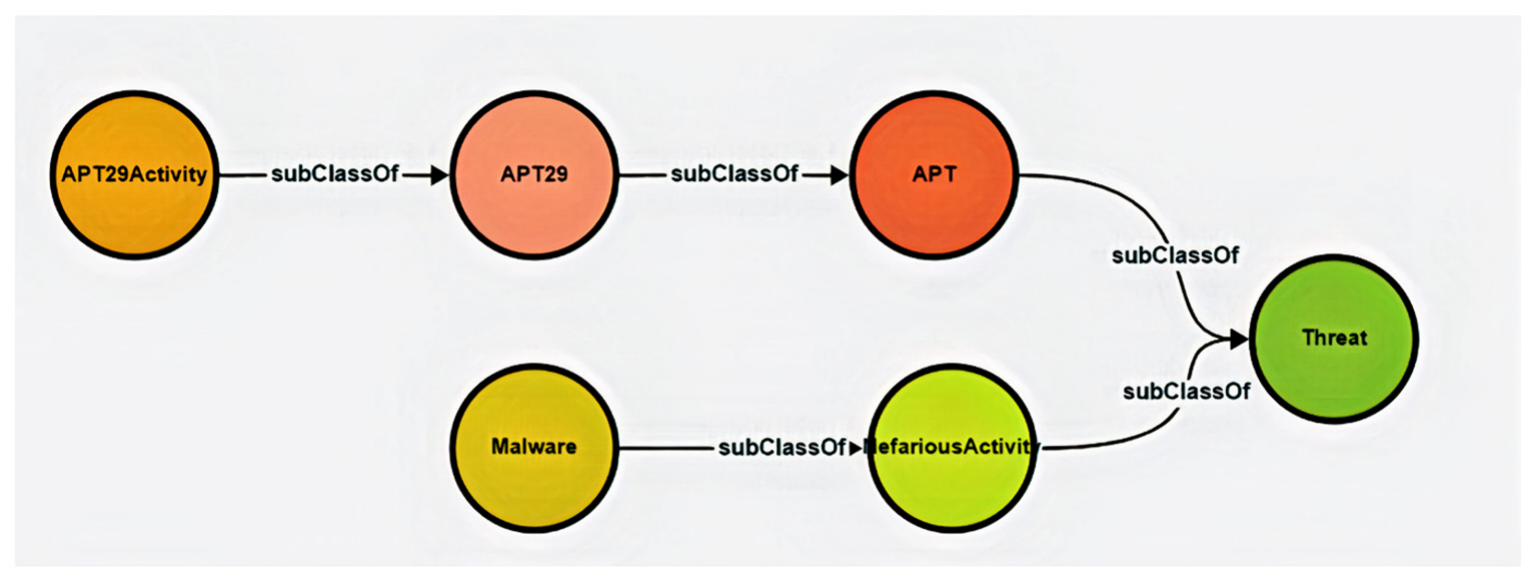
Early-stage ontology depicting relationships between threat-related concepts. This visualisation captures an early-stage representation of the 5G Hybrid Threat Ontology, structured to classify adversarial entities and activities. The diagram highlights APT29 as a subclass of the broader Advanced Persistent Threat (APT) category, while NefariousActivity is shown as a subclass of Threat, aligning with the ENISA 5G Taxonomy. These structured relationships facilitate semantic reasoning and integration with standardised cybersecurity frameworks. By structuring threats hierarchically, this ontology ensures alignment with industry-recognised taxonomies while providing a flexible foundation for expanded hybrid threat modelling.

Figure 1 depicts the major components of early-stage ontology and illustrates key hierarchical relationships between them.

### Phase 2

The next step in developing our 5G ontology for countering hybrid threats followed a systematic approach that integrated two essential frameworks: STIX 2.1 and the ENISA 5G Threat Taxonomy. We evaluated STIX categories for their applicability to 5G threat modelling while analysing ENISA's 5G Taxonomy to ensure alignment with established security frameworks. This dual consideration was crucial for maintaining compatibility with existing threat intelligence platforms while addressing the unique aspects of 5G infrastructure security.

The foundation of our working ontology began with carefully selecting a root concept. We aligned with STIX's ThreatConcept while maintaining explicit connections to ENISA's taxonomy through custom annotation properties. This approach is exemplified in our root class definition as represented by Turtle in the following snippet:


# Section 2: Root Level Concepts
fght:ThreatConcept a owl:Class ;
  rdfs:label "ThreatConcept" ;
  rdfs:comment """Root class representing hybrid threats in 5G infrastructure, aligned with
      STIX (ThreatConcept) and ENISA taxonomies (Threat) """;
  fght:alignedWithStandard "STIX 2.1" ;
  fght:alignedWithENISA "ENISA Threat Taxonomy 2020" ;
  fght:externalReference <https://www.enisa.europa.eu/publications/enisa-threat-landscape-report-for-5g-networks>. 


This code from the 5G Hybrid Threat Ontology represents the ThreatConcept class, a root-level concept structured using Semantic Web technologies. This class is aligned with both STIX 2.1 (Structured Threat Information eXpression) and ENISA's 5G Threat Taxonomy, ensuring compatibility with established cybersecurity standards. The rdfs:comment annotation provides contextual information about its role in representing hybrid threats within 5G infrastructure, emphasising its relevance to multi-domain security modelling. Additionally, the fght:externalReference property links directly to the ENISA 5G Threat Landscape Report, integrating authoritative cybersecurity resources into the ontology. This approach enhances interoperability while maintaining a structured, machine-readable format for threat intelligence representation. Significantly, the ontology now adopts the prefix 'fght:' (short for '5G hybrid threats') instead of the generic 'ex:' to enhance clarity and align with best practices in ontology engineering.

**Figure 2.**
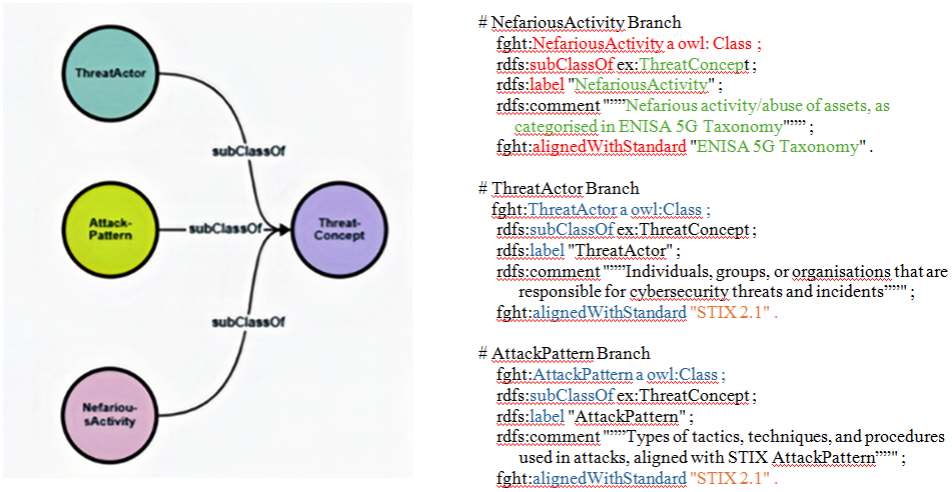
Partial view of the early-stage ontology. This is a partial view of the early-stage ontology, illustrating the hierarchical relationships between key threat concepts. The ThreatConcept root class branches into three primary categories: NefariousActivity, ThreatActor, and AttackPattern. As revealed in the three code snippets, this structure ensures alignment with both STIX 2.1 and ENISA taxonomies while providing a foundation for hybrid threat modelling in 5G security.

We developed three primary branches from this foundation: NefariousActivity, ThreatActor and AttackPattern. The latter two categories are aligned with STIX, while the NefariousActivity branch corresponds to ENISA's taxonomy, as demonstrated in the tree structure of Figure 2:

### Hierarchy and metrics

**Figure 3.**
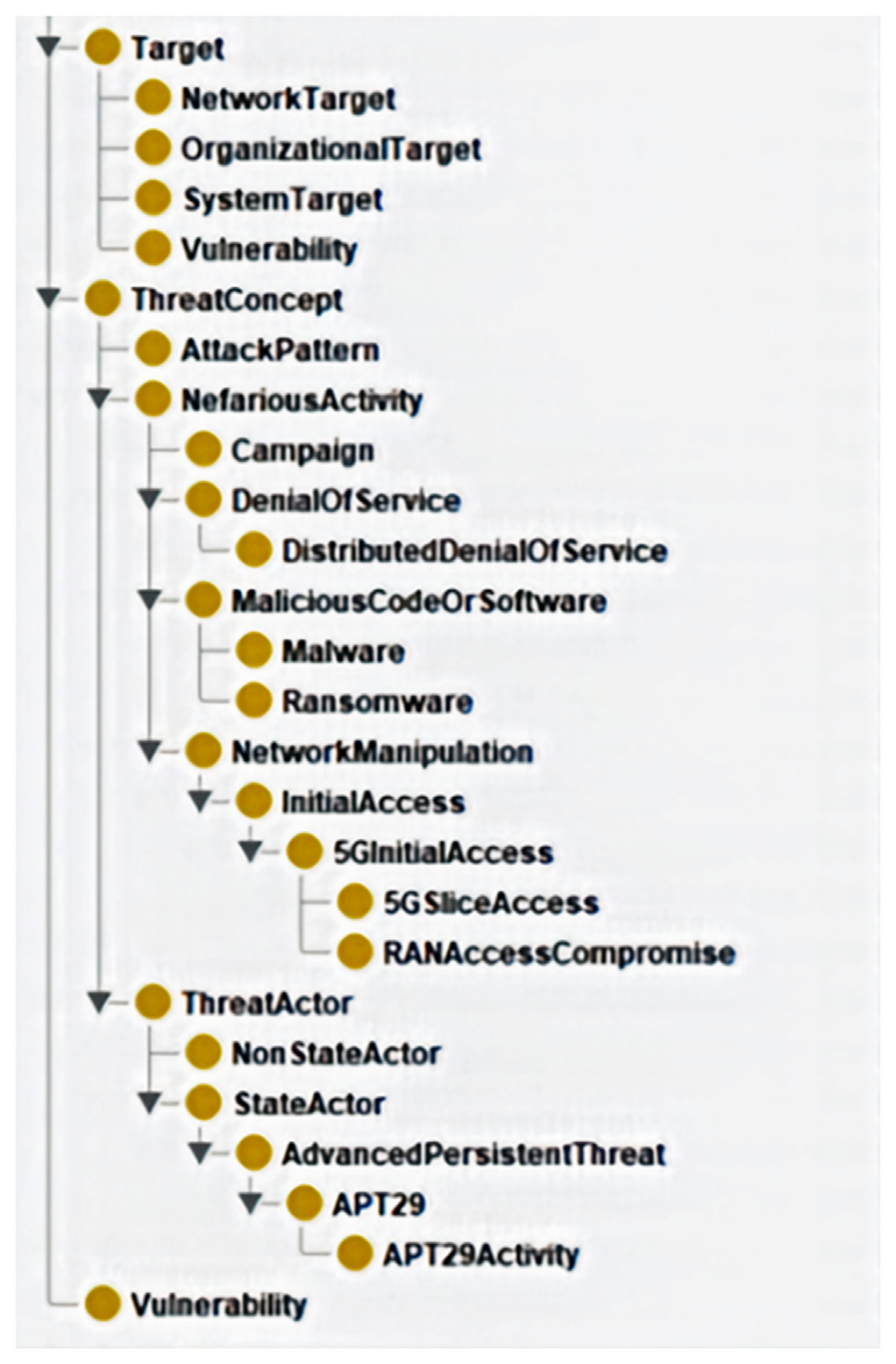
Class hierarchy at the end of Phase 2. The ontology metrics can be examined in the GitHub supplementary materials in the docs/Metrics folder. These have been taken from the Protégé tool, provide a quantitative assessment of key components, such as total axioms, classes, properties, individuals, assertions, and logical constraints. Figure 3 illustrates the ontology’s complexity, reasoning capabilities, and adherence to cybersecurity frameworks, ensuring alignment with structured threat intelligence standards.

The class hierarchy and ontology metrics presented in the two visuals below reflect the state of the ontology at the end of Phase 2. The hierarchy in Figure 3, depicted by the Protégé tool, captures key structural components, including threat concepts, attack patterns, actors, and vulnerabilities. These elements form the foundation for modelling hybrid threats in 5G infrastructure.

### SHACL validation

To maintain semantic accuracy and structural integrity, SHACL (Shapes Constraint Language) was employed as a validation mechanism throughout Phase 2. SHACL constraints were defined to enforce logical consistency, ensuring that entity relationships—such as those between threats, actors, and attack patterns—conformed to expected modelling rules. This validation process was instrumental in refining class-property interactions, identifying structural inconsistencies, and enhancing the overall reliability of the ontology.

SHACL validation runs were conducted in Visual Studio Code (VS Code) using a Python script. The validation process relied on a two-file system: one containing the ontology itself and another containing the SHACL constraint definitions. ^ix^ This approach facilitated efficient constraint checking, systematic debugging, and controlled iteration throughout development. Additionally, it provided a robust mechanism for validating updates while preserving the integrity of the ontology.

Integrating SHACL validation into the development workflow ensured compliance with STIX and ENISA standards while maintaining flexibility for future expansions.

### Validation results

Our key findings from the validation process, identified structural inconsistencies, refined constraints, and critical lessons learned. The results highlight challenges encountered and methodological breakthroughs in strengthening the ontology’s expressivity and resilience against inconsistencies. These are summarised below.

### Initial challenges

Early SHACL validation attempts exposed several structural issues, including:

- Property constraint violations, particularly in threat-actor relationships
- Hierarchical conflicts between NefariousActivity and MaliciousCodeOrSoftware branches
- Ambiguous positioning of the Target concept within the hierarchy
- Unclear boundaries between malware instances and general nefarious activities

### Methodological breakthrough

The validation followed an incremental refinement approach, initially simplifying the ontology and SHACL constraints to eliminate errors and then progressively expanding the class hierarchy while maintaining structural integrity. This stepwise methodology enabled us to:

- Establish a foundational, error-free structure before introducing greater complexity
- Ensure that SHACL constraints remained valid as new classes and relationships were added
- Ensured correct instance definitions for distinct threat categories (e.g., Malware, Ransomware, APT29 activities) by systematically addressing Focus Node-related errors, leading to improved SHACL validation and structural consistency
- Maintain hierarchical integrity across the ontology while integrating new cybersecurity frameworks

### Validation framework

We developed comprehensive SHACL shapes to ensure:

- Structural integrity at the class level
- Data quality at the instance level
- Proper documentation of cyber activities
- Consistency with STIX and ENISA frameworks

### Key achievements

- Successful resolution of DirectNefariousActivityShape validation
- Proper positioning of Target as a root-level concept
- Enhanced the representation of 5G-specific threat vectors – NefariousActivity, NetworkManipulation
- Reclassified InitialAccess from subclass of AttackPattern to NetworkManipulation, improving alignment with real-world threat scenarios

### Validation results

The refined ontology demonstrated:

- Successful integration with STIX threat intelligence standards
- Alignment with ENISA's 5G security framework
- Clear hierarchical relationships, improving semantic reasoning
- Flexibility for future extensions, allowing adaptation to emerging threats

Implementing our developmental approach resulted in an ontological framework that effectively bridges STIX threat intelligence standards, ENISA’s 5G security framework, and key constructs from the ‘JRC/CoE hybrid threats ecosystem.’ This phase established a structured yet adaptable ontology that maintains clear hierarchical relationships while ensuring the flexibility to accommodate evolving threat landscapes and future extensions. By integrating these diverse frameworks, the ontology enhances interoperability, supports structured threat intelligence, and lays a strong foundation for resilience-focused security modelling in 5G environments.

### Phase 3

***Advancing ontology validation and refinement***

With the foundational structure established and SHACL validation confirming key constraints, Phase 3 focused on enhancing the ontology’s reasoning capabilities and ensuring its logical soundness. The transition from Phase 2 to Phase 3 marked a shift from structural validation to logical consistency and inference testing. This ensured the ontology could support structured risk assessment, resilience planning, and cross-domain threat intelligence integration.

A key aspect of this phase was the transition to the Protégé development environment, which facilitated reasoning validation using multiple reasoners. This iterative refinement strengthened the ontology’s logical structure, ensuring threat relationships, hierarchical classifications, and risk factor associations adhered to formal ontological principles and cybersecurity frameworks.

By systematically refining the ontology through reasoner-based validation, we ensured that its logical structure remained coherent, free from inconsistencies, and aligned with established security standards. This process involved verifying the correctness of class hierarchies, ensuring the proper application of object and data properties, and confirming that inferred relationships accurately represented hybrid threats within a resilience-driven security framework.

This phase represented a significant step forward in making the ontology technically robust and practically applicable for resilience-focused security modelling. By integrating formal reasoning mechanisms, Phase 3 validated the ontology’s ability to support structured threat analysis, enable cross-domain security assessments, and facilitate intelligence-sharing in hybrid threat contexts. We now turn to the specific results obtained from our reasoning tests, highlighting their implications for the continued refinement and scalability of the 5G Hybrid Threat Ontology.

***Technical details: reasoning***

To ensure the ontology’s logical consistency and semantic coherence, we conducted reasoning tests using HermiT 1.4.3.456, ELK 0.6.0, and Pellet 2.2.0, all of which are available on the Protégé platform. These evaluations helped determine the ontology’s capacity to support structured intelligence modelling, resilience-driven security planning, and cross-sectoral cybersecurity knowledge integration.

The results of these tests are reported in Table 1, demonstrating how the ontology maintains logical consistency, effectively models hierarchical relationships, and ensures compliance with cybersecurity ontologies and standards. These evaluations confirmed that the ontology’s formal structure could accurately model adversarial tactics, system vulnerabilities, and hybrid threat interdependencies in a structured and semantically rigorous manner.

**Table 1.** Reasoner performance comparison. The table presents the reasoning performance results, comparing processing time, loading time, and inference capabilities across the three reasoners.

Metric	HermIT	ELK	Pellet
Processing Time	185 ms	360 ms	134 ms
Loading Time	376 ms	288 ms	258 ms
Warning/Issues	None reported	Multiple incompleteness warnings	None reported
Inferences Computed	All standard	Limited by EL++ restrictions	All standard

The results demonstrated robust performance across multiple reasoning approaches, with Pellet showing the fastest processing time at 134 milliseconds, followed by HermiT at 185 milliseconds and ELK at 360 milliseconds. While both HermiT and Pellet completed all standard inference tasks without issues, ELK produced multiple incompleteness warnings due to our ontology's use of constructs beyond EL++ expressivity. These included 34 data property assertions, 8 data properties, and 5 anonymous individuals.

All three reasoners successfully processed the core inference types, including class hierarchy, property hierarchies, and assertions. The loading efficiency varied among reasoners, with Pellet demonstrating the most efficient ontology loading time at 258 milliseconds, followed by ELK at 288 milliseconds and HermiT at 376 milliseconds.

These results validate the logical consistency of our ontological framework and its practical implementation ability while highlighting the trade-offs between different reasoning approaches in handling our hybrid threat modelling constructs.

***Structure of the preliminary ontology***

***Implications and next steps***

**Figure 4.**
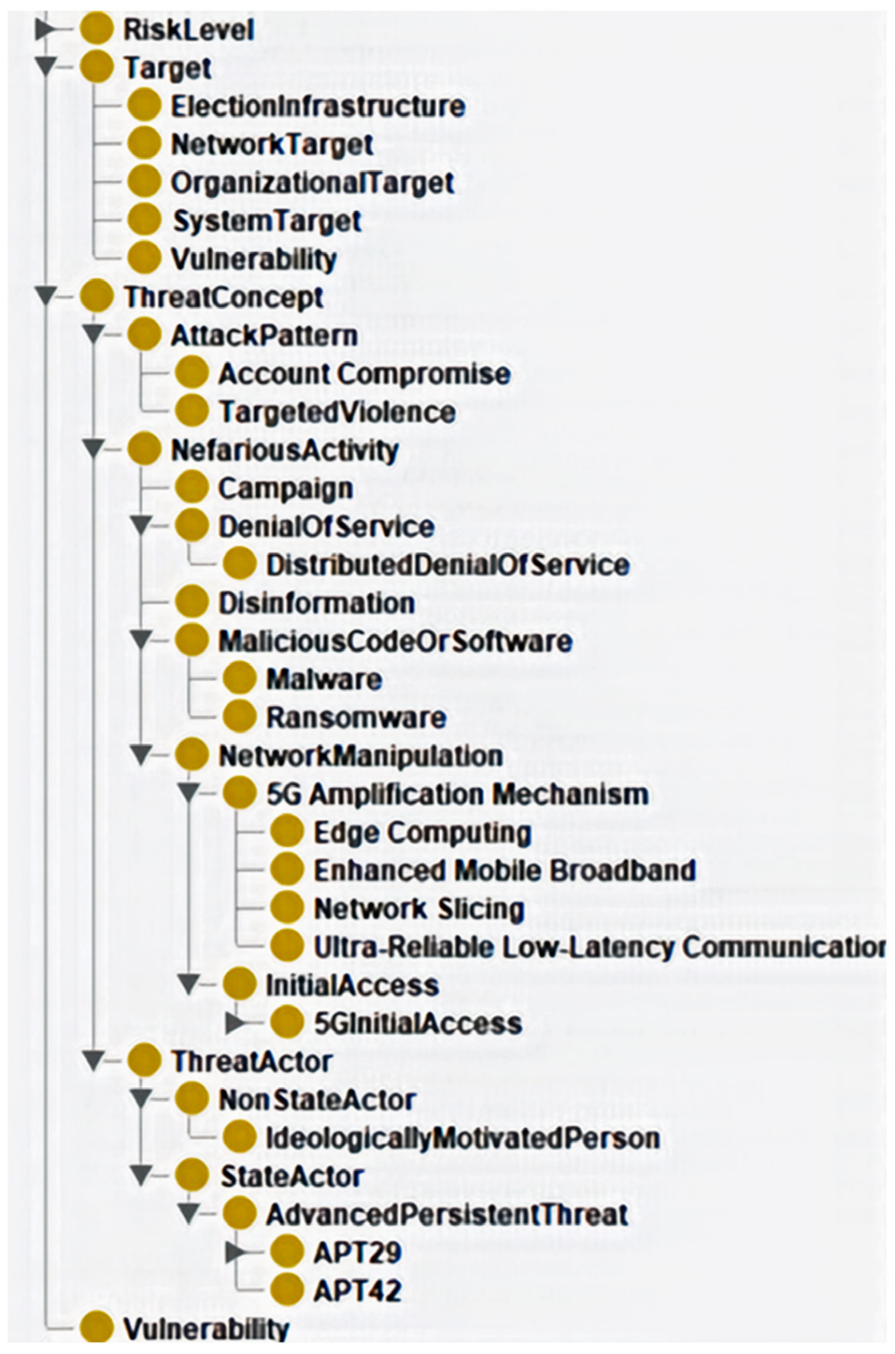
Class hierarchy of preliminary ontology. Figure 4 presents the class hierarchy, highlighting the structured representation of hybrid threats, attack patterns, threat actors, and vulnerabilities. The hierarchical structure ensures a precise classification of key cybersecurity elements. At the same time, the ontology metrics outlined in the GitHub supplementary materials, located in the docs/Metrics folder, reflect a well-balanced framework with a manageable level of complexity, supporting both expressivity and maintainability, which in turn supports the goal of achieving resilience through effective risk reduction.

The validation results and reasoner performance comparison for Phase 3, as well as the structure analysis for Figure 4, confirmed the ontology's readiness as a preliminary framework for modelling hybrid threats in 5G contexts. The ontology's strengths lie in its comprehensive coverage of the threat landscape, efficient constraint checking, and logical consistency.

These refinements and validations significantly strengthened the ontology's capacity to model real-world hybrid threats described in the next section.

### Phase 4 

***Case study: hybrid threats to U.S. election infrastructure***

*Introduction*

In October 2024, an article in *WIRED* magazine ^x^ highlighted the evolving nature of cyber threats to U.S. election infrastructure. While state-sponsored actors such as Russia, China, and Iran have historically interfered in U.S. elections, the article identified a shift in the threat landscape. The Department of Homeland Security reported that financially motivated cybercriminals now pose a more significant threat of disruptive attacks on election systems.

This case study examines these emerging threats, particularly in the context of 5G-enabled infrastructure, to demonstrate how the ontological framework's emphasis on **resilience through risk reduction** effectively captures and analyses them. The study explores the motivations and tactics of various threat actors, the vulnerabilities of election infrastructure, and the potential impact of cyberattacks on democratic processes.

*5G-enabled disinformation propagation*

**Figure 5.**
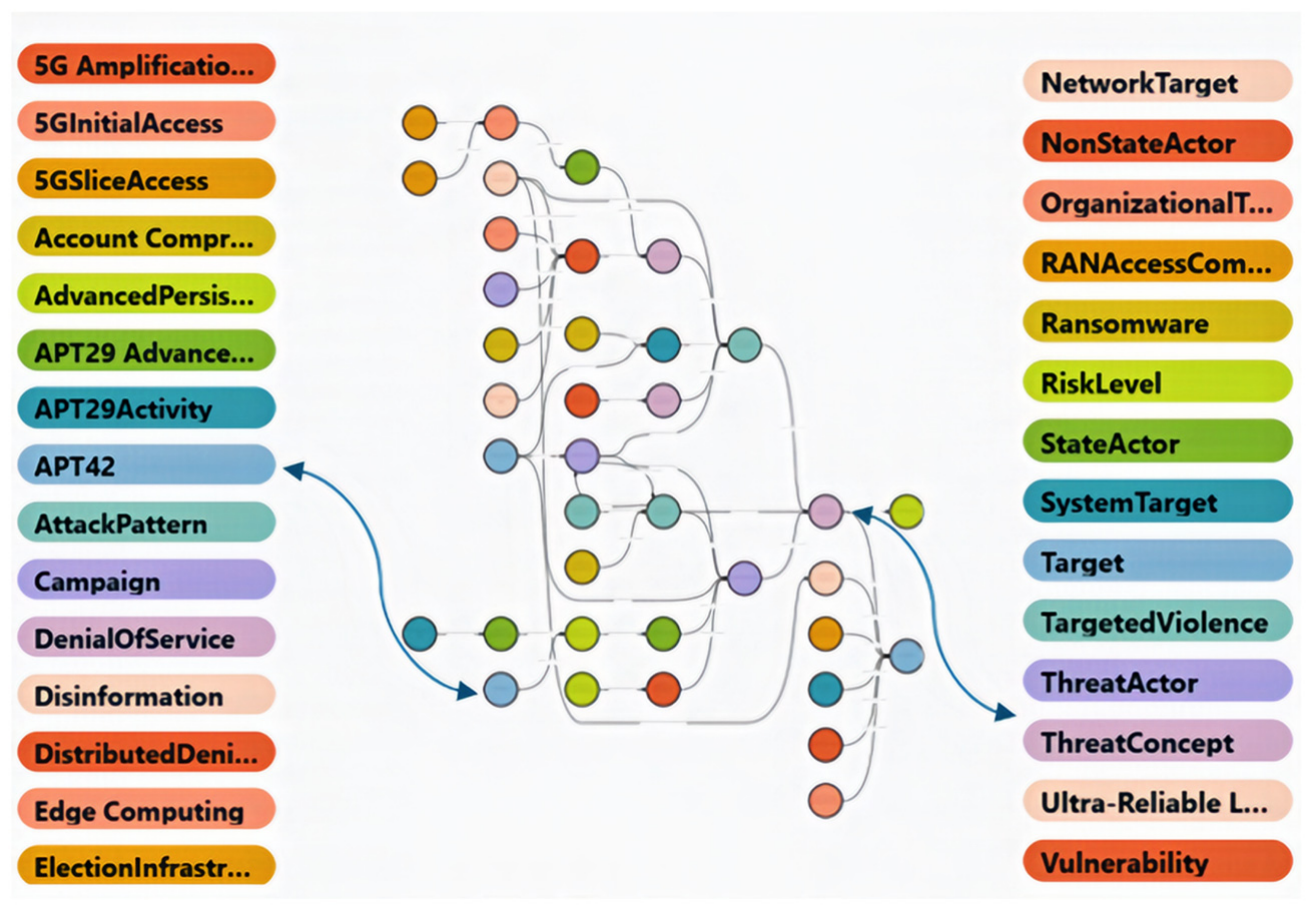
Modelling disinformation threats and incorporating APT42 into the ontology. Derived from the STARDOG Knowledge Graph, the figure depicts the structured modelling of disinformation threats within the ontology. The colour-coded classes and subclasses correspond to the central hierarchical structure, illustrating how disinformation campaigns, adversarial tactics, and infrastructure vulnerabilities interconnect. The STIX 2.1 category ThreatConcept, the root class, is positioned towards the bottom right. The relationships between threat actors, campaigns, vulnerabilities, and 5G-specific components highlight how the ontology enables risk-informed decision-making and structured intelligence analysis. By supporting dynamic threat integration and contextual risk assessment, this framework provides a scalable and extensible approach to mitigating hybrid threats in complex 5G environments. A key feature here is the seamless integration of APT42, which demonstrates the ontology’s adaptability in incorporating new threat actors without requiring major modifications. This flexibility reinforces the ontology’s resilience, ensuring that evolving hybrid threats — particularly those leveraging 5G infrastructure for disinformation operations — can be accurately mapped and assessed.

The *WIRED* article highlights how Russian, Chinese, and Iranian state actors actively spread disinformation throughout the 2024 campaign season. Our ontology captures this critical threat vector through its Disinformation class structure ( Figure 5., bottom left, in pink), specifically addressing how 5G infrastructure can be exploited for information operations.

The high-bandwidth, low-latency characteristics of 5G networks create new opportunities for disinformation campaigns. Our ontology models this within the Disinformation class through:

- propagationChannel property: maps how specific 5G network components, such as network slices or edge computing nodes, can be exploited for targeted disinformation delivery
- amplificationMethod property: captures techniques that leverage 5G capabilities for the enhanced spread of false information

*5G infrastructure: enhancing threat detection and response*

The ontology identifies key 5G-specific amplification mechanisms that (identified by different colours in Figure 5), while potentially exploitable, enable advanced protective measures:

- Network slicing capabilities allowing precise threat isolation and targeted defence deployment (5GSliceAccess, top left)
- Edge computing supporting rapid local threat detection and containment (bottom left)
- Enhanced mobile broadband, enabling sophisticated authenticity verification and deepfake detection
- Ultra-reliable low-latency communication facilitating real-time threat monitoring and response (bottom right)

*Resilience through structure*

Our framework ( Figure 5) supports risk reduction for disinformation threats by:

- Linking disinformation campaigns to specific threat actors (Campaign and ThreatActor classes, off-centre, towards the bottom, left and right, respectively)
- Mapping propagation methods to network components (5GInitialAccess, top left)
- Enabling analysis of amplification patterns (5GAmplificationMechanism, top left)
- Supporting early detection through pattern recognition (AttackPattern, middle left)

This structured approach to modelling disinformation threats, particularly in their relationship to 5G infrastructure, enhances our ability to identify and counter such campaigns, contributing to overall election infrastructure resilience.

*Dynamic response to emerging threats*

The analysis of the *WIRED* magazine article highlighted potential gaps in the ontology's structure, particularly in representing evolving threat actors and their activities. In response, three key additions were made: **APT42**, to capture newly identified adversarial behaviour; **Campaign**, to model coordinated disinformation and cyber operations; and **ElectionInfrastructure**, to account for critical election-related assets as explicit targets. Figure 5. illustrates the integration of APT42 within the ontology. The code snippet below presents the RDF/Turtle representation of the newly introduced Campaign class, followed by the snippet that defines the ElectionInfrastructure class, now a subclass of Target.


fght:Campaign a owl:Class ;
  rdfs:label "Campaign" ;
  rdfs:comment """A coordinated set of activities, undertaken by threat actors 
      employing a set of Tactics, Techniques, and Procedures (TTPs), to achieve 
      specific objectives, such as data exfiltration, espionage, or disruption of
      critical systems.""" ;
  fght:alignedWithStandard "STIX 2.1" ;
  fght:alignedWithMITRE "MITRE ATT&CK Matrix for Enterprise" ;
  fght:exampleUsage """The SolarWinds Campaign by APT29 employed multiple 
      TTPs to infiltrate supply chain systems and exfiltrate sensitive data.""" ;
  rdfs:subClassOf fght:NefariousActivity,
     [ a owl:Restriction ;
       owl:onProperty fght:targets ;
       owl:someValuesFrom fght:Target
     ] .



The **Campaign** class is a key addition to the 5G Hybrid Threat Ontology, introduced as part of the case study’s refinement process in response to election-related disinformation campaigns. It models coordinated adversarial activities, emphasising the use of Tactics, Techniques, and Procedures (TTPs) in achieving malicious objectives, such as data exfiltration, espionage, or critical system disruption.

A central aspect of this class is its explicit alignment with MITRE ATT&CK and STIX 2.1, ensuring interoperability with widely adopted standards for threat intelligence. These relationships are captured through:

- **fght: alignedWithStandard "STIX 2.1"** – ensuring compliance with structured threat intelligence-sharing frameworks.
- **fght: alignedWithMITRE "MITRE ATT&CK Matrix for Enterprise"** – linking the class to MITRE ATT&CK’s structured adversarial tactics.

The **exampleUsage** property contextualizes this class by referencing APT29’s SolarWinds campaign, illustrating how supply chain infiltration and TTP-driven attacks are formally modeled within the ontology.

Structurally, the Campaign class is defined as a subclass of NefariousActivity, ensuring a logical placement within the threat hierarchy. Additionally, the class incorporates a restriction-based property definition (owl:Restriction), which enforces structured relationships by requiring a Campaign to have specific targets, as defined under the fght:Target class. Within the case study, election infrastructure serves as a relevant example, highlighting how adversarial campaigns exploit critical infrastructure to spread disinformation and disrupt democratic processes.

The **ElectionInfrastructure** class was introduced to the 5G Hybrid Threat Ontology in response to the case study’s focus on hybrid threats targeting U.S. election systems. It models critical election-related systems and infrastructure components, ensuring that threats to election security are explicitly captured within the ontology.

This class is a subclass of Target (fght: Target, below), situating election infrastructure within the broader asset category that adversaries may have compromised or manipulated. The rdfs: comment property, provides a descriptive definition, specifying that it represents essential election-related infrastructure components, including voting systems, voter registration databases, and election management software.


# Election Infrastructure
fght:ElectionInfrastructure a owl:Class ;
  rdfs:subClassOf fght:Target ;
  rdfs:label "ElectionInfrastructure" ;
  rdfs:comment """Critical election-related systems and infrastructure components,
      specifically, the Cybersecurity Framework Election Infrastructure Profile""" ;
  fght:alignedWithNIST "NIST Critical Infrastructure Protection" ;
  fght:externalReference < https://www.nist.gov/publications/cybersecurity-framework-election-infrastructure-profile > .



This RDF/Turtle snippet acknowledges that The National Institute of Standards and Technology (NIST) categorises election infrastructure as ‘critical’ infrastructure, which requires protection and underscores its strategic importance and the necessity of resilience-driven security measures to safeguard democratic processes against emerging threats. Within this general framework, NIST has established a Cybersecurity Framework Election Infrastructure Profile to address the unique cybersecurity challenges faced by election systems. This profile provides election administrators and IT professionals with structured guidelines for managing and mitigating cyber risks associated with voting equipment and election-related information systems. The fght: externalReference displays the web link to the Profile. ^xi^

A critical feature of this class is its explicit alignment with NIST’s Critical Infrastructure Protection framework through the fght:alignedWithNIST "NIST Critical Infrastructure Protection" property. This alignment ensures the ontology remains consistent with widely recognised cybersecurity guidelines and risk management frameworks. NIST categorises election infrastructure as part of critical infrastructure, emphasising its strategic importance and the need for resilience-driven security measures.

By formally incorporating ElectionInfrastructure into the ontology, our model enhances structured threat intelligence and interoperability with national cybersecurity policies. The inclusion of this class provides a semantic foundation for analysing hybrid threats to democratic processes, particularly those leveraging 5G-enabled disinformation, cyber intrusions, and adversarial campaigns against election systems.

The discovery that **APT42** targeted presidential campaign accounts underscores the dynamic nature of hybrid threats and the necessity of a flexible ontological framework. According to Google's Threat Analysis Group, "APT42, which is believed to work for Iran's Revolutionary Guard Corps, targeted about a dozen people associated with the Trump and Biden campaigns this spring." ^xii^

APT42 is known for its cyber espionage and influence operations, often leveraging social engineering, credential harvesting, and targeted phishing campaigns against political entities, journalists, and dissidents. Unlike APT29, which primarily focuses on covert intelligence gathering and strategic cyber intrusion, APT42 exhibits a broader hybrid strategy, combining cyber, socio-political, and psychological operations to influence electoral processes.

The ontology’s structured approach enables the seamless integration of APT42 ( Figure 5) alongside threat actors like APT29, ensuring that adversarial tactics, targeting patterns, and attribution confidence are accurately mapped and semantically linked. The framework also supports rapid risk assessment and response initiation without requiring structural modifications, reinforcing its adaptability for tracking evolving hybrid threat campaigns.

*Historical foundation and evolution*

The ontology's foundation in historical data, particularly the documented activities of APT29 in the 2016 election interference ^xiii^ and the 2020 SolarWinds campaign, ^xiv^ provides crucial pattern recognition capabilities. By incorporating these past threat scenarios, the ontology enables comparison of current threats with established patterns, supports predictions of potential attack vectors, and informs risk reduction strategies based on past incidents. Furthermore, this historical perspective helps validate the effectiveness of the classification framework by ensuring its ability to capture evolving adversarial tactics.

*5G infrastructure vulnerabilities and access points*

The ontology explicitly models 5G-specific attack vectors through dedicated classes that capture entry points and vulnerabilities within the network ( Figure 5). These include 5GInitialAccess, which represents points of entry into 5G infrastructure; 5GSliceAccess, which models threats to network slice isolation; and RanAccessCompromise, which reflects vulnerabilities in radio access networks. These structural additions are particularly relevant as they highlight how threat actors could exploit 5G infrastructure to compromise election-related network slices, disrupt localised services through edge computing nodes, and target virtualised network components.

*Threat actor motivation*

The case study also highlights the need to refine the ontology’s representation of Non-State Actor motivations ( Figure 5, top right). While the ontology currently models ideologically driven actors under IdeologicallyMotivatedPerson, the prominence of financially motivated cybercriminals in election interference suggests a necessary enhancement to the framework. This expansion demonstrates the ontology’s flexibility in evolving alongside emerging threat landscapes while maintaining its core structural integrity.

*Attack vectors and 5G infrastructure*

The *WIRED* magazine report identifies two primary attack patterns — ransomware attacks ( Figure 5, off-centre, towards the top right) targeting election-related systems and Distributed Denial of Service (DDoS) attacks on critical infrastructure (bottom left) — that align directly with ENISA’s 5G threat categories. These threats pose unique risks in 5G environments due to increased network surface area from virtualisation and network slicing, enhanced connectivity that enables more sophisticated DDoS campaigns, and critical dependencies between election systems and 5G infrastructure.

*Risk reduction through ontological modelling*

Our framework actively supports risk reduction by offering adaptive capability, enabling the rapid integration of new threat actors without requiring major structural modifications. Additionally, the ontology enhances pattern recognition through the standardised mapping of attack vectors to affected infrastructure components. Establishing transparent relationships between threats, vulnerabilities, and targets facilitates impact analysis, while its cross-domain approach ensures the integration of cyber and physical security considerations.

*Resilience enhancement*

The ontology strengthens resilience in multiple ways. It enables rapid threat actor classification, supports standardised response procedures, and facilitates threat intelligence sharing by structuring adversarial behaviours and attack vectors within a semantically rigorous framework. Moreover, its structured approach to vulnerability assessment ensures a more comprehensive understanding of systemic risks.

### Concluding insights

The case study illustrates how the 5G Hybrid Threat Ontology’s historical foundation and dynamic threat modelling capabilities support enhanced resilience through risk reduction. By capturing both established threat patterns and emerging risks, particularly in the context of 5G-enabled disinformation campaigns and election-related cyber threats, the framework provides a robust foundation for protecting critical election infrastructure. Its ability to integrate evolving adversarial tactics, refine risk assessments, and structure intelligence-sharing mechanisms underscores its value as a scalable and adaptable cybersecurity resource in hybrid threat environments.

### Validation

***SPARQL query validation and practical utility***

To validate the ontology's effectiveness in supporting actionable threat intelligence, we developed multiple SPARQL queries addressing key analytical scenarios relevant to hybrid threat analysis. These queries demonstrate how security analysts can systematically extract structured intelligence from the ontology to support operational decision-making.

Here, we present two representative queries that directly address the paper's core objectives: demonstrating the ontology's capability to model hybrid threats while supporting resilience through systematic risk reduction.

*Query 1: Comprehensive risk profile analysis*

This query identifies high-threat campaigns, their targeted vulnerabilities, associated threat actors, and malware arsenals – providing the multi-dimensional intelligence required for prioritised defensive planning.

**SPARQL query No. 1**


PREFIX fght: <https://purl.org/5g-hybrid-threats#>
PREFIX rdfs: <http://www.w3.org/2000/01/rdf-schema#>

SELECT ?vulnerability ?campaign ?threatActor ?malware ?riskLevel
WHERE {
 ?campaign a fght:Campaign ;
      fght:targets ?vulnerability ;
      fght:attributedTo ?threatActor .
 ?threatActor fght:hasThreatLevel fght:High .
 OPTIONAL { 
   ?malware fght:usedBy ?threatActor ;
      fght:hasRiskLevel ?riskLevel ;
      fght:MalwareName ?malwareName
 }
}
ORDER BY ?vulnerability ?threatActor


The query results in Table 2. demonstrate the ontology's practical utility across diverse threat scenarios and temporal ranges. The analysis reveals APT42's 2024 targeting of U.S. presidential campaign infrastructure, utilising 5G amplification mechanisms (Edge Computing and Network Slicing), while simultaneously capturing similarities with the Lazarus Group's 2017 WannaCry campaign. This dual perspective illustrates the ontology's capability to correlate contemporary election-focused threats with historical attack patterns, supporting the framework's *historical foundation* approach described earlier in the case study. The results also expose cross-actor patterns in malware arsenals – specifically the shared use of tools like Mimikatz and Tor Browser – providing comparative intelligence valuable for defensive planning. All identified threats carry High Risk assessments, enabling prioritised resource allocation. The breadth of coverage, spanning both election-specific vulnerabilities and general infrastructure targets, demonstrates that the ontology provides actionable intelligence across the full spectrum of hybrid threat scenarios rather than serving only narrow use cases.

**Table 2.** Risk Profile Analysis: High-Threat Campaigns and Associated Malware.

Vulnerability Target	Campaign	Threat Actor	Malware/Tool	Risk Level
US Presidential Campaign Vulnerability	APT42 Campaign 2024	APT42	Edge Computing	High Risk
US Presidential Campaign Vulnerability	APT42 Campaign 2024	APT42	Network Slicing	High Risk
Vulnerability	WannaCry	Lazarus Group	WannaCry	High Risk
Vulnerability	WannaCry	Lazarus Group	Mimikatz	High Risk
Vulnerability	WannaCry	Lazarus Group	wAgent	High Risk
Vulnerability	WannaCry	Lazarus Group	Bookcode	High Risk
Vulnerability	WannaCry	Lazarus Group	Tor Browser	High Risk

*Query 2: 5G-Enabled disinformation operations*

This query specifically addresses the case study's focus on election-related disinformation by linking adversarial operations to the 5G capabilities they exploit, their propagation mechanisms, and associated risk levels.

**SPARQL query No. 2**


PREFIX fght: <https://purl.org/5g-hybrid-threats#>
PREFIX rdfs: <http://www.w3.org/2000/01/rdf-schema#>

SELECT ?disinfoOp ?mechanism ?method ?channel ?risk
WHERE {
 ?disinfoOp a fght:Disinformation ;
        fght:usesAmplificationMechanism ?mechanism ;
        fght:amplificationMethod ?method ;
        fght:propagationChannel ?channel ;
        fght:hasRiskLevel ?risk .
 ?mechanism rdfs:label ?mechLabel .
}


The query results in Table 3. demonstrate the ontology's integration of threat mechanics with risk assessment for 5G-enabled disinformation operations. The analysis reveals how adversaries exploit specific 5G capabilities – Network Slicing for demographic targeting, Edge Computing for localised campaigns, and Ultra-Reliable Low-Latency Communication (URLLC) for real-time content adjustment. Each operation propagates through 5G Core infrastructure vulnerabilities and carries a High Risk assessment. This structured intelligence directly supports the case study's focus on election-related disinformation campaigns described in the WIRED article, enabling security teams to prioritise defensive measures based on both exploitation mechanisms and assessed severity. The query exemplifies the ontology's practical utility in linking adversarial tactics to infrastructure vulnerabilities, supporting resilience through systematic risk reduction rather than reactive threat response.

**Table 3.** 5G-Enabled Disinformation Operations and Amplification Mechanisms.

Disinformation Operation	5G Amplification Mechanism	Exploitation Method	Propagation Channel	Risk Level
5G Disinfo Operation	Network Slicing	Combined slice and edge exploitation	5G Core Vulnerability	High Risk
Network Slicing Attack	Network Slicing	Network slice manipulation	5G Core Vulnerability	High Risk
5G Disinfo Operation	Edge Computing	Combined slice and edge exploitation	5G Core Vulnerability	High Risk
5G DDoS Operation	Ultra-Reliable Low-Latency	URLLC exploitation	5G Core Vulnerability	High Risk

***Validation through Integration of Intelligence***

To further validate the effectiveness of the 5G Hybrid Threat Ontology in supporting resilience through risk reduction, we implemented a Python-based intelligence integrator that analyses threat data from multiple authoritative sources, including MITRE ATT&CK, CISA, and US-CERT. The code focuses explicitly on APT42 and related Iranian threat actors while monitoring for 5G infrastructure vulnerabilities and exploitation patterns. ^xv^

This Python code defines a class called *APT42IntelligenceIntegrator*, designed to gather and analyse threat intelligence data related to the activities of the Iranian state-sponsored APT42 threat actor group, particularly those targeting the 2024 U.S. presidential election and 5G infrastructure.

The class fetches data from various sources, including MITRE ATT&CK, CISA Known Exploited Vulnerabilities, CISA Alerts, US-CERT Current Activity, and Microsoft Security Guidance. It then analyses this data for keywords related to APT42, disinformation campaigns, and 5G technologies. The code assesses the risk level of identified threats based on predefined criteria and correlates patterns in the data to identify potential temporal trends and attack vectors. The results, including categorised findings and correlated patterns, are then printed to the console.

***Results***

The analysis of the Python code results demonstrated the ontology's practical value in supporting systematic threat classification, enabling risk-based decision-making, and facilitating a comprehensive understanding of attack patterns. The findings were successfully categorised into 82 distinct entries across the following categories: campaign targeting (18), 5G exploitation (51), and general activities (13), demonstrating precise alignment with the classification system.

The validation results from the intelligence integrator confirmed the relevance of 5G network slicing and edge computing vulnerabilities, demonstrating that the 5G Hybrid Threat Ontology effectively structures and analyses hybrid threats in alignment with real-world intelligence data.

The combined validation through SPARQL querying and real-world intelligence integration demonstrates the ontology's effectiveness in capturing hybrid threats across multiple dimensions. The SPARQL queries successfully extracted structured intelligence spanning contemporary election threats (APT42 2024) and historical campaigns (WannaCry 2017), while the Python-based intelligence integrator categorised 82 distinct findings with 64.6% assessed as high-risk, enabling prioritised defensive strategies. This validation confirmed the ontology's capacity to support systematic threat classification and risk-based decision-making as designed.

However, the validation process – particularly the analysis of the WIRED case study – also revealed limitations in the ontology's ability to capture increasingly complex relationships between state actors, disinformation campaigns, and the exploitation of 5G infrastructure. Modelling how different APT groups coordinate using 5G amplification mechanisms for information operations proved challenging with the preliminary ontology's Description Logic (DL) expressivity. These insights from real-world threat scenarios drove systematic enhancements to the ontology's formal capabilities, as described in the following section.

## Enhancing the preliminary ontology's expressivity for advanced hybrid threat modelling

The *WIRED* case study revealed limitations in our ontology's ability to capture complex relationships between state actors, disinformation campaigns, and exploitation of 5G infrastructure. Modelling how different APT groups coordinate using 5G amplification mechanisms for disinformation operations was particularly challenging. This highlighted the need for enhanced Description Logic (DL) expressivity to represent these sophisticated hybrid threats better.

### DL expressivity enhancements in the aftermath of the WIRED case study

Starting with a baseline DL expressivity of ALHI(O), we systematically enhanced the ontology's expressivity through several key improvements. These enhancements, documented in the ‘docs/Expressivity’ folder of our GitHub repository, significantly expand the ontology's ability to represent and reason about complex relationships between threat actors and hierarchical structures.

Based on the enhancements we implemented, the current DL expressivity is ALCHFRSIOQ(D), representing a significant expansion from the initial ALHI(O) baseline. The enhanced DL expressivity achieved through these systematic improvements directly strengthens the ontology's capability to model and reason about hybrid threats in 5G environments. The enhanced expressivity enables the 5G Hybrid Threat Ontology to capture sophisticated attack patterns where state and non-state actors collaborate across different threat vectors by implementing complex role chains, transitivity, and qualified cardinality restrictions.

The ontology can now model how APT groups share infrastructure and tools while maintaining distinct operational characteristics, as well as how different sections of vulnerability analyses interrelate to form a comprehensive threat landscape. This enhanced modelling capability directly supports risk reduction by enabling more nuanced threat detection and improving anticipation of complex, multi-vector attacks that characterise the hybrid threat landscape in 5G environments.

### Ontology metrics and complexity analysis

Following the DL expressivity enhancements, our ontology's formal structure expanded significantly. Comparing metrics between Phase 2 (January 2, 2025) and the final version (January 24, 2025) reveals substantial growth in complexity and coverage. The details are presented in the docs/Metrics folder of our GitHub repository, with key findings summarised below.

Overall, the metrics indicate a clear trend of increasing complexity, expressiveness, and interconnectedness across the three phases of ontology development. The growth in logical axioms, classes, object properties, and assertions demonstrates significant progress in enhancing ontology's capabilities for hybrid threat modelling and risk reduction in 5G environments.

These metrics illustrate the evolution of the 5G Hybrid Threat Ontology from a basic taxonomic structure to a sophisticated knowledge representation system capable of modelling complex hybrid threats in 5G environments. The increased axiom count and relationship complexity directly support enhanced reasoning capabilities, particularly in tracking the propagation of attacks and group-level threat behaviours.

The improved expressivity has proven particularly valuable for modelling disinformation campaigns. It enables the representation of complex relationships between state actors and their coordinated exploitation of 5G infrastructure for information operations.

## Reproducibility, Community Engagement, and Ontology Extension

To support reproducibility and encourage community contributions, all ontology development materials, validation code, and case study data are publicly available in the SecOntologyLab GitHub repository ( https://github.com/SecOntologyLab/5G-hybrid-threats). The repository provides structured resources organised for different research needs.

*Core ontology:* These files include the complete 5G Hybrid Threat Ontology in RDF/Turtle format (ontology_fght.ttl), SHACL validation shapes (shapes_fght.ttl), and a Python-based validation script (validation_fght.py) enabling researchers to verify ontological consistency. These files represent the validated framework described in Phases 1-3 and incorporate enhancements from the election infrastructure case study.

*Validation and expressivity materials:* These describe the ontology's DL expressivity evolution through seven comprehensive test cases demonstrating specific features: disjoint classes (C), property chains (R), transitive properties with inverse roles (SIR), qualified cardinality restrictions (Q), and functional properties (F). Each test includes executable SPARQL queries, Python implementation code, query results, and corresponding Turtle representations from the ontology. These materials enable researchers to reproduce our reasoning validation and understand how specific DL constructs support hybrid threat modelling.

*Case study materials:* These provide the complete text of the WIRED magazine article, the Python-based APT42 intelligence integrator code, execution results across 82 categorised findings, and detailed analysis documentation. This enables replication of our validation methodology, integrating real-world threat intelligence from MITRE ATT&CK, CISA, and US-CERT sources.

*Metrics and evolution analysis:* This documents the ontology's growth across development phases, with quantitative comparisons ( Table 1– Table 3) showing class expansion, property additions, and increases in axiom count. These supplementary materials support understanding the iterative development process without adding excessive technical detail to the main paper.

*Extending the ontology:* Researchers can contribute by (1) adding new threat actor classes following the StateActor/NonStateActor taxonomy structure, (2) incorporating additional 5G amplification mechanisms as subclasses of 5GAmplificationMechanism, (3) defining new campaigns with required properties validated through SHACL constraints, or (4) integrating threat intelligence from additional sources following the Python intelligence integrator pattern. All contributions should maintain alignment with STIX 2.1, ENISA taxonomies, and MITRE ATT&CK frameworks through the established annotation property system (alignedWithStandard, alignedWithENISA, alignedWithMITRE).

*Replicating validation:* To reproduce our validation process: (1) clone the repository, (2) execute validation_fght.py to verify SHACL constraints against the ontology, (3) load the ontology into Protégé and run HermiT, Pellet, or ELK reasoners to validate logical consistency, (4) execute the DL expressivity test queries documented in the docs/Expressivity directory, and (5) run the MITRE ATT&CK intelligence integrator to analyze current threat data. Complete execution instructions and dependencies are provided in the repository README.

The dataset is licensed under the Creative Commons Attribution 4.0 International (CC-BY 4.0) license, allowing for reuse and adaptation with proper attribution. The persistent identifier (PURL) https://purl.org/5g-hybrid-threats provides stable access to the ontology namespace. Community engagement through GitHub issues and pull requests supports collaborative refinement of the framework as 5G threat landscapes evolve.

## Conclusion

The 5G Hybrid Threat Ontology, introduced in this Open Letter, has been developed from the ground up. It integrates established cybersecurity frameworks while providing a novel, structured approach to modelling hybrid threats in 5G infrastructure. This initiative aims to achieve resilience through effective risk reduction rather than focusing solely on threat elimination or prediction.

By presenting an iterative approach to building a structured ontological framework and integrating insights from established frameworks such as ENISA’s 5G Threat Landscape, STIX 2.1, and MITRE ATT&CK, the ontology captures the complex interplay of adversarial tactics, 5G-specific vulnerabilities, and cascading risks across interconnected domains. Furthermore, by aligning with the ‘JRC/CoE hybrid threats ecosystem’, the framework reflects a broader European security strategy for countering hybrid threats through structured intelligence modelling and resilience-building. This connection strengthens the ontology’s ability to cohesively incorporate socio-political, cyber, and physical security dimensions, ensuring its relevance for policy and operational cybersecurity efforts.

The ongoing evolution and proliferation of hybrid threats and rapid advancements in 5G infrastructure necessitate continual refinement of the proposed framework. Future research will build on the phased development of the 5G Hybrid Threat Ontology, further refining its expressivity and scalability. Comparing ontology metrics across development phases demonstrates its progression from an initial, compact structure to an increasingly comprehensive framework.

The subsequent growth phase will focus on scaling the 5G Hybrid Threat Ontology by leveraging graph-based reasoning and expanded cybersecurity datasets. A key priority will be integrating structured threat intelligence sources, such as ENISA’s 5G Threat Landscape Report, into a linked data environment that enhances risk assessment and resilience modelling. Given the ontology’s current use within STARDOG’s Knowledge Graph framework, future work will explore advanced reasoning capabilities, including the potential for STARDOG’s Virtual Graphs to dynamically integrate external cybersecurity datasets without moving or copying the data or requiring complete RDF conversion. Additionally, efforts will be made to extend semantic relationships between hybrid threat actors, infrastructure vulnerabilities, and attack campaigns, ensuring the ontology remains adaptable to evolving security challenges. While machine learning (ML) may play a future role in automating data extraction and classification, the immediate next steps will prioritise enhancing the ontology’s expressivity, expanding its integration with structured threat intelligence frameworks, and refining validation mechanisms to support practical cybersecurity applications. The 5G Hybrid Threat Ontology will evolve as a valuable tool for resilience-focused cybersecurity intelligence by progressively building on the structured knowledge representation established in this work.

## Data Availability

The 5G Hybrid Threat Ontology dataset supports this study and is publicly available in the SecOntologyLab GitHub repository: https://github.com/SecOntologyLab/5G-hybrid-threats. This dataset includes: The 5G Hybrid Threat Ontology in RDF/Turtle format (.ttl), modelling hybrid threats targeting 5G infrastructure. (…/Decoding5G/ontology/ontology_fght.ttl) SHACL validation rules for ensuring ontology consistency. (…/Decoding5G/ontology/shapes_fght.ttl) Python scripts used for ontology validation and integration with STIX and ENISA frameworks. (…/Decoding5G/scripts/validation_fght.py) Supplementary documentation: DL expressivity evolution analysis demonstrating ALCHFRSIOQ(D) enhancements. (…/Decoding5G/docs/Expressivity/) Ontology metrics documenting growth across development phases. (…/Decoding5G/docs/Metrics/) MITRE ATT&CK intelligence integrator with APT42 validation results. (…/Decoding5G/docs/MITRE_ATTACK/) Election infrastructure case study materials including WIRED article analysis. (…/Decoding5G/docs/Case_Study/) The dataset is licensed under a Creative Commons Attribution 4.0 International License (CC-BY 4.0), allowing for reuse and adaptation with proper attribution. 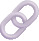
 Persistent Identifier (PURL): https://purl.org/5g-hybrid-threats

## Footnotes

^i^ The EU's CER (Critical Entities Resilience) Directive can be found here: https://eur-lex.europa.eu/eli/dir/2022/2557/oj/eng

^ii^ The full text of the NIS2 (Network and Information Security 2) is available here: https://eur-lex.europa.eu/eli/dir/2022/2555/2022-12-27/eng

^iii^ Users can pull our ontology from the GitHub repository and incorporate it into a STARDOG Knowledge Graph by opening up a free account at: https://cloud.stardog.com/get-started

^iv^ https://www.hybridcoe.fi/

^v^ European Commission. (2016). Joint Communication to the European Parliament and the Council, Joint Framework on Countering Hybrid Threats: A European Union response. *JOIN/2016/018*. eur-lex.europa.eu/legal-content/EN/TXT/PDF/?uri=CELEX:52016JC0018

^vi^ NATO's Strategic Concept documents have been developed and updated periodically since the end of the Cold War. These documents outline NATO's enduring purpose, core tasks, and strategic objectives, adapting to the evolving global security environment. The series includes key documents from the following years: 1991, 1999, 2010, and 2022, the most recent. NATO. (2022). *Strategic Concept*. https://www.nato.int/strategic-concept/

^vii^ See, for example, its contributions to Research and Analysis - Hybrid CoE - The European Centre of Excellence for Countering Hybrid Threats and real-world examples and case studies at Publications and readings - Hybrid CoE - The European Centre of Excellence for Countering Hybrid Threats

^viii^ For an overview of the taxonomy and its visualisation, go to: https://cybersecurity-atlas.ec.europa.eu/cybersecurity-taxonomy

^ix^ The complete SHACL Shapes file (latest version), Python script, and Turtle ontology file are available in our GitHub repository: https://github.com/SecOntologyLab/5G-hybrid-threats.

^x^ Cybercriminals Pose a Greater Threat of Disruptive US Election Hacks Than Russia or China | WIRED. The article is also available on our GitHub repository in the docs directory.

^xi^ https://www.nist.gov/publications/cybersecurity-framework-election-infrastructure-profile

^xii^ https://www.wired.com/story/iran-apt42-trump-biden-harris-phishing-targeting/

^xiii^ As reported" in DNC Lawsuit Against Russia Reveals New Details About 2016 Hack | WIRED “...Russian intelligence group Cozy Bear—the GRU-affiliated hacker group, also known as APT29—infiltrated the DNC network as far back as July 27, 2015, nearly a year before the leaks of the pilfered material began.”

^xiv^ As reported by MITRE ATT&CK at SolarWinds Compromise, Campaign C0024 | MITRE ATT&CK® “The SolarWinds Compromise was a sophisticated supply chain cyber operation conducted by APT29 that was discovered in mid-December 2020.”

^xv^ The Python code and the run results are available in our GitHub repository, in the directory ‘…/docs/MITRE ATT&CK’.
