# Supplementary material for: Decoding 5G security: toward a hybrid threat ontology
Source: Open Res Eur. 2025 Oct 22;4:34. Originally published 2024 Feb 19. [Version 3] doi: 10.12688/openreseurope.16916.3 (PMC12489459; doi:10.12688/openreseurope.16916.3)
Supplement: Peer review history

# Reviewer report: Reviewer response for version 3

**DOI:** 10.21956/openreseurope.23335.r62636

**Published:** 2025-11-05

**Authors:** Geeta Sandeep Nadella

**Affiliations:** 1 University of the Cumberlands, Williamsburg, Kentucky, USA

Thank you for your diligent work in revising the manuscript. The latest version demonstrates a marked improvement in clarity, structure, and scholarly rigor, effectively addressing the core concerns raised during the initial review. The addition of the "Design Rationale and Competing Approaches" section is particularly valuable, as it situates your work within the broader research landscape and justifies key technical choices with clear reasoning.

 The enhanced case study, now featuring detailed SPARQL queries with full code and analytical captions, strengthens the paper's practical relevance. It convincingly showcases the ontology's utility for real-world threat intelligence and risk assessment, moving beyond theoretical discussion to demonstrate operational value.

 Your decision to relocate highly technical content—such as ontology metrics and detailed DL expressivity analysis—to supplementary GitHub materials strikes an excellent balance. It maintains the Open Letter's accessibility while ensuring that interested researchers can delve into the technical foundations. This approach respects the format's purpose without sacrificing depth.

 The new "Reproducibility and Community Engagement" section is a significant strength. By providing clear pathways for validation, extension, and collaboration, you have transformed the work from a standalone contribution into a living, community-oriented resource. This aligns well with open science principles and encourages broader adoption and refinement.

 The refined integration of established frameworks—STIX, MITRE ATT&CK, and ENISA—alongside the "JRC/CoE hybrid threats ecosystem" now presents a more cohesive and interoperable model. The structured approach to aligning with these standards enhances the ontology's credibility and utility for cybersecurity practitioners.

 Overall, the manuscript now presents a compelling, well-justified initiative that bridges policy, technical implementation, and collaborative research. It successfully communicates both the conceptual framework and its practical application, making a valuable contribution to the field of 5G security and hybrid threat modelling. The revisions reflect a thoughtful and thorough engagement with reviewer feedback, resulting in a more polished, accessible, and impactful Open Letter.

 The revised manuscript successfully addresses the majority of reviewer concerns and presents a well-structured, technically sound Open Letter. It provides a clear rationale, supports claims with citations, and is written in accessible language. Minor improvements in critical engagement and community guidance would further strengthen the work. The article is scientifically sound and makes a valuable contribution to the field of 5G security and hybrid threat modelling.

Where applicable, are recommendations and next steps explained clearly for others to follow? (Please consider whether others in the research community would be able to implement guidelines or recommendations and/or constructively engage in the debate)

Partly

Does the article adequately reference differing views and opinions?

Partly

Are all factual statements correct, and are statements and arguments made adequately supported by citations?

Yes

Is the rationale for the Open Letter provided in sufficient detail? (Please consider whether existing challenges in the field are outlined clearly and whether the purpose of the letter is explained)

Yes

Is the Open Letter written in accessible language? (Please consider whether all subject-specific terms, concepts and abbreviations are explained)

Yes

Reviewer Expertise:

5G Security Telecommunications Security Hybrid Threats Critical National Infrastructure (CNI) Protection Network Slicing Security Supply Chain Risk Management (SCRM) Geopolitics of Technology Standardization and Governance

I confirm that I have read this submission and believe that I have an appropriate level of expertise to confirm that it is of an acceptable scientific standard.

# Reviewer report: Reviewer response for version 2

**DOI:** 10.21956/openreseurope.21432.r61148

**Published:** 2025-10-01

**Authors:** Geeta Sandeep Nadella

**Affiliations:** 1 University of the Cumberlands, Williamsburg, Kentucky, USA

- **Critical Engagement with Related Work:** While the “Related work” section is comprehensive, it could be strengthened by explicitly contrasting the proposed ontology with existing models and justifying design choices in light of competing approaches.
- **Community Engagement and Reproducibility:** Although the ontology is publicly available, the manuscript could include clearer instructions for researchers to replicate the validation process or contribute to the ontology’s development.
- **Case Study Depth:** The election infrastructure case study effectively demonstrates the ontology’s applicability but could be enhanced with more detailed SPARQL query examples or reasoning outputs to illustrate practical utility.
- **Balance of Technical Detail:** As noted by Reviewer 1, some technical sections (e.g., reasoner performance metrics) may be overly detailed for an Open Letter. Consider moving highly technical content to supplementary materials or a dedicated technical report.

Where applicable, are recommendations and next steps explained clearly for others to follow? (Please consider whether others in the research community would be able to implement guidelines or recommendations and/or constructively engage in the debate)

Partly

Does the article adequately reference differing views and opinions?

Partly

Are all factual statements correct, and are statements and arguments made adequately supported by citations?

Yes

Is the rationale for the Open Letter provided in sufficient detail? (Please consider whether existing challenges in the field are outlined clearly and whether the purpose of the letter is explained)

Yes

Is the Open Letter written in accessible language? (Please consider whether all subject-specific terms, concepts and abbreviations are explained)

Yes

Reviewer Expertise:

The revised manuscript successfully addresses the majority of reviewer concerns and presents a well-structured, technically sound Open Letter. It provides a clear rationale, supports claims with citations, and is written in accessible language. Minor improvements in critical engagement and community guidance would further strengthen the work. The article is scientifically sound and makes a valuable contribution to the field of 5G security and hybrid threat modelling.

I confirm that I have read this submission and believe that I have an appropriate level of expertise to confirm that it is of an acceptable scientific standard, however I have significant reservations, as outlined above.

# Response

**Published:** 2025-10-07

**Authors:** R. Andrew Paskauskas

**Affiliations:** Lithuanian Cybercrime Center of Excellence for Research,Training and Education, Lithuania

We sincerely thank the reviewer for their thorough evaluation and constructive recommendations. The feedback has substantially strengthened the manuscript, and we appreciate the recognition that our work "makes a valuable contribution to the field of 5G security and hybrid threat modelling."

**Addressing Primary Recommendations** **Critical Engagement with Related Work**:

 We have added a new section titled "Design Rationale and Competing Approaches" (~440 words) that explicitly contrasts our ontology with existing frameworks. This section justifies key technical decisions—including knowledge representation language selection, validation architecture, ontological structure, and framework integration strategy—by comparing our approach with Bernardini et al.'s 5G security ontology, Salazar et al.'s mutation-based approach, Randles et al.'s IBCLO framework, Mahmud et al.'s SDN security ontology, and Globa et al.'s telecommunications model. Each design choice is contextualized against specific alternatives, demonstrating systematic evaluation rather than arbitrary architectural decisions.

**Community Engagement and Reproducibility**:

 We have incorporated a comprehensive "Reproducibility, Community Engagement, and Ontology Extension" section (~500 words) providing: (1) structured organization of GitHub repository materials by purpose (core files, validation code, case study data, metrics analysis), (2) step-by-step instructions for replicating our validation process across SHACL constraints, reasoner testing, DL expressivity verification, and MITRE ATT&CK intelligence integration, (3) concrete guidance on extending the ontology with new threat actors, 5G mechanisms, campaigns, and threat intelligence sources, and (4) contribution workflows through GitHub. This directly addresses the reviewer's concern about enabling research community implementation and constructive engagement.

**Case Study Depth**:

 We have enhanced the case study with two detailed SPARQL query examples presented in publication-ready tables with comprehensive captions. Query 6 (Risk Profile Analysis) demonstrates multi-dimensional intelligence aggregation across vulnerabilities, campaigns, threat actors, and malware with 7 result rows spanning both contemporary (APT42 2024) and historical (WannaCry 2017) threats. Query A (5G-Enabled Disinformation Operations) illustrates the integration of threat mechanics with risk assessment, showing how adversaries exploit specific 5G capabilities (Network Slicing, Edge Computing, URLLC) with 4 categorized attack instances. Both queries include complete SPARQL code, results tables, and analytical captions explaining operational significance for security practitioners.

**Balance of Technical Detail**:

 Following Reviewer 1's concerns and this recommendation, we have relocated Tables 1, 3, and 4 (ontology metrics), detailed DL expressivity analysis with all seven test cases, and the extended design rationale to GitHub supplementary materials in the docs/Metrics and docs/Expressivity directories. The main paper now references these materials for interested researchers while maintaining focus on core contributions appropriate for an Open Letter format.

**Addressing Supplementary Assessment Questions**

 We believe the revisions have strengthened the manuscript's performance on the two areas initially assessed as "Partly":

**"Does the article adequately reference differing views and opinions?"**

 The new Design Rationale section systematically contrasts our approach with six competing ontological frameworks (Bernardini et al., Rivadeneira & Gómez, Salazar et al., Randles et al., Mahmud et al., Globa et al.), explicitly acknowledging their strengths while explaining why alternative approaches were insufficient for hybrid threat modelling in 5G contexts. This demonstrates engagement with diverse perspectives across 5G security ontologies, general cybersecurity ontologies, network-focused approaches, and IoT security frameworks.

**"Where applicable, are recommendations and next steps explained clearly for others to follow?"**

 The Reproducibility section provides actionable guidance through:

 (1) five concrete steps for validation replication with specific tool names and file paths,

 (2) four specific extension pathways (new threat actors, 5G mechanisms, campaigns, intelligence sources) with structural requirements,

 (3) explicit alignment requirements maintaining STIX 2.1, ENISA, and MITRE ATT&CK compatibility, and

 (4) GitHub-based contribution workflows with issue tracking and pull request mechanisms. These instructions enable the research community to both implement our framework and contribute to its evolution.

 We are grateful for the reviewer's recognition that the work is "technically sound" and "well-structured," and we believe these revisions fully address the identified areas for improvement while maintaining the accessible, high-level perspective appropriate for an Open Letter.

# Reviewer report: Reviewer response for version 2

**DOI:** 10.21956/openreseurope.21432.r51855

**Published:** 2025-05-30

**Authors:** I.J. Da Silva Oliveira

**Affiliations:** 1 University of Twente, Enschede, The Netherlands

I consider that the author has now improved the paper enough for indexing, with new interesting additions. However, some parts seem unnecessary: (a) Ontology metrics (Tables 1, 3, and 4), (b) description logic expressivity considerations, (c) reasoner performance comparison. These things can be easily checked on proper software by the reader. I appreciate the initiative to better document the ontologies.

Where applicable, are recommendations and next steps explained clearly for others to follow? (Please consider whether others in the research community would be able to implement guidelines or recommendations and/or constructively engage in the debate)

No

Does the article adequately reference differing views and opinions?

No

Are all factual statements correct, and are statements and arguments made adequately supported by citations?

Partly

Is the rationale for the Open Letter provided in sufficient detail? (Please consider whether existing challenges in the field are outlined clearly and whether the purpose of the letter is explained)

Partly

Is the Open Letter written in accessible language? (Please consider whether all subject-specific terms, concepts and abbreviations are explained)

Yes

Reviewer Expertise:

Conceptual modeling, ontology engineering, Applied Ontology, Enterprise Modeling, Risk Management and Cybersecurity

I confirm that I have read this submission and believe that I have an appropriate level of expertise to confirm that it is of an acceptable scientific standard.

# Response

**Published:** 2025-10-07

**Authors:** R. Andrew Paskauskas

**Affiliations:** Lithuanian Cybercrime Center of Excellence for Research,Training and Education, Lithuania

We are deeply grateful to the reviewer for the thorough reconsideration and approval of our manuscript. The evolution from the initial assessment to the current endorsement reflects our commitment to addressing substantive concerns, and we sincerely appreciate the recognition that the paper now contains "new interesting additions" sufficient for indexing.

**Addressing Technical Detail Concerns**

 We have directly implemented the reviewer's recommendations regarding technical content that "can be easily checked on proper software by the reader":

**(a) Ontology metrics (Tables 1, 3, and 4)**: All three metric tables have been relocated to the GitHub repository's docs/Metrics directory, along with a comprehensive analysis document titled "Core Metrics Evolution" that provides detailed quantitative comparisons and growth percentages across development phases. The main paper now contains only Table 2 (Reasoner Performance Comparison), which directly validates Phase 3 development and cannot be easily reproduced without our specific ontology file.

**(b) Description Logic expressivity considerations**: The detailed DL expressivity analysis, including all seven test cases with SPARQL queries, Python implementation code, query results, and Turtle representations, has been moved to docs/Expressivity/DL_Expressivity_Results.docx in the GitHub repository. The main paper retains only a condensed explanation (~150 words) within the "Design Rationale and Competing Approaches" section, sufficient to justify our architectural choices without overwhelming technical detail.

**(c) Reasoner performance comparison**: While Table 2 remains in the main text as direct evidence of Phase 3 validation, we have streamlined the surrounding discussion to focus on validation outcomes rather than technical performance minutiae. Extended analysis of reasoner selection trade-offs has been incorporated into the Design Rationale section's explanation of validation architecture choices.

**Documentation Enhancement**

 We appreciate the reviewer's acknowledgment of our ontology documentation efforts. The GitHub repository now provides comprehensive materials organized for different research needs: core ontology files with validation scripts, complete case study materials including the WIRED article and Python intelligence integrator, all technical appendices (metrics, expressivity tests, extended design rationale), and step-by-step instructions for validation replication and ontology extension. The manuscript now balances the Open Letter format's accessibility requirements with the technical rigor necessary for reproducible research. Technical readers can access complete validation materials through GitHub, while the main paper maintains focus on conceptual contributions, practical applications, and strategic implications for 5G security.

 We thank the reviewer again for the constructive engagement that has significantly improved the manuscript's clarity and accessibility while preserving its technical foundation.

# Reviewer report: Reviewer response for version 1

**DOI:** 10.21956/openreseurope.18282.r42665

**Published:** 2024-09-14

**Authors:** I.J. Da Silva Oliveira

**Affiliations:** 1 University of Twente, Enschede, The Netherlands

The paper concerns understanding and modelling hybrid threats in the context of 5G technology, which is an important problem. However, the presentation is poorly made so the contributions are unclear.

 The paper does not give a general overview of its sections, as is usual in scientific publications. This makes it harder to follow the content as it is unclear what is intended to be a contribution and what is just the presentation of other materials.

 On page 4, the taxonomy of threats comes out of nowhere after mentioning a Google Scholar search.

 No related work section is given.

 The concept of hybrid threat is crucial in the paper but is stated elsewhere ( https://www.hybridcoe.fi/hybrid-threats-as-a-phenomenon/), not discussed in the paper.

 The ontology development is confusing. What were the tools employed to make the ontology? What was the methodology? Why not employ a foundational ontology? Why not extend an existing reference domain ontology?

 The proposed ontology is not available. Assessing its merits is, therefore, harder.

 Figures 1, 2, and 5 are too small. 

 RDF/OWL snippets are not relevant.  They should be removed. So, Tables 2, 4, 5, 7, and 9 should be removed.

 Is the "conceptual model" of Figure 2 a contribution?

 The paper has too many definitions and descriptions, resembling a technical report. Instead, it should focus on key aspects of the proposed ontology, and leave definitions in the ontological artefact and/or a documentation website.

 Figure 3 has a weird notation. Recommendation: use UML class diagram.

 Table 3. ENISA Threat Landscape 2022 report. Is it just repeating the report content?

 There is an unnamed table on page 22.

 The paper is unreasonably long. Its contribution is hard to identify and assess.

Where applicable, are recommendations and next steps explained clearly for others to follow? (Please consider whether others in the research community would be able to implement guidelines or recommendations and/or constructively engage in the debate)

No

Does the article adequately reference differing views and opinions?

No

Are all factual statements correct, and are statements and arguments made adequately supported by citations?

Partly

Is the rationale for the Open Letter provided in sufficient detail? (Please consider whether existing challenges in the field are outlined clearly and whether the purpose of the letter is explained)

Partly

Is the Open Letter written in accessible language? (Please consider whether all subject-specific terms, concepts and abbreviations are explained)

Yes

Reviewer Expertise:

Conceptual modeling, ontology engineering, Applied Ontology, Enterprise Modeling, Risk Management and Cybersecurity

I confirm that I have read this submission and believe that I have an appropriate level of expertise to state that I do not consider it to be of an acceptable scientific standard, for reasons outlined above.

# Response

**Published:** 2024-09-22

**Authors:** R. Andrew Paskauskas

**Affiliations:** Lithuanian Cybercrime Center of Excellence for Research,Training and Education, Lithuania

The peer reviewer is to be commended for taking the time to consider the paper in question. However, there appears to be a misunderstanding of the purpose and approach. Please note the Peer review criteria (e.g., “Peer review critique No. ??: ) below are identified by the short acronyms PRC1, PRC2, etc; and the author’s reply is identified by “AR1, AR2… etc…: *** **Author's opening statement:** **ORE Criteria ** Open letters are short, peer-reviewed articles … presenting guidelines … or announcing new initiatives. An Open Letter should usually represent the views of an eligible project [in this case H2020 EU-HYBNET ] ... ORE's guidelimnes are: "An Open Letter **should not include new data and analysis** (if it does, a Research Article may be more suitable)…."

**PRC1:** “…the presentation is poorly made so the contributions are unclear.”

**AR1:** The abstract contains an outline of the presentation; the Open Letter format requires no clear contributions – rather, it’s aim is “announcing new initiatives.”

**PRC 2:** “… it is unclear what is intended to be a contribution…”. **A**

**R2.:** Again, the expected contributions apply to a formal research paper. However, this is an Open Letter… ***Peer review critique No. 3***

***(PRC3)*** “…On page 4, the taxonomy of threats comes out of nowhere after mentioning a Google Scholar search.”

**AR3.:** **See the front end of the ABSTRACT:**

1. “A meticulous review of ENISA's Taxonomy is undertaken, specifically in its application to 5G networks and their cybersecurity assets.” 
2. “In the context of hybrid threats, the study categorizes these multifaceted challenges using the established taxonomy.”
3. The inclusion of the relevance of JRC’s taxonomy is spread throughout the rest of the ABSTRACT.

**See the Introduction:**

1. “In Part 1 we take an in-depth look at developing taxonomies and ontologies for analyzing cyberthreats and vulnerabilities related to 5G networks. We begin by introducing ENISA's threat taxonomy for 5G, with categories covering various attack types, actors, and impacts. An overview of ontology development is then provided, explaining how to transform a hierarchy into a full ontology by defining classes, relationships, attributes and using RDF/OWL syntax.”
2. Next in the Introduction: “Focus then shifts to the cyber domain, introducing a high-level taxonomy of potential 5G cyberthreats including signaling attacks, core network threats,”
3. See the Author’s Exposition section, just before the critique’s  complaint of “taxonomy of threats” being introduced as follows:  “…the taxonomy of threats comes out of nowhere…”

**PRC4:** = “No related work section is given.”

**AR4:** The author is not sure what this means. Perhaps a research paper would demand this aspect; however, we are dealing here with a free-form Open Letter.

**PRC 5:** “…The concept of hybrid threat is crucial in the paper but is stated elsewhere ( https://www.hybridcoe.fi/hybrid-threats-as-a-phenomenon/), not discussed in the paper.”

**AR5: ** See the lengthy section on “Hybrid threat ontology: *Conceptual Model” for the requested discussion. Also, see the *Author’s first footnote in the paper: **Footnotes** ^i^ A general overview of what constitutes the idea of hybrid threats is nicely put by The European Centre of Excellence for Countering Hybrid Threats (Hybrid CoE): https://www.hybridcoe.fi/hybrid-threats-as-a-phenomenon/ As well as Reference No. 3, which is a much more technical / scholarly discussion of the topic… The paper refers to the following in footnote 3:

 3. Giannopoulos G, Smith H, Theocharidou M, eds.: The Landscape of Hybrid Threats: A conceptual model. EUR 30585 EN, Publications Office of the European Union, Luxembourg, 2021. Publisher Full Text

**PRC6:** “…The ontology development is confusing. What were the tools employed to make the ontology? What was the methodology? Why not employ a foundational ontology? Why not extend an existing reference domain ontology?”

**AR6:** The paper’s title says it all, “…toward…” a “hybrid threat” ontology. The aim in this “Open Letter” is to “…announce a new initiative…” As far as tools. methodology, etc … we based our first approximation on the ENISA incorporation of the ISO 27005 standard as a first approximation. That’s all it is…”a first approximation…” not a complete ontological famework… WE did this because there is no “foundational ontology” and no “existing reference domain ontology” in the present domain of interest.

**PRC7:** “Figures 1, 2, and 5 are too small.”

**AR 7:** That is true, but if one clicks on the image and uses the standard enlargement tools, the images and lettering come into clear focus. This is why ORE includes appropriate “buttons” for this purpose.

**PRC 8:** “The proposed ontology is not available. Assessing its merits is, therefore, harder.”

**AR8:** A first approximation to the ontology has been proposed, including coding mechanisms linking a Taxonomy to EC cybersecurity compliance requirements.

**PRC 9:** “RDF/OWL snippets are not relevant.  They should be removed. So, Tables 2, 4, 5, 7, and 9 should be removed.”

**AR 9:** I agree that so much code reflects overkill in the narrative. However, what is warranted is a statement of the “relevance” of an RDF/OWL approach to a first approximation to an ontology of interest in this context.

**PRC10.:** Earlier, the reviewer complained, "The concept of hybrid threat is crucial in the paper … not discussed in the paper.”

**AR10:** See response AR5 above…

**PRC11:** As for Figure 2, we have from the reviewer :” Is the "conceptual model" of Figure 2 a contribution?”

**AR11:** Author’s response: “No, it is an elaboration of the model, as already requested …”.

**PRC12:** “Figure 3 has a weird notation. Recommendation: use UML class diagram.”

**AR12:** Consider the guidelines for an Open Letter Identified above at the beginning: “…. An Open Letter should usually represent the views of an eligible project”… in this case it is EU-HYBNET.  Figure 3 arises directly from a H2020 project, as called for in the requirements for an Open Letter.

**PRC13:** “Table 3. ENISA Threat Landscape 2022 report. Is it just repeating the report content?”

**AR13:** It summarises the structure of the taxonomy according to the latest developments, acknowledging that there are enhancements to the ENISA threat taxonomy of December 2020.

**PRC14:** “The paper is unreasonably long. Its contribution is hard to identify and assess.”

**AR14:** Agreed, the paper is much too long. The peer reviewer’s “call for a contribution” in the strict research sense is unrealistic – especially as it is not called for in terms of ORE’s Open Letter concept. What should be assessed by peer reviewers is the “value” of the “announcement of a possible new initiative…”

# Response

**Published:** 2025-02-25

**Authors:** R. Andrew Paskauskas

**Affiliations:** Lithuanian Cybercrime Center of Excellence for Research,Training and Education, Lithuania

Thank you for your review and insightful comments on my paper. I greatly appreciate your time and effort in providing constructive feedback. Below, I address your points and outline the revisions accordingly. ORE should be publishing the new version soon.

 Summary of Key Revisions

 ✅ Clarified that this is an Open Letter and not a scientific research paper

 ✅ Nevertheless, a general overview of sections is provided, employing a narrative-driven approach

 ✅ Added a detailed "Related works" section for significantly improved context and background.

 ✅ Expanded discussion on hybrid threats with sources

 ✅ A detailed ontology developmental process is put forward (four phases, tools employed, rationale…etc.)

 ✅ Made the ontology publicly available on GitHub via a permanent URL for transparency and accessibility

 ✅ Revised figures and visual representations to align with the Open Letter style

 ✅ Removed RDF/XML, redundant tables, and unnecessary technical descriptions to streamline content

 ✅ Figure 2 in previous version has been eliminated

 ✅ Reduced background material to maintain focus on contributions

 ✅ A new set of Tables and Figures has been put forward

 ✅ A plethora of definitions and descriptions resembling a technical report have been eliminated

# Reviewer report: Reviewer response for version 1

**DOI:** 10.21956/openreseurope.18282.r38531

**Published:** 2024-04-22

**Authors:** Leslie F. Sikos

**Affiliations:** 1 Edith Cowan University, Joondalup, Western Australia, Australia

While the proposed ontology seems to aim to contribute to an increasingly important field, there are several indicators that the ontology engineering process was suboptimal.  

 Currently, the proposed ontology is not available, only snippets of it are presented. A URL should be provided that points to the current version, and only include snippets of it when it adds to the discussion.  

 What builds on the state of the art and constitutes original contribution should be presented clearly. The paper has overly detailed description of trivia, such as a part of a taxonomical structure presented both as a list (instead of a tree) and RDF. In addition, there is no reason to provide code examples in RDF/XML, which is too verbose and very difficult to read; instead, RDF/Turtle should be used exclusively throughout. Long code fragments should not be rotated, and common namespace declarations should be omitted altogether from the examples.

 No formal grounding is presented for the ontology (such as in the form of DL axioms), and no indication of integrity checking taken place with reasoners. The computational properties of the formalism are not presented, so the reasoning complexity remains unclear.

 The proposed domain ontology features concepts many of which would apply to more general domains, and are already defined in industry-standard KOSes, which should be reused as per Semantic Web best practices. The alignment with standard KOSes is partial only, such as the ontology is aligned with the ENISA Taxonomy but not with STIX. For instance, a sabotage can be described with STIX’s Attack Motivation Vocabulary, but it is redefined instead.

 Suggested changes:

 • The paper should be shortened to ~20 pages by removing excessive RDF/XML code and providing a URL of the ontology instead (without which the proposed ontology cannot be utilized by implementers). The JRC Taxonomy can be omitted altogether, because its URL is provided, so the readers can look up the file.

 • It has to be clarified what is the ontology based on, i.e., what was used in the knowledge acquisition phase (clear list of KOSes with links to each) and what is the original contribution (which essentially has to be a separate, publicly available ontology file—as per ontology engineering best practice, the proposed ontology should have a permalink namespace URL, such as on purl.org, the abbreviation of which should be used in the examples instead of the symbolic ex:).

 • For the (partial) representation of the taxonomical structure/concept hierarchy, a tree structure should be used (not lists). The topmost concept could be reused from a standard cybersecurity ontology instead of using owl:Thing. Concept names should be in singular, and written in PascalCase, as per ontology engineering best practices (currently inconsistent).

 • Instead of the excessive detailing of the ontology concepts (which are trivial from the file once it is shared), the formal grounding of the ontology needs to be presented with ontology metrics such as class and property count and DL expressivity. This would help implementations, and would make the reasoning complexity clear.

 • Instead of having code fragments of the ontology itself (which does not add value as soon as the ontology URL is included), there should be examples of 5G network traffic under attack represented in RDF using terms of the proposed ontology. The usability of the ontology should be evaluated and demonstrated through a case study with actual cyberattacks in a 5G network, the file of which should be publicly available. This would ensure repeatability. To make the case study complete, complex SPARQL querying and/or automated reasoning should be demonstrated with terms of the ontology.

 • Industry-standard reasoners should be used to check the consistency of the proposed ontology. This would highlight/prevent errors such as the ones in Table 5, where prefixes are used with a concept name in class definitions, making the whole code fragment invalid.

 • The wording should be checked throughout. For example, the caption of Table 9 states “RDF and OWL formats,” despite the OWL code also being RDF. OWL is not a format per se, it is a language (but not a markup language as incorrectly stated); the XML presentation syntax should be referred to correctly (better even, remove the XML examples altogether). “RDF coding” should be written as “RDF representation.” “Comprehend” should be written as “help interpret” (considering the automation this would facilitate, and semantic agents would interpret, and not comprehend, the codified expert knowledge).

 • Formatting should be fixed throughout. For example, in Figure 5, the font size is too small (almost illegible).

Where applicable, are recommendations and next steps explained clearly for others to follow? (Please consider whether others in the research community would be able to implement guidelines or recommendations and/or constructively engage in the debate)

No

Does the article adequately reference differing views and opinions?

Partly

Are all factual statements correct, and are statements and arguments made adequately supported by citations?

Partly

Is the rationale for the Open Letter provided in sufficient detail? (Please consider whether existing challenges in the field are outlined clearly and whether the purpose of the letter is explained)

Partly

Is the Open Letter written in accessible language? (Please consider whether all subject-specific terms, concepts and abbreviations are explained)

Yes

Reviewer Expertise:

Cybersecurity, Formal Knowledge Representation and Automated Reasoning

I confirm that I have read this submission and believe that I have an appropriate level of expertise to confirm that it is of an acceptable scientific standard, however I have significant reservations, as outlined above.

## References

[1] : Decoding 5G security: toward a hybrid threat ontology. *Open Research Europe* .2024;4: 10.12688/openreseurope.16916.1 10.12688/openreseurope.16916.1 41048567 PMC12489459

# Response

**Published:** 2024-05-12

**Authors:** R. Andrew Paskauskas

**Affiliations:** Lithuanian Cybercrime Center of Excellence for Research,Training and Education, Lithuania

**Author response:** Thank you for your thorough review and insightful comments on my paper. I greatly appreciate your time and effort in providing constructive feedback. I would like to acknowledge and address a crucial point raised in your review – the current unavailability of the proposed ontology. As you rightly pointed out, I have not provided a URL or the complete ontology file, as it does not yet exist in its entirety. The primary objective of this paper was to lay the groundwork and take the initial steps towards developing an ontology based on existing taxonomical structures. However, I recognize the importance of presenting a clearly defined and accessible ontology to facilitate its adoption and implementation by the research community. Regarding the state of the art, I want to clarify that my intention was to explore the potential for advancing the field by building upon existing frameworks and taxonomies. The novelty lies in integrating and aligning established resources within the specific context of 5G cybersecurity and hybrid threats. I wholeheartedly agree with your suggestion to condense the paper to approximately 20 pages, as the current length of around 60 pages is certainly excessive. Reducing the paper's length by removing extraneous details and excessive code examples will enhance its clarity and focus on the core contributions. Your recommendations regarding the use of RDF/Turtle syntax, the inclusion of formal grounding through DL axioms, and the alignment with industry-standard Knowledge Organization Systems (KOSes) and frameworks like STIX are well-received. These practices will undoubtedly strengthen the ontology's formal foundation, interoperability, and adherence to best practices in ontology engineering. I also appreciate your guidance on incorporating a case study with actual 5G network traffic under attack, represented using the proposed ontology, and demonstrating complex querying and reasoning capabilities. This practical evaluation will be invaluable in assessing the ontology's usability and potential for real-world applications. I will carefully consider and implement your suggestions, including using industry-standard reasoners for consistency checking, adherence to naming conventions, and addressing any terminology or wording issues. Once again, thank you for your valuable insights and constructive criticism. Your review has highlighted areas for improvement and provided a clear path forward to enhance the quality and impact of my work. I look forward to addressing these points and continuing to refine my research in the field of 5G cybersecurity and ontology development.

# Response

**Published:** 2025-02-25

**Authors:** R. Andrew Paskauskas

**Affiliations:** Lithuanian Cybercrime Center of Excellence for Research,Training and Education, Lithuania

I appreciate the time and effort taken to review this paper. Your detailed feedback has been invaluable in refining the work's clarity, structure, and technical rigour. The comments provided a thoughtful critique, and your insights greatly benefited the revisions.

 Below, I address your key points and outline how they have been incorporated into the revised version, which may appear soon on the ORE website.

**Summary of Key Revisions**

 ✅ Ontology is now publicly accessible via a permanent URL and GitHub.

 ✅ Several code snippets are included that add to the discussion only

 ✅State of the art is presented in ‘Related works’ along with original contribution

 ✅Presentation of trivia and taxonomical lists have been eliminated in favour of trees

 ✅ Removed excessive RDF/XML, standardised RDF/Turtle representation, namespace declarations omitted

 ✅DL axioms, reasoners and computational properties of formalism address reasoning complexity

 ✅ Resolved owl:topObjectProperty issues through extensive Protégé reasoner executions

 ✅ Knowledge acquisition: KOSes are listed and are reused accordingly

 ✅ Ontology is now fully aligned with STIX 2.1 and MITRE ATT&CK standards (+ ENISA 5G Taxonomy)

 ✅ Paper is now ~ 22 pages and URL points to the ontology on GitHub

 ✅ JRC Taxonomy has been omitted and deemed to be irrelevant to the new ontology

 ✅ Knowledge acquisition phase clearly established – KOSes are listed with web links + original contribution noted

 ✅ Permalink namespace URL, i.e., on purl.org is in place

 ✅ ex: eliminated in favour of fght: (5G hybrid threats)

 ✅Tree structure used for partial representation of taxonomical structure

 ✅Top most concept reused from STIX 2.1 = ThreatConcept (consistent with ENISA 5G Taxonomy = Threat)

 ✅ Improved ontology structure (hierarchical trees, concept names singular, written in PascalCase…)

 ✅Ontology metrics (class, property count, etc… ) and DL expressivity addressed in detail

 ✅ Reasoners have been employed (HermiT, ELK, Pellet) - (no use of prefixes in code snippets)

 ✅ Revised terminology for technical accuracy: OWL, RDF, and XML eliminated; comprehend vs. interpret

 ✅ Eliminated unnecessary definitions and redundant detailing

 ✅ A new set of Tables and Figures has been put forward
